# Supplementary material for: CARD14 signaling in intestinal epithelial cells induces intestinal inflammation and intestinal transit delay
Source: EMBO Mol Med. 2025 Oct 23;17(12):3300–28. doi: 10.1038/s44321-025-00321-4 (PMC12686530; doi:10.1038/s44321-025-00321-4)
Supplement: Supplementary file 1 — Table EV1 [file 44321_2025_321_MOESM1_ESM.docx]

**Table EV1 Genes that are differentially expressed between WT and *CARD14*(E138A)^IEC^ mice in IECs of the small intestine.**

A total of 1,289 genes were differentially expressed (FDR < 0.05). Among these, 217 genes were upregulated and 119 genes were downregulated with at least a two-fold change (|log_2_FC| ≥ 1). Of these, 58 genes were upregulated and 19 genes were downregulated with at least a five-fold change (|log_2_FC| ≥ 2.32). Log_2_FC, Log fold change; FDR, False discovery rate

| **Gene** | **LogFC** | **FDR** | **Gene** | **LogFC** | **FDR** |
| --- | --- | --- | --- | --- | --- |
| **Syt8** | 5.896418 | 3.66E-04 | **Sumo3** | -0.26594 | 3.90E-02 |
| **Ripply1** | 5.465938 | 6.33E-05 | **Srp9** | -0.26739 | 4.06E-02 |
| **Dmrta1** | 5.388841 | 6.32E-05 | **Mgat4b** | -0.26883 | 3.28E-02 |
| **Sema3e** | 4.976703 | 7.41E-07 | **Tfdp2** | -0.26936 | 4.59E-02 |
| **Gm12828** | 4.964399 | 5.78E-05 | **Cox14** | -0.27416 | 2.82E-02 |
| **Megf6** | 4.837078 | <1e-10 | **Pdcl3** | -0.27454 | 3.98E-02 |
| **Cxcl1** | 4.566181 | 3.18E-03 | **Erp29** | -0.27527 | 4.74E-02 |
| **Adamts9** | 4.233983 | 4.67E-03 | **Vdac3** | -0.27593 | 3.50E-02 |
| **Apba2** | 4.124732 | 2.69E-03 | **Arpc1a** | -0.27746 | 4.75E-02 |
| **P2rx2** | 4.076278 | 1.23E-03 | **Cib1** | -0.2784 | 8.17E-03 |
| **Ankfn1** | 4.032479 | 4.53E-04 | **Mrpl32** | -0.28057 | 2.95E-02 |
| **Wnt10b** | 3.889644 | 1.70E-04 | **Lamtor1** | -0.28338 | 2.48E-02 |
| **Mir196a-1** | 3.873818 | 6.76E-03 | **Mepce** | -0.28433 | 4.08E-03 |
| **C3** | 3.829354 | 3.86E-06 | **Ywhaq** | -0.28461 | 2.94E-02 |
| **Rasal1** | 3.814416 | <1e-10 | **Nt5c** | -0.285 | 4.18E-02 |
| **Pnpla3** | 3.724921 | <1e-10 | **Immt** | -0.28698 | 1.67E-02 |
| **Muc1** | 3.675777 | 2.17E-03 | **Echdc1** | -0.28729 | 4.92E-02 |
| **Mal** | 3.643791 | <1e-10 | **Pgp** | -0.28849 | 3.24E-02 |
| **Trabd2b** | 3.619929 | 1.67E-02 | **Cops6** | -0.28862 | 4.33E-02 |
| **Dlk1** | 3.562281 | 2.79E-05 | **Eif2a** | -0.28927 | 1.47E-02 |
| **Tff2** | 3.442125 | 2.58E-02 | **Psmd9** | -0.29068 | 3.98E-02 |
| **Sh3tc2** | 3.329291 | 5.90E-04 | **Kdsr** | -0.29091 | 4.08E-02 |
| **Igfals** | 3.18381 | 5.35E-09 | **Gdi2** | -0.29388 | 1.55E-02 |
| **Nox1** | 3.182905 | <1e-10 | **Eif1** | -0.29503 | 2.59E-02 |
| **Cxcl2** | 3.166679 | 6.49E-03 | **Syf2** | -0.29624 | 3.10E-02 |
| **Igf2bp3** | 3.142835 | 2.45E-10 | **Ndufs2** | -0.29682 | 2.91E-02 |
| **Slco5a1** | 3.101058 | 4.38E-03 | **Gm1123** | -0.2969 | 3.92E-02 |
| **Igkv1-117** | 3.069542 | 6.29E-03 | **Sucla2** | -0.29911 | 2.17E-02 |
| **Tnf** | 2.886245 | <1e-10 | **Ilk** | -0.30033 | 1.36E-03 |
| **Cd55b** | 2.876525 | 2.01E-05 | **As3mt** | -0.30046 | 3.53E-02 |
| **Gm36283** | 2.868998 | 3.10E-03 | **Brk1** | -0.3015 | 9.93E-03 |
| **Dnah7b** | 2.865033 | 4.17E-02 | **Eif4b** | -0.30276 | 2.79E-02 |
| **Wnt4** | 2.82041 | 5.57E-08 | **Psma4** | -0.30299 | 2.72E-03 |
| **Loxl2** | 2.81831 | 9.98E-03 | **Higd2a** | -0.30419 | 3.16E-02 |
| **Rims4** | 2.795733 | 3.44E-10 | **Rnf187** | -0.30426 | 1.01E-02 |
| **Csf1** | 2.778307 | 4.65E-10 | **Ap2s1** | -0.30596 | 2.25E-02 |
| **Prickle2** | 2.724271 | 4.76E-04 | **Eif3g** | -0.30607 | 4.03E-02 |
| **Ecscr** | 2.68212 | <1e-10 | **Atg3** | -0.30618 | 4.11E-02 |
| **Ubd** | 2.680443 | 5.48E-05 | **Oaz1** | -0.30637 | 1.13E-02 |
| **Ccl20** | 2.662393 | 3.28E-09 | **Atp5pb** | -0.3073 | 2.40E-02 |
| **Hoxa9** | 2.635593 | 4.65E-03 | **Anp32a** | -0.3107 | 4.28E-02 |
| **Apobec2** | 2.623613 | 1.25E-04 | **Ptms** | -0.31134 | 4.06E-02 |
| **Msx1** | 2.621254 | 1.99E-03 | **Igbp1** | -0.31134 | 1.02E-02 |
| **Capsl** | 2.617041 | 4.91E-03 | **Elob** | -0.31206 | 4.63E-02 |
| **Ighv10-1** | 2.61412 | 2.54E-02 | **Taf10** | -0.31241 | 3.41E-02 |
| **Icam1** | 2.602185 | <1e-10 | **Uaca** | -0.31399 | 2.62E-02 |
| **Etv4** | 2.601675 | <1e-10 | **Dynlrb1** | -0.31427 | 1.15E-02 |
| **Tnip3** | 2.542776 | 6.08E-05 | **Ccdc90b** | -0.31439 | 2.15E-02 |
| **Slc43a3** | 2.529146 | 2.97E-09 | **Suclg2** | -0.31599 | 2.30E-02 |
| **Trim46** | 2.480068 | <1e-10 | **Ndrg3** | -0.31612 | 1.75E-02 |
| **Gm15635** | 2.469245 | 4.84E-03 | **Psmd8** | -0.31613 | 1.65E-02 |
| **Npnt** | 2.372293 | 7.24E-04 | **Polr1d** | -0.3162 | 7.19E-03 |
| **Psg25** | 2.352258 | 7.17E-06 | **Mrps15** | -0.31627 | 4.72E-02 |
| **Slc2a6** | 2.351806 | 1.55E-06 | **Psmb6** | -0.31731 | 8.02E-03 |
| **Sec14l4** | 2.350984 | 9.72E-03 | **Mrpl18** | -0.31755 | 4.32E-02 |
| **Gm14137** | 2.345689 | 7.77E-08 | **Rdh11** | -0.31936 | 2.83E-02 |
| **E230025N22Rik** | 2.343561 | 1.51E-03 | **BC004004** | -0.32094 | 1.90E-02 |
| **Sirpa** | 2.340665 | 1.10E-09 | **Anapc16** | -0.32178 | 2.17E-02 |
| **Loxl3** | 2.305597 | 4.23E-05 | **Dera** | -0.32185 | 1.50E-02 |
| **Hoxaas3** | 2.290322 | 1.17E-02 | **Tbca** | -0.32287 | 3.09E-02 |
| **Micu3** | 2.266009 | 3.01E-04 | **Card10** | -0.32316 | 4.32E-02 |
| **Comp** | 2.226027 | 3.50E-02 | **Cyc1** | -0.32408 | 1.61E-02 |
| **Gm30042** | 2.222882 | 5.60E-03 | **Ppard** | -0.32442 | 4.15E-02 |
| **Rian** | 2.222083 | 2.31E-03 | **Tomm7** | -0.32626 | 2.92E-02 |
| **Hs3st3b1** | 2.215507 | <1e-10 | **Dynll2** | -0.3278 | 8.13E-03 |
| **Zbtb46** | 2.1922 | 6.04E-10 | **Gabarapl2** | -0.32806 | 3.91E-02 |
| **Rhbdl3** | 2.177728 | 1.09E-07 | **Ndufa9** | -0.32847 | 1.10E-02 |
| **Thsd1** | 2.176999 | 5.63E-03 | **Fh1** | -0.33044 | 2.30E-02 |
| **5730507C01Rik** | 2.168312 | 2.98E-05 | **Nudt1** | -0.33152 | 3.84E-02 |
| **Bmp6** | 2.163367 | 1.33E-02 | **Calm3** | -0.33197 | 9.53E-03 |
| **Adgrb1** | 2.143001 | 1.62E-05 | **Pex7** | -0.3321 | 9.70E-03 |
| **Lpl** | 2.138182 | 8.84E-04 | **C1qtnf12** | -0.33225 | 2.98E-02 |
| **Rasgef1a** | 2.135795 | 2.17E-02 | **Mrpl28** | -0.333 | 4.40E-02 |
| **Sycp2l** | 2.13543 | 7.87E-04 | **Arpc3** | -0.33426 | 4.71E-03 |
| **Chst8** | 2.126214 | <1e-10 | **Lsm4** | -0.33478 | 3.62E-02 |
| **Hmox1** | 2.115608 | 1.09E-04 | **Gpd1l** | -0.33511 | 8.57E-03 |
| **Zmynd15** | 2.112308 | <1e-10 | **Cox8a** | -0.33521 | 2.98E-02 |
| **Rerg** | 2.110587 | 1.18E-05 | **Nipsnap3b** | -0.33524 | 1.88E-02 |
| **Gm49493** | 2.092995 | 4.06E-03 | **Oard1** | -0.33629 | 1.93E-02 |
| **Gm45727** | 2.066431 | 2.37E-02 | **Cox6c** | -0.337 | 6.19E-03 |
| **Gm37581** | 2.061563 | 3.93E-02 | **Uqcrc2** | -0.33751 | 1.76E-03 |
| **Meg3** | 2.055338 | 1.26E-03 | **Dhrs7** | -0.33773 | 2.79E-02 |
| **Gsdma2** | 2.043164 | 2.83E-09 | **Ptdss1** | -0.34055 | 5.56E-05 |
| **Gm11948** | 2.041843 | 3.92E-02 | **Ybx1** | -0.3407 | 2.30E-02 |
| **Cap2** | 2.015789 | 1.80E-07 | **Dnttip2** | -0.34117 | 3.63E-02 |
| **Il1rn** | 1.981989 | 5.80E-07 | **Ctnnbip1** | -0.34232 | 4.80E-02 |
| **Lama4** | 1.975996 | 6.93E-03 | **Chmp2a** | -0.34275 | 3.77E-02 |
| **Akr1b8** | 1.974821 | <1e-10 | **Galnt7** | -0.3428 | 6.86E-03 |
| **Hoxa7** | 1.968218 | 3.03E-03 | **Mien1** | -0.34295 | 3.05E-02 |
| **Hoxb3** | 1.962882 | 1.20E-02 | **Pole3** | -0.34313 | 4.26E-02 |
| **Il1r1** | 1.953152 | 2.33E-02 | **Adk** | -0.3435 | 3.42E-02 |
| **Dipk1c** | 1.939008 | 3.35E-04 | **Mrpl27** | -0.34354 | 3.71E-03 |
| **Wnt5b** | 1.934676 | 4.03E-02 | **Eif3k** | -0.34552 | 5.16E-03 |
| **Agbl3** | 1.919449 | 1.61E-03 | **Tmem147** | -0.34581 | 2.14E-02 |
| **Stx11** | 1.899229 | 1.55E-06 | **Cox7c** | -0.34635 | 2.09E-02 |
| **Fut7** | 1.847371 | 3.18E-03 | **Chchd2** | -0.34784 | 4.17E-03 |
| **Hoxa3** | 1.808391 | 4.19E-05 | **Bag1** | -0.34808 | 2.53E-02 |
| **Vcam1** | 1.804836 | 2.11E-02 | **Deptor** | -0.3487 | 2.70E-03 |
| **Pnpla1** | 1.791318 | 3.13E-08 | **Psmb1** | -0.34956 | 7.19E-03 |
| **Pde1c** | 1.790225 | 7.28E-03 | **Atp5c1** | -0.35049 | 1.32E-02 |
| **Ifi44** | 1.786068 | 1.54E-02 | **Mrpl43** | -0.35063 | 1.90E-02 |
| **Sync** | 1.779765 | 9.72E-04 | **Prdx1** | -0.35087 | 1.87E-02 |
| **Cntnap1** | 1.772147 | <1e-10 | **Pgls** | -0.35123 | 1.09E-02 |
| **Oas2** | 1.763577 | 2.68E-04 | **Psma2** | -0.35131 | 2.56E-02 |
| **Gm47863** | 1.754514 | 1.78E-02 | **Sdhb** | -0.35147 | 3.02E-02 |
| **Lbp** | 1.733452 | 1.27E-03 | **Ubl5** | -0.3516 | 2.19E-02 |
| **Col9a3** | 1.725834 | 4.59E-03 | **Atp5b** | -0.35214 | 1.15E-02 |
| **Nfkbid** | 1.721644 | <1e-10 | **Psmb4** | -0.35304 | 1.18E-03 |
| **Abcc4** | 1.689661 | <1e-10 | **Lgals4** | -0.35317 | 2.32E-03 |
| **Nkain1** | 1.688279 | 7.40E-05 | **Rab9** | -0.35358 | 3.49E-03 |
| **Rbpms** | 1.63016 | 1.70E-04 | **Sf3b6** | -0.35365 | 2.12E-02 |
| **Paqr8** | 1.62925 | <1e-10 | **Gzf1** | -0.35394 | 2.51E-02 |
| **Gbp8** | 1.612321 | 6.58E-04 | **Ndufb5** | -0.35426 | 1.08E-02 |
| **Lrrc8e** | 1.589817 | 3.46E-02 | **Ern2** | -0.35529 | 4.08E-03 |
| **Arhgef17** | 1.559224 | 2.23E-02 | **Hsbp1** | -0.35576 | 5.27E-04 |
| **Itih5** | 1.550181 | <1e-10 | **Ndufb3** | -0.35633 | 3.66E-02 |
| **Gm45418** | 1.542838 | 4.92E-02 | **Vti1b** | -0.35686 | 2.12E-02 |
| **Prkar2b** | 1.540043 | 5.10E-03 | **Eci2** | -0.35961 | 2.06E-02 |
| **Cxcl10** | 1.538053 | 1.26E-03 | **Pdk3** | -0.36026 | 1.84E-02 |
| **Sertad4** | 1.537045 | 8.50E-04 | **Atp5g3** | -0.36116 | 8.31E-03 |
| **Il7r** | 1.527308 | 3.86E-03 | **Gid4** | -0.36189 | 4.21E-02 |
| **Pxdn** | 1.523334 | 1.62E-02 | **Cox5a** | -0.36198 | 4.71E-03 |
| **Clca3a1** | 1.501887 | 6.94E-09 | **Faf1** | -0.3631 | 2.83E-02 |
| **Slfn5** | 1.499958 | 3.29E-03 | **Mrpl48** | -0.36354 | 7.14E-03 |
| **Inf2** | 1.498421 | 2.98E-08 | **Aimp1** | -0.36407 | 7.84E-04 |
| **Cbr3** | 1.498217 | 4.92E-02 | **Mrpl9** | -0.36518 | 7.87E-04 |
| **Gsta3** | 1.495369 | <1e-10 | **Ten1** | -0.36538 | 4.78E-02 |
| **Dpysl3** | 1.486645 | <1e-10 | **Suclg1** | -0.366 | 1.84E-02 |
| **Nod2** | 1.4845 | 6.99E-09 | **Gcsh** | -0.36688 | 3.24E-02 |
| **Zfp808** | 1.475578 | 1.18E-03 | **Mrps36** | -0.36795 | 3.09E-02 |
| **Cyba** | 1.469253 | <1e-10 | **Ost4** | -0.36849 | 6.27E-03 |
| **Gm53** | 1.464786 | 1.33E-02 | **Pgm3** | -0.36947 | 3.59E-03 |
| **C030037D09Rik** | 1.448141 | 3.71E-03 | **Atox1** | -0.37047 | 2.18E-02 |
| **Trpv3** | 1.441716 | 1.01E-02 | **Apool** | -0.3705 | 6.58E-03 |
| **Zbtb7c** | 1.429343 | 8.94E-06 | **Psma5** | -0.37059 | 2.06E-02 |
| **Zfp92** | 1.388495 | 2.65E-05 | **Chchd1** | -0.37108 | 4.32E-02 |
| **Gm50069** | 1.386814 | 1.29E-02 | **Rpl18** | -0.37129 | 4.33E-02 |
| **Gm12854** | 1.38524 | 5.78E-05 | **Phgr1** | -0.37146 | 7.03E-04 |
| **Ccdc189** | 1.380066 | 1.04E-02 | **Rpl10** | -0.37213 | 3.30E-02 |
| **Vasn** | 1.373451 | 1.61E-02 | **Uqcr11** | -0.37218 | 3.44E-02 |
| **Syngap1** | 1.369776 | 1.30E-06 | **Ndufb9** | -0.37258 | 1.75E-02 |
| **Slc6a18** | 1.366936 | 1.58E-02 | **Cux1** | -0.37411 | 5.47E-03 |
| **Oas1b** | 1.356671 | 3.43E-04 | **Smagp** | -0.37609 | 1.42E-02 |
| **Epop** | 1.354876 | 9.01E-03 | **Calml4** | -0.37622 | 3.54E-03 |
| **Snhg14** | 1.350656 | 3.06E-04 | **Rab17** | -0.3767 | 9.62E-03 |
| **Il3ra** | 1.350107 | 3.93E-05 | **Tbc1d31** | -0.37757 | 4.63E-02 |
| **Prodh2** | 1.332204 | 3.85E-03 | **Eef2** | -0.3781 | 2.48E-02 |
| **Fgfr4** | 1.331727 | <1e-10 | **H2bc4** | -0.37834 | 2.21E-02 |
| **Rims2** | 1.32047 | 2.21E-02 | **Atp5j** | -0.37854 | 3.99E-03 |
| **Zfp991** | 1.315664 | 3.22E-02 | **Gm11437** | -0.37983 | 4.59E-02 |
| **Tmem184c** | 1.310067 | <1e-10 | **Adgre5** | -0.38002 | 4.63E-02 |
| **Cxcl16** | 1.307732 | 2.77E-07 | **Fam117b** | -0.38257 | 4.12E-02 |
| **Pir** | 1.304421 | 6.34E-04 | **Cpt1a** | -0.38258 | 4.67E-04 |
| **Hpn** | 1.293042 | 6.36E-03 | **Ndufa11** | -0.38359 | 1.15E-02 |
| **Rgs17** | 1.281233 | 3.01E-02 | **Etfa** | -0.38392 | 7.96E-03 |
| **Gm44223** | 1.280543 | 1.95E-04 | **Hibch** | -0.38424 | 3.47E-02 |
| **Pla2g5** | 1.27345 | 1.33E-02 | **Cyb5a** | -0.38459 | 2.32E-02 |
| **Gem** | 1.270969 | 3.66E-02 | **Cox5b** | -0.38495 | 7.90E-03 |
| **Rhbdf1** | 1.256738 | <1e-10 | **Txndc17** | -0.38512 | 1.23E-02 |
| **9530056K15Rik** | 1.25342 | 4.53E-03 | **Mrpl16** | -0.38533 | 4.82E-02 |
| **Smarcd3** | 1.250455 | 2.38E-02 | **Clta** | -0.38545 | 2.01E-04 |
| **Trpm6** | 1.243121 | 2.48E-02 | **Edf1** | -0.38554 | 4.03E-02 |
| **Fyb2** | 1.23893 | 2.56E-04 | **Mea1** | -0.38879 | 2.29E-02 |
| **Gpr17** | 1.234764 | 2.49E-04 | **Btf3** | -0.38892 | 3.18E-03 |
| **Adora1** | 1.234396 | 2.35E-04 | **Mrps23** | -0.38942 | 1.93E-02 |
| **Iqcd** | 1.231177 | 3.87E-02 | **Pfdn1** | -0.3899 | 9.93E-03 |
| **Il18r1** | 1.229034 | 2.95E-02 | **Cops9** | -0.39034 | 4.17E-04 |
| **Flt3l** | 1.22808 | 2.20E-04 | **Aldh9a1** | -0.39038 | 1.76E-02 |
| **Tmem198** | 1.227811 | 1.50E-02 | **Mrps21** | -0.39186 | 2.40E-02 |
| **Macc1** | 1.193936 | 7.35E-03 | **Ndufv2** | -0.3925 | 7.84E-04 |
| **Lama5** | 1.190489 | 1.09E-04 | **D630039A03Rik** | -0.39277 | 1.10E-02 |
| **Clca3a2** | 1.184684 | 2.86E-07 | **Chchd3** | -0.39291 | 1.33E-02 |
| **Gm11723** | 1.174068 | 3.94E-02 | **Ndufb11** | -0.39488 | 8.19E-04 |
| **Syne4** | 1.164608 | 3.54E-03 | **Fbp2** | -0.39609 | 8.90E-03 |
| **4930402H24Rik** | 1.155777 | 3.14E-05 | **Ndufa6** | -0.39688 | 2.73E-02 |
| **Cep126** | 1.155333 | 2.17E-02 | **Uqcrfs1** | -0.39756 | 1.75E-02 |
| **Ap1s2** | 1.153091 | 1.57E-02 | **Fgfr3** | -0.39832 | 4.59E-02 |
| **Thbs1** | 1.152839 | 1.04E-04 | **Pafah1b3** | -0.39942 | 8.07E-03 |
| **Trim35** | 1.151455 | 7.87E-04 | **Abhd17a** | -0.40001 | 2.79E-02 |
| **Itga5** | 1.13282 | 2.36E-03 | **Mrpl34** | -0.40032 | 7.83E-03 |
| **Chst11** | 1.118348 | 1.70E-04 | **Mpc2** | -0.40065 | 1.31E-02 |
| **Bcam** | 1.111884 | 5.66E-06 | **Atp5l** | -0.40072 | 3.18E-03 |
| **Lrrc3** | 1.111863 | 1.46E-03 | **Rcan3** | -0.40121 | 4.66E-02 |
| **Igsf9b** | 1.106925 | <1e-10 | **Atp5d** | -0.40172 | 5.17E-03 |
| **Gm11735** | 1.106437 | 7.40E-03 | **Dmbt1** | -0.40221 | 9.01E-03 |
| **Cd44** | 1.100242 | 1.36E-07 | **Gm10080** | -0.40259 | 2.38E-02 |
| **Igdcc4** | 1.09958 | 3.93E-05 | **Atp5md** | -0.40324 | 8.03E-03 |
| **Gm17193** | 1.095705 | 2.92E-02 | **Decr1** | -0.40389 | 4.78E-02 |
| **Pdia5** | 1.094575 | <1e-10 | **Atp5h** | -0.40421 | 1.98E-03 |
| **Rhbdl2** | 1.093937 | 2.31E-04 | **Ndufb7** | -0.40468 | 2.09E-03 |
| **Nup210l** | 1.079568 | 2.58E-02 | **Gm5436** | -0.40504 | 1.50E-02 |
| **Hrh1** | 1.078379 | 3.09E-03 | **Ppdpf** | -0.40653 | 3.40E-02 |
| **Lrp8** | 1.077165 | 1.15E-03 | **Ndufa5** | -0.4071 | 1.90E-02 |
| **Arhgef10l** | 1.075519 | 2.10E-05 | **Lamtor2** | -0.40742 | 3.19E-02 |
| **Mdfic** | 1.073715 | 4.75E-03 | **Esd** | -0.41097 | 9.62E-04 |
| **Ctrl** | 1.072851 | 4.67E-03 | **Eif3f** | -0.41178 | 1.75E-02 |
| **Mmp28** | 1.070329 | 2.55E-07 | **Pfdn6** | -0.41184 | 9.10E-03 |
| **Zfp275** | 1.068823 | 1.32E-08 | **Cltb** | -0.41194 | 9.82E-04 |
| **Slc6a7** | 1.067483 | 7.43E-04 | **Pithd1** | -0.4124 | 3.19E-03 |
| **Gm48293** | 1.061998 | 4.35E-02 | **Mrpl52** | -0.41332 | 4.23E-02 |
| **Ccdc122** | 1.061206 | 1.76E-03 | **Cd63** | -0.41347 | 2.29E-03 |
| **Scn2b** | 1.06049 | 2.03E-03 | **Zfp472** | -0.41382 | 4.11E-02 |
| **Gm38190** | 1.056986 | 2.37E-02 | **Enox2** | -0.41723 | 3.59E-03 |
| **Gbp9** | 1.053865 | 1.20E-02 | **Mrpl30** | -0.41744 | 6.01E-03 |
| **Cpe** | 1.047298 | 2.47E-06 | **Rpl27** | -0.41786 | 3.84E-02 |
| **Gm35551** | 1.039086 | 8.76E-03 | **Nop53** | -0.41905 | 9.53E-03 |
| **Oas1g** | 1.037225 | 6.73E-04 | **Ndufa8** | -0.41936 | 1.66E-03 |
| **B9d1** | 1.024756 | 2.65E-02 | **Ndufa2** | -0.42111 | 1.36E-03 |
| **Gm44321** | 1.018787 | 4.17E-02 | **Glrx5** | -0.42263 | 2.48E-02 |
| **H2-T24** | 1.017774 | 1.86E-02 | **Cox4i1** | -0.42354 | 4.23E-03 |
| **Mx2** | 1.016912 | 2.83E-03 | **Gmnn** | -0.42361 | 7.78E-03 |
| **Tnfrsf9** | 1.015706 | 1.68E-02 | **Ndufs4** | -0.42547 | 9.50E-04 |
| **Gm43681** | 1.014743 | 1.67E-02 | **Dap** | -0.4259 | 2.58E-02 |
| **Mreg** | 1.011201 | 1.68E-04 | **Hint1** | -0.42763 | 1.44E-03 |
| **Klhdc8b** | 1.007194 | 2.08E-03 | **S100a10** | -0.4278 | 3.50E-02 |
| **Fam129c** | 1.007013 | 1.90E-02 | **Ndufv3** | -0.42848 | 9.88E-03 |
| **Plxna1** | 1.003659 | <1e-10 | **Aldoa** | -0.42873 | 1.96E-02 |
| **Fam20c** | 0.998803 | 4.67E-02 | **Mrpl14** | -0.42884 | 3.33E-02 |
| **Gbp6** | 0.984663 | 5.16E-04 | **Nxt2** | -0.42937 | 4.56E-02 |
| **Sting1** | 0.97273 | 7.33E-05 | **Naca** | -0.43001 | 5.19E-03 |
| **Il20rb** | 0.971686 | 1.02E-02 | **Tmlhe** | -0.43044 | 6.39E-03 |
| **Map3k14** | 0.969645 | <1e-10 | **Ppp1r14d** | -0.43228 | 2.75E-02 |
| **Eml5** | 0.967936 | 3.83E-02 | **Cox7a2** | -0.43373 | 7.72E-03 |
| **Agrn** | 0.963969 | <1e-10 | **Naxe** | -0.43376 | 2.48E-02 |
| **Pglyrp1** | 0.961751 | 2.55E-07 | **Atp5o** | -0.4354 | 2.96E-03 |
| **Ddx60** | 0.961005 | 1.15E-03 | **Ggact** | -0.43785 | 5.61E-08 |
| **Cd14** | 0.960151 | 2.99E-05 | **Anapc13** | -0.4379 | 7.89E-03 |
| **Mapk11** | 0.957762 | 1.30E-02 | **Timm8b** | -0.43793 | 1.03E-02 |
| **Tnfaip3** | 0.950758 | 8.16E-09 | **Rpl3** | -0.43824 | 4.99E-02 |
| **S100a11** | 0.948463 | <1e-10 | **Mrpl42** | -0.44035 | 3.59E-03 |
| **Ppl** | 0.947666 | 1.23E-10 | **Pdcd4** | -0.44121 | 2.52E-03 |
| **Nbea** | 0.944456 | 4.17E-06 | **Dgcr6** | -0.44237 | 2.48E-02 |
| **Banp** | 0.939732 | 2.47E-03 | **Eef1g** | -0.44278 | 1.14E-02 |
| **Gm26532** | 0.937654 | 4.08E-03 | **Uqcc2** | -0.4428 | 6.72E-03 |
| **Gm50010** | 0.936436 | 1.58E-02 | **Kxd1** | -0.4431 | 4.94E-03 |
| **Plxnb1** | 0.934821 | 3.62E-07 | **Ndufb10** | -0.44331 | 1.90E-02 |
| **Fam189a2** | 0.934762 | 2.45E-06 | **Acads** | -0.44479 | 1.27E-02 |
| **Me1** | 0.930383 | 1.71E-04 | **Ccl6** | -0.44565 | 9.93E-03 |
| **Phf11d** | 0.926506 | 1.50E-03 | **Cox7b** | -0.44621 | 6.72E-03 |
| **Gm20219** | 0.92487 | 1.15E-03 | **Atp5g2** | -0.44654 | 1.65E-03 |
| **Eif4e3** | 0.924209 | 3.15E-04 | **Cox6b1** | -0.44886 | 3.08E-04 |
| **Oas3** | 0.921407 | 3.32E-03 | **Bcas1** | -0.45026 | 1.20E-02 |
| **Tfrc** | 0.918925 | 2.29E-04 | **Rps9** | -0.45088 | 2.32E-02 |
| **Abcc1** | 0.918198 | 4.26E-09 | **Tinag** | -0.45367 | 9.28E-03 |
| **Noxo1** | 0.911794 | 3.20E-08 | **Dcxr** | -0.45558 | 4.83E-04 |
| **Nid2** | 0.909781 | 4.19E-05 | **Micos13** | -0.45567 | 6.75E-03 |
| **Emp2** | 0.907891 | 2.49E-03 | **Cd63-ps** | -0.4589 | 2.54E-02 |
| **Il7** | 0.907783 | 4.71E-03 | **Rpl10a** | -0.45939 | 3.71E-02 |
| **Tmem176a** | 0.902904 | 2.14E-08 | **Ethe1** | -0.4594 | 1.57E-03 |
| **Trim30a** | 0.902224 | 3.92E-02 | **Ndufb1-ps** | -0.46 | 9.93E-03 |
| **Nfkbie** | 0.899647 | 8.34E-04 | **Zfp358** | -0.46044 | 4.63E-02 |
| **Nfkbia** | 0.896476 | 4.40E-08 | **Cth** | -0.4609 | 5.16E-03 |
| **Nfkb2** | 0.895574 | <1e-10 | **Rpl11** | -0.46148 | 1.84E-02 |
| **Parp3** | 0.894943 | 3.43E-04 | **9430038I01Rik** | -0.46254 | 2.20E-02 |
| **Tmem176b** | 0.894278 | <1e-10 | **Hsd17b10** | -0.46743 | 6.36E-03 |
| **Tmem132a** | 0.890784 | 5.54E-03 | **Zfp954** | -0.46755 | 2.30E-03 |
| **Sema4c** | 0.888396 | 1.86E-02 | **Oit1** | -0.47043 | 2.88E-02 |
| **Slc22a15** | 0.883099 | 1.97E-07 | **Gm19810** | -0.47049 | 3.33E-02 |
| **Gm14286** | 0.876867 | 4.59E-02 | **0610012G03Rik** | -0.47081 | 1.64E-02 |
| **Abcc8** | 0.872174 | 2.18E-02 | **Rpl30** | -0.47137 | 6.00E-03 |
| **Mboat1** | 0.868065 | 4.57E-03 | **Rsph3b** | -0.472 | 4.41E-02 |
| **Gm47218** | 0.867045 | 1.51E-02 | **Ndufa3** | -0.47203 | 8.40E-05 |
| **Gm42547** | 0.861837 | 5.25E-03 | **Hmgcl** | -0.47221 | 5.69E-04 |
| **Oas1c** | 0.861276 | 2.53E-02 | **Rpl15** | -0.47238 | 1.57E-02 |
| **Cyp4f17** | 0.85972 | 1.43E-02 | **Pipox** | -0.47243 | 2.11E-02 |
| **Rnf213** | 0.857867 | 9.83E-05 | **Eif3h** | -0.4751 | 1.33E-03 |
| **Iqank1** | 0.857604 | 4.70E-02 | **Atp5g1** | -0.47686 | 1.93E-03 |
| **Nmrk1** | 0.856652 | 4.54E-06 | **Abhd11os** | -0.47803 | 2.58E-02 |
| **Tgfbr2** | 0.850873 | 5.45E-08 | **Rap1gap** | -0.47846 | 1.30E-02 |
| **Mfge8** | 0.848963 | 2.29E-04 | **Msra** | -0.47878 | 3.98E-02 |
| **C2** | 0.846373 | 5.27E-04 | **Myorg** | -0.47904 | 2.62E-02 |
| **Dusp14** | 0.845249 | 1.65E-03 | **Atpif1** | -0.48071 | 1.36E-03 |
| **H2-Q6** | 0.844682 | 4.77E-03 | **Ndufb4** | -0.48124 | 1.14E-02 |
| **Stard5** | 0.842011 | 1.08E-05 | **Gm5881** | -0.48305 | 1.50E-02 |
| **Lacc1** | 0.838769 | 3.81E-03 | **Cst3** | -0.48327 | 7.54E-05 |
| **Arnt2** | 0.837946 | 9.82E-04 | **Tma7** | -0.48406 | 9.72E-03 |
| **Rtp4** | 0.835917 | 1.84E-02 | **Rplp0** | -0.48409 | 3.92E-02 |
| **Zfr2** | 0.831616 | 8.61E-08 | **Gm13597** | -0.48433 | 4.62E-02 |
| **Adgrl2** | 0.829722 | 7.77E-08 | **Pccb** | -0.48526 | 7.48E-08 |
| **Dpy19l1** | 0.827729 | 1.16E-04 | **Gm4332** | -0.48542 | 2.46E-02 |
| **Muc4** | 0.826621 | 9.72E-04 | **Eif3i** | -0.48615 | 7.20E-03 |
| **Celsr1** | 0.818271 | 5.37E-04 | **Rpl7** | -0.48669 | 3.18E-03 |
| **Iffo2** | 0.817768 | 9.62E-04 | **Rpl37** | -0.48707 | 9.93E-03 |
| **Slc11a2** | 0.814556 | 7.73E-05 | **Cdkn2d** | -0.48913 | 1.58E-02 |
| **6720427I07Rik** | 0.811677 | 2.30E-02 | **Gm11964** | -0.49062 | 2.78E-02 |
| **Tfcp2l1** | 0.808835 | 6.36E-10 | **Uqcrh** | -0.49063 | 1.15E-03 |
| **Ptpn13** | 0.80572 | 1.75E-02 | **Apopt1** | -0.49218 | 6.32E-03 |
| **Dhx58** | 0.804655 | 1.84E-02 | **Taf7** | -0.49236 | 1.17E-02 |
| **Pls3** | 0.803448 | 6.29E-03 | **Rplp1** | -0.49237 | 1.56E-02 |
| **Rbms1** | 0.801478 | 4.29E-06 | **Selenom** | -0.49277 | 7.61E-03 |
| **Plxna3** | 0.799626 | 3.24E-02 | **Smim22** | -0.49364 | 6.52E-03 |
| **Anxa5** | 0.799573 | 1.87E-05 | **Mrps24** | -0.49399 | 1.36E-05 |
| **Herc6** | 0.797541 | 8.74E-03 | **Rpl6** | -0.49411 | 2.18E-02 |
| **Mpzl2** | 0.795031 | 7.33E-06 | **Rps13** | -0.49493 | 1.53E-02 |
| **F8a** | 0.78564 | 3.94E-05 | **Arhgap31** | -0.49734 | 8.16E-03 |
| **Tmed8** | 0.783056 | 2.30E-02 | **Xxylt1** | -0.49739 | 4.38E-02 |
| **Gm44974** | 0.78298 | 7.89E-03 | **Rpl22** | -0.49898 | 1.66E-02 |
| **Stx1a** | 0.774224 | 1.30E-02 | **Spryd7** | -0.50019 | 1.50E-02 |
| **Rbm43** | 0.772115 | 4.71E-03 | **Zfp467** | -0.50284 | 4.41E-02 |
| **Zc3h12a** | 0.769447 | 1.31E-05 | **Rpl4** | -0.50289 | 1.24E-02 |
| **Ptbp2** | 0.768891 | 2.79E-05 | **Hscb** | -0.50297 | 1.58E-02 |
| **Pgap1** | 0.768805 | 7.43E-05 | **Fam174b** | -0.50303 | 1.49E-02 |
| **Glb1l** | 0.765442 | 3.00E-02 | **Atp5j2** | -0.50342 | 7.19E-04 |
| **A930001C03Rik** | 0.765362 | 1.84E-02 | **Atp5k-ps2** | -0.50486 | 1.90E-02 |
| **Nipal2** | 0.762815 | 2.51E-04 | **Mrpl23** | -0.50535 | 8.42E-03 |
| **Gbp7** | 0.761934 | 2.19E-02 | **Rpl23a-ps3** | -0.50633 | 4.08E-02 |
| **Btc** | 0.757795 | 5.19E-03 | **Eef1a1** | -0.50649 | 5.01E-03 |
| **Ifitm3** | 0.753271 | 4.21E-02 | **Lrrc26** | -0.50882 | 5.01E-03 |
| **Cd274** | 0.752932 | 2.66E-02 | **Shank2** | -0.50937 | 3.98E-02 |
| **Zdhhc17** | 0.748117 | 2.25E-07 | **Rps2** | -0.50963 | 3.95E-02 |
| **Slfn2** | 0.743423 | 1.90E-02 | **Rpl19** | -0.51147 | 8.72E-03 |
| **5730480H06Rik** | 0.741611 | 3.01E-04 | **Gm10250** | -0.51196 | 5.56E-04 |
| **Abcc3** | 0.732423 | 1.38E-05 | **Ndufaf8** | -0.51245 | 1.03E-02 |
| **Xkr8** | 0.729142 | 1.91E-02 | **Hepacam2** | -0.51295 | 1.55E-06 |
| **Crlf2** | 0.726502 | 1.46E-03 | **Eef1b2** | -0.51397 | 6.39E-03 |
| **Arhgap8** | 0.724357 | 1.33E-02 | **Pyroxd2** | -0.51505 | 3.89E-02 |
| **Sp110** | 0.71862 | 1.94E-02 | **Plin2** | -0.51511 | 3.01E-02 |
| **Fzd7** | 0.718571 | 2.91E-06 | **Rps21** | -0.51544 | 1.59E-02 |
| **2900005J15Rik** | 0.718432 | 3.71E-02 | **Rpl7a** | -0.51621 | 5.63E-03 |
| **Casp4** | 0.717437 | 3.82E-04 | **Ccne2** | -0.51651 | 3.25E-02 |
| **Arrb2** | 0.717314 | 5.27E-03 | **Fbxl15** | -0.51666 | 2.51E-02 |
| **Cytip** | 0.715039 | 2.93E-02 | **Mrpl13** | -0.51691 | 1.95E-04 |
| **Traf3** | 0.712846 | <1e-10 | **Rpl39** | -0.51719 | 7.54E-03 |
| **Fads3** | 0.71248 | 2.37E-02 | **Otc** | -0.51818 | 1.34E-02 |
| **Nfatc2** | 0.712352 | 2.87E-02 | **Gm10039** | -0.5183 | 7.71E-03 |
| **Nt5dc3** | 0.704816 | 4.33E-02 | **Uqcrb** | -0.51945 | 4.37E-05 |
| **Hivep3** | 0.703181 | 2.69E-02 | **Cmbl** | -0.52002 | 3.71E-03 |
| **Mmp14** | 0.699183 | 3.72E-03 | **Dpf3** | -0.52009 | 1.62E-02 |
| **1700066B19Rik** | 0.698222 | 1.25E-02 | **Dbi** | -0.52012 | 3.26E-05 |
| **Nlrc5** | 0.69268 | 1.74E-02 | **Slc51b** | -0.52236 | 6.27E-03 |
| **Rc3h1** | 0.690939 | 6.38E-06 | **Coa4** | -0.52449 | 2.31E-03 |
| **Oasl2** | 0.690698 | 3.95E-02 | **Rps23** | -0.52512 | 9.53E-03 |
| **Cxcl11** | 0.689739 | 2.63E-03 | **Rps15a** | -0.52573 | 2.32E-02 |
| **Dusp4** | 0.689532 | 1.63E-02 | **Endog** | -0.52601 | 1.13E-02 |
| **Hdac7** | 0.689505 | 3.40E-02 | **Rpl23a** | -0.52792 | 3.98E-02 |
| **Rfx5** | 0.684486 | 3.25E-09 | **Rpl18a** | -0.52891 | 7.66E-03 |
| **Msi1** | 0.680074 | 2.28E-02 | **2410006H16Rik** | -0.52905 | 3.04E-02 |
| **Tnfrsf1b** | 0.676649 | 3.29E-03 | **Prelid2** | -0.52999 | 3.53E-02 |
| **Hspg2** | 0.676508 | 1.07E-02 | **Rps10-ps1** | -0.5301 | 3.60E-02 |
| **Fbxw17** | 0.675673 | 5.14E-05 | **Nsa2** | -0.53089 | 5.03E-04 |
| **Cyren** | 0.675036 | 3.35E-02 | **Pcbd2** | -0.53184 | 1.03E-02 |
| **Fuz** | 0.674488 | 2.65E-03 | **Rps26** | -0.53233 | 2.35E-02 |
| **Slc13a2** | 0.674109 | 2.44E-03 | **Rps29** | -0.53316 | 1.26E-03 |
| **Col20a1** | 0.671045 | 2.46E-02 | **Commd1** | -0.5337 | 3.69E-03 |
| **Cd9** | 0.670497 | 2.65E-02 | **Sesn2** | -0.53434 | 2.35E-04 |
| **Celf5** | 0.670432 | 1.59E-02 | **Rack1** | -0.53443 | 1.55E-02 |
| **Ripk2** | 0.668069 | 2.21E-02 | **Gm6863** | -0.53582 | 4.65E-03 |
| **Rab12** | 0.666014 | 7.06E-03 | **H3f3a** | -0.5361 | 2.35E-05 |
| **Rnf144b** | 0.661483 | 3.24E-02 | **Kiz** | -0.53654 | 1.60E-02 |
| **H2-Q7** | 0.65468 | 3.25E-02 | **Fut4** | -0.53741 | 1.18E-03 |
| **Itgb6** | 0.651535 | 6.72E-03 | **Smco4** | -0.53888 | 1.50E-03 |
| **Gm12940** | 0.650119 | 2.20E-02 | **Triap1** | -0.54023 | 2.98E-05 |
| **Cdk17** | 0.649072 | 1.96E-03 | **Rpl8** | -0.54235 | 7.28E-03 |
| **Dpm1** | 0.648842 | 6.64E-03 | **Pm20d1** | -0.54267 | 5.16E-03 |
| **Marcksl1** | 0.648478 | 4.26E-03 | **Rpl12** | -0.54276 | 2.20E-02 |
| **Piezo1** | 0.647556 | 4.90E-03 | **Rps28** | -0.54297 | 7.18E-03 |
| **Gm9926** | 0.645896 | 3.92E-02 | **Acot13** | -0.54319 | 2.64E-02 |
| **Gm20559** | 0.644725 | 4.63E-04 | **Ndufa4** | -0.5446 | 1.90E-04 |
| **Alpk1** | 0.64291 | 3.65E-02 | **Rep15** | -0.54636 | 8.35E-03 |
| **Gm37893** | 0.642581 | 1.95E-02 | **Gm3336** | -0.54737 | 1.38E-02 |
| **Sfxn3** | 0.63325 | 5.74E-03 | **Rps5** | -0.54805 | 1.17E-02 |
| **Fam129b** | 0.621576 | 1.04E-02 | **Rpl37a** | -0.54929 | 8.08E-03 |
| **Htra2** | 0.618829 | 8.95E-06 | **Rpl21** | -0.55078 | 9.15E-04 |
| **Tcaf1** | 0.618327 | 9.56E-03 | **H2bc21** | -0.55095 | 4.23E-02 |
| **Megf8** | 0.617699 | 8.04E-05 | **Tmppe** | -0.55336 | 3.02E-02 |
| **Il6st** | 0.617076 | 8.85E-07 | **Gtf2b** | -0.55449 | 9.40E-06 |
| **Poglut3** | 0.616915 | 3.41E-04 | **Scp2** | -0.55899 | 1.36E-03 |
| **Kctd18** | 0.614998 | 3.03E-02 | **Cox7a2l** | -0.56016 | 2.23E-04 |
| **4930581F22Rik** | 0.614082 | 8.76E-03 | **Rplp2** | -0.56102 | 9.03E-03 |
| **Slc45a4** | 0.613946 | 6.04E-04 | **Rps4x** | -0.56165 | 1.32E-02 |
| **Csad** | 0.612016 | 1.35E-03 | **Spdef** | -0.56239 | 1.32E-02 |
| **Afap1** | 0.610363 | 3.49E-02 | **Rps18** | -0.56536 | 1.59E-02 |
| **Zfp950** | 0.6102 | 1.23E-02 | **Rpl23** | -0.56537 | 2.52E-03 |
| **Rpgrip1l** | 0.607165 | 8.42E-03 | **Rpl36** | -0.56563 | 1.75E-02 |
| **Myef2** | 0.603533 | 2.83E-02 | **Tpt1-ps3** | -0.56608 | 1.69E-03 |
| **Tchp** | 0.602311 | 5.16E-03 | **Rps27a** | -0.56875 | 4.55E-03 |
| **Whrn** | 0.601685 | 1.06E-04 | **Rpl29** | -0.57113 | 8.92E-03 |
| **Stat1** | 0.601527 | 1.65E-03 | **Uqcrq** | -0.57153 | 4.17E-04 |
| **Man2a2** | 0.601199 | 1.50E-02 | **Pfdn5** | -0.57524 | 2.92E-04 |
| **Xaf1** | 0.597051 | 3.62E-02 | **Etfb** | -0.57541 | 6.36E-03 |
| **Prnp** | 0.595896 | 4.93E-02 | **Dnajc28** | -0.57642 | 4.32E-02 |
| **Tead1** | 0.595678 | 9.06E-03 | **Rps15** | -0.5769 | 4.37E-03 |
| **Aplp2** | 0.589144 | 1.94E-08 | **Scp2-ps2** | -0.57948 | 1.24E-02 |
| **Klf7** | 0.58895 | 2.08E-03 | **Atp5mpl** | -0.58064 | 1.34E-05 |
| **Ccdc66** | 0.58445 | 2.67E-03 | **Ndufs6** | -0.58112 | 2.98E-05 |
| **Pik3r3** | 0.582608 | 8.60E-03 | **Rpl36a** | -0.58284 | 1.84E-02 |
| **Eppk1** | 0.581898 | 1.90E-04 | **Rps16** | -0.58503 | 8.44E-03 |
| **A730017L22Rik** | 0.580104 | 7.96E-03 | **Rps11** | -0.58515 | 2.29E-03 |
| **Tmem161b** | 0.579666 | 5.09E-03 | **Rpl13** | -0.58542 | 8.84E-03 |
| **Csnk1e** | 0.579665 | 5.75E-05 | **Rps6** | -0.58552 | 1.84E-02 |
| **Kif12** | 0.579199 | 3.20E-02 | **Kcnk6** | -0.58568 | 3.01E-04 |
| **Ppp4r1l-ps** | 0.576994 | 1.46E-02 | **Fau** | -0.58617 | 6.46E-05 |
| **Samd9l** | 0.576271 | 3.62E-03 | **Aamdc** | -0.59128 | 3.34E-03 |
| **Fam219a** | 0.576049 | 2.30E-02 | **H2ax** | -0.59182 | 3.05E-02 |
| **Lpar6** | 0.574969 | 7.28E-03 | **Pde8a** | -0.59266 | 4.98E-02 |
| **9530082P21Rik** | 0.573698 | 4.94E-03 | **Rpl34** | -0.59593 | 5.03E-03 |
| **Ikbke** | 0.572953 | 1.62E-05 | **Camkk2** | -0.59731 | 4.32E-02 |
| **Rras** | 0.572815 | 9.88E-03 | **Psd3** | -0.59755 | 1.04E-02 |
| **Id2** | 0.57064 | 1.03E-03 | **Rps3a1** | -0.59781 | 3.77E-03 |
| **Map1s** | 0.570367 | 1.25E-04 | **Creb3l4** | -0.59983 | 9.07E-04 |
| **Atp11c** | 0.56933 | 1.93E-02 | **Pxmp2** | -0.60032 | 2.48E-02 |
| **Lamc2** | 0.563551 | 4.33E-02 | **Rpl14-ps1** | -0.60091 | 2.04E-02 |
| **Per3** | 0.560104 | 2.43E-02 | **Mfsd4a** | -0.60338 | 2.51E-03 |
| **Rhbdf2** | 0.559964 | 3.35E-10 | **Gm9794** | -0.60512 | 9.38E-04 |
| **Zfp984** | 0.559187 | 7.50E-03 | **Gm15427** | -0.60691 | 1.64E-02 |
| **Slc38a9** | 0.554583 | 2.14E-02 | **Rpl9** | -0.60931 | 2.37E-03 |
| **Trim34a** | 0.552641 | 2.48E-02 | **Rps17** | -0.60999 | 2.05E-03 |
| **Herpud1** | 0.549454 | 2.51E-07 | **Gm6419** | -0.61088 | 4.23E-02 |
| **Gdpd5** | 0.549266 | 4.19E-03 | **Mpst** | -0.61158 | 9.93E-03 |
| **2610507I01Rik** | 0.548641 | 1.01E-02 | **Tpd52-ps** | -0.61236 | 9.98E-03 |
| **Paxbp1** | 0.54803 | 3.37E-03 | **Map2k6** | -0.61271 | 2.48E-02 |
| **Myo9b** | 0.547625 | 1.80E-04 | **Rpl14** | -0.61308 | 7.51E-03 |
| **Hr** | 0.547301 | 1.13E-02 | **Rps20** | -0.61309 | 7.83E-03 |
| **Sdhaf1** | 0.54591 | 6.01E-03 | **Galnt5** | -0.61349 | 6.38E-03 |
| **Apobec3** | 0.54433 | 2.80E-02 | **Gas6** | -0.6158 | 2.19E-04 |
| **Lrch1** | 0.542121 | 4.59E-02 | **Polr2k** | -0.61612 | 5.50E-03 |
| **Cep162** | 0.541145 | 5.33E-04 | **Tmem86b** | -0.61946 | 6.36E-03 |
| **D930048N14Rik** | 0.540959 | 2.48E-02 | **Rps3** | -0.62197 | 1.36E-03 |
| **Trim3** | 0.53908 | 1.25E-02 | **Tspan6** | -0.62463 | 4.84E-02 |
| **Tm2d3** | 0.538597 | 2.03E-03 | **Rpl28** | -0.62816 | 2.54E-03 |
| **Lgals3bp** | 0.536089 | 3.03E-02 | **Adtrp** | -0.62866 | 4.17E-04 |
| **Ctsh** | 0.531206 | 6.07E-05 | **Rps8** | -0.63042 | 1.76E-03 |
| **Izumo4** | 0.525587 | 3.54E-02 | **Rps12** | -0.63204 | 5.93E-03 |
| **Rab8b** | 0.523777 | 1.23E-02 | **Rpl37rt** | -0.63329 | 1.18E-04 |
| **Mmp15** | 0.523582 | 1.50E-02 | **Rps14** | -0.63499 | 7.99E-04 |
| **Gm20300** | 0.517347 | 3.22E-02 | **Rps7** | -0.63946 | 2.18E-03 |
| **Fkbp5** | 0.512745 | 8.72E-03 | **Uqcr10** | -0.64 | 2.14E-05 |
| **Atp11a** | 0.511406 | 1.50E-02 | **Rpl5** | -0.64085 | 1.33E-03 |
| **Trit1** | 0.507015 | 7.65E-03 | **Tcea3** | -0.64217 | 8.89E-05 |
| **Parp11** | 0.506632 | 4.65E-03 | **Rps24** | -0.64225 | 3.07E-03 |
| **Prpf39** | 0.503534 | 1.90E-02 | **Acacb** | -0.64271 | 1.84E-02 |
| **Bicd2** | 0.50308 | 1.72E-04 | **Rpl35a** | -0.64453 | 9.82E-04 |
| **Mfhas1** | 0.50034 | 2.41E-02 | **Rpl26** | -0.64517 | 1.83E-03 |
| **Neurl3** | 0.499903 | 2.56E-03 | **Rpl17** | -0.64543 | 2.76E-03 |
| **A530017D24Rik** | 0.499225 | 2.86E-02 | **Atp5k** | -0.64628 | 6.74E-05 |
| **Ogt** | 0.498547 | 4.63E-02 | **Rps25** | -0.64755 | 2.90E-03 |
| **Snhg17** | 0.498361 | 2.49E-02 | **Alox5** | -0.64836 | 1.42E-02 |
| **Pitpnm1** | 0.49796 | 7.16E-04 | **Rpl38** | -0.64839 | 1.17E-03 |
| **Lmntd2** | 0.496021 | 2.98E-02 | **Rpl27a** | -0.64948 | 1.68E-03 |
| **Egfr** | 0.493671 | 3.65E-03 | **Gm5963** | -0.64982 | 2.39E-02 |
| **Pgghg** | 0.493363 | 1.90E-04 | **Tpt1** | -0.65626 | 2.73E-07 |
| **Ttll3** | 0.491687 | 4.53E-02 | **Rhobtb1** | -0.65695 | 2.42E-02 |
| **Ppfibp1** | 0.491432 | 5.53E-03 | **Pla2g10** | -0.65695 | 6.70E-03 |
| **Krit1** | 0.491408 | 6.49E-03 | **Gm12254** | -0.65696 | 7.07E-03 |
| **Mov10** | 0.489136 | 2.32E-02 | **Ccdc28b** | -0.6579 | 2.60E-02 |
| **9230112E08Rik** | 0.488098 | 3.60E-02 | **Gm9616** | -0.66098 | 3.78E-04 |
| **Samd8** | 0.483111 | 3.13E-02 | **Gpt2** | -0.66361 | 9.38E-03 |
| **Mycl** | 0.481806 | 2.58E-02 | **Gm14586** | -0.66427 | 2.90E-02 |
| **Mapk8ip3** | 0.479887 | 2.29E-03 | **Rnf130** | -0.66507 | 4.71E-03 |
| **Eme2** | 0.478224 | 1.34E-02 | **Rpl31** | -0.66633 | 7.28E-04 |
| **Apbb3** | 0.478187 | 2.86E-02 | **Pecr** | -0.66687 | 1.51E-02 |
| **Pomgnt1** | 0.477802 | 4.14E-04 | **Cdkn2c** | -0.66813 | 8.07E-03 |
| **Stat2** | 0.476999 | 1.12E-02 | **Rps19** | -0.67362 | 7.71E-03 |
| **Nfic** | 0.475784 | 4.08E-02 | **Fmc1** | -0.67588 | 2.99E-04 |
| **Mdm4** | 0.474861 | 3.08E-02 | **Rps26-ps1** | -0.67635 | 1.37E-02 |
| **Tnfsf10** | 0.474552 | 2.20E-02 | **Bmyc** | -0.67871 | 2.87E-02 |
| **Rbm12** | 0.472472 | 3.98E-02 | **Tpsg1** | -0.68061 | 1.03E-04 |
| **Far1** | 0.470092 | 2.53E-02 | **Rpl35** | -0.68718 | 7.10E-03 |
| **Nfat5** | 0.469531 | 2.82E-02 | **Mettl7b** | -0.68744 | 5.97E-05 |
| **Ipo4** | 0.46888 | 6.27E-03 | **Alkbh7** | -0.68841 | 2.03E-03 |
| **Atat1** | 0.467742 | 4.45E-02 | **Khdrbs3** | -0.68877 | 4.84E-02 |
| **Oplah** | 0.466747 | 7.87E-04 | **Agr2** | -0.69507 | 9.82E-04 |
| **Ttc14** | 0.465859 | 1.33E-02 | **Smim26** | -0.69634 | 1.30E-02 |
| **Xpnpep3** | 0.465377 | 2.82E-02 | **Snhg18** | -0.69997 | 7.33E-05 |
| **Rnf135** | 0.464911 | 4.82E-02 | **Rpl32** | -0.7035 | 5.74E-03 |
| **Orai2** | 0.463424 | 4.75E-02 | **Snx30** | -0.70681 | 1.57E-02 |
| **Xpo1** | 0.460869 | 3.76E-03 | **Rps27** | -0.70804 | 1.17E-05 |
| **Atrn** | 0.460194 | 8.90E-03 | **Ston1** | -0.70836 | 7.89E-03 |
| **Rab15** | 0.459919 | 2.83E-02 | **Adra2a** | -0.71344 | 4.26E-02 |
| **Entpd2** | 0.459903 | 1.90E-02 | **Hsd3b3** | -0.71479 | 6.36E-03 |
| **Usp20** | 0.456258 | 6.73E-04 | **Atp5e** | -0.71594 | 2.54E-05 |
| **Nectin1** | 0.456225 | 2.84E-04 | **Gm43672** | -0.71651 | 2.14E-02 |
| **Flot2** | 0.453699 | 1.66E-02 | **Ndrg2** | -0.71744 | 2.37E-03 |
| **Plbd2** | 0.451557 | 9.09E-06 | **Gm15500** | -0.72329 | 3.72E-02 |
| **Slc39a14** | 0.450129 | 3.02E-02 | **Tpbg** | -0.7379 | 1.96E-02 |
| **Lif** | 0.448377 | 4.83E-02 | **Rpl21-ps8** | -0.74673 | 4.90E-03 |
| **Znrf1** | 0.444932 | 1.28E-02 | **Ncald** | -0.74701 | 2.84E-04 |
| **Tnfrsf11a** | 0.444905 | 1.68E-03 | **Gm11478** | -0.74761 | 3.57E-02 |
| **Pea15a** | 0.443215 | 1.42E-02 | **Ramp1** | -0.75062 | 3.03E-04 |
| **Rhou** | 0.442561 | 4.63E-02 | **Rpl41** | -0.75116 | 7.43E-04 |
| **Zdhhc18** | 0.441834 | 2.09E-03 | **Sh3rf2** | -0.75571 | 3.33E-03 |
| **Nbeal2** | 0.441074 | 2.79E-03 | **Spa17** | -0.75888 | 4.48E-02 |
| **Gm44950** | 0.439756 | 3.98E-02 | **Tcea1-ps1** | -0.76163 | 1.98E-02 |
| **Zkscan3** | 0.43967 | 3.39E-02 | **Naip1** | -0.76752 | 2.13E-04 |
| **Gramd2** | 0.438991 | 1.33E-02 | **Pllp** | -0.76795 | 2.17E-04 |
| **Nsmf** | 0.435927 | 8.90E-03 | **Cyp2j6** | -0.77319 | 1.35E-02 |
| **Kdm1b** | 0.434368 | 7.34E-04 | **Gm2962** | -0.77507 | 7.20E-04 |
| **Morc3** | 0.434283 | 2.19E-05 | **Zfp503** | -0.77561 | 4.98E-02 |
| **Sema4g** | 0.433357 | 2.07E-02 | **Creb3l1** | -0.77575 | 8.70E-04 |
| **Stk39** | 0.433249 | 1.67E-02 | **Nxf7** | -0.78181 | 1.62E-02 |
| **Nectin2** | 0.432557 | 3.28E-04 | **Rapgef3** | -0.78555 | 2.14E-02 |
| **Uba7** | 0.431406 | 1.84E-02 | **Asrgl1** | -0.81386 | 2.30E-02 |
| **Serinc5** | 0.430349 | 3.84E-02 | **Rpsa-ps10** | -0.81508 | 6.01E-03 |
| **Zbtb18** | 0.428516 | 6.32E-05 | **Gm5485** | -0.81621 | 5.75E-03 |
| **Ddr1** | 0.427923 | 5.57E-03 | **Kyat3** | -0.81752 | 2.65E-03 |
| **Dmtf1** | 0.423689 | 6.36E-03 | **Tmem252** | -0.82045 | 9.35E-05 |
| **Tcirg1** | 0.422315 | 5.46E-03 | **Tff3** | -0.82052 | 5.84E-04 |
| **Srsf11** | 0.420972 | 3.34E-02 | **Pde8b** | -0.82289 | 9.93E-03 |
| **H2-Q4** | 0.419991 | 4.24E-02 | **Spink4** | -0.82514 | 8.88E-03 |
| **Acot11** | 0.418542 | 3.44E-02 | **Steap3** | -0.83577 | 4.41E-03 |
| **Rnf31** | 0.416943 | 2.18E-02 | **Acat1** | -0.83939 | 2.33E-04 |
| **Recql5** | 0.415193 | 2.58E-03 | **Kcnh3** | -0.84593 | 1.62E-02 |
| **Gga3** | 0.414049 | 2.69E-03 | **Kdelr3** | -0.85119 | 1.76E-03 |
| **Elovl7** | 0.412588 | 1.84E-02 | **Gng11** | -0.85456 | 1.51E-02 |
| **Cnnm3** | 0.410722 | 2.79E-02 | **AI427809** | -0.85852 | 3.09E-03 |
| **Parp14** | 0.410313 | 4.37E-02 | **Gm10221** | -0.8763 | 1.30E-06 |
| **Tasor** | 0.403844 | 2.93E-02 | **Clic6** | -0.87792 | 2.58E-02 |
| **Srgap2** | 0.402735 | 2.32E-02 | **Itpr1** | -0.88633 | 9.93E-03 |
| **Abca7** | 0.401484 | 5.27E-03 | **Acvr1c** | -0.89079 | 3.20E-03 |
| **Slc27a1** | 0.398802 | 5.50E-03 | **Zg16** | -0.89859 | 9.15E-05 |
| **Rexo1** | 0.398739 | 7.28E-05 | **Rps18-ps6** | -0.90936 | 6.00E-03 |
| **Dcun1d5** | 0.396206 | 1.85E-02 | **Chchd2-ps** | -0.93137 | 3.98E-02 |
| **Cdc73** | 0.395773 | 5.84E-04 | **Cox7a1** | -0.93354 | 4.76E-04 |
| **Map4k2** | 0.394686 | 4.91E-02 | **Cuedc1** | -0.94087 | 5.16E-03 |
| **Fbxo11** | 0.394436 | 5.54E-03 | **Rps15a-ps5** | -0.94695 | 3.35E-02 |
| **3830406C13Rik** | 0.393281 | 4.24E-02 | **Nupr1** | -0.94931 | 6.28E-03 |
| **Mafg** | 0.391772 | 3.27E-02 | **Syna** | -0.95628 | 2.40E-02 |
| **Tpcn2** | 0.390347 | 3.28E-02 | **Macrod2** | -0.9573 | 9.09E-04 |
| **Cftr** | 0.389398 | 2.76E-03 | **Selenbp2** | -0.95807 | 7.07E-03 |
| **Dock6** | 0.387234 | 4.58E-02 | **Il18** | -0.96073 | 2.86E-02 |
| **Shisa5** | 0.381429 | 6.05E-03 | **Sst** | -0.97135 | 3.75E-03 |
| **Tcim** | 0.380573 | 2.12E-02 | **Epb41l4a** | -0.97164 | 9.28E-03 |
| **Lamb3** | 0.380556 | 1.81E-02 | **Gm13657** | -0.98104 | 4.04E-02 |
| **Szt2** | 0.379662 | 3.24E-03 | **2310022B05Rik** | -0.99591 | <1e-10 |
| **Rnf139** | 0.37835 | 3.54E-02 | **Prelp** | -0.99941 | 1.79E-02 |
| **Adar** | 0.377186 | 3.85E-03 | **Kcnu1** | -1.00153 | 4.67E-02 |
| **Sufu** | 0.370886 | 1.25E-02 | **Efna3** | -1.003 | 1.46E-02 |
| **Tada1** | 0.365836 | 1.77E-03 | **Trpa1** | -1.01351 | 3.62E-02 |
| **Tmem165** | 0.36569 | 3.07E-03 | **3300005D01Rik** | -1.0229 | 2.69E-02 |
| **Fbxo33** | 0.362982 | 8.43E-03 | **Peli2** | -1.02846 | 5.16E-04 |
| **Tapbp** | 0.360223 | 1.09E-02 | **Chrm3** | -1.03239 | 2.93E-02 |
| **Rai14** | 0.359605 | 1.36E-02 | **Fmo1** | -1.0339 | 6.39E-03 |
| **Vsig10** | 0.358813 | 2.59E-02 | **Bambi** | -1.03433 | 1.29E-02 |
| **Hps5** | 0.358761 | 7.28E-03 | **Otud1** | -1.04247 | 1.15E-02 |
| **Tmc4** | 0.358145 | 3.35E-03 | **Upb1** | -1.04248 | 1.23E-02 |
| **Mid2** | 0.354565 | 9.92E-03 | **P2ry4** | -1.04997 | 1.81E-02 |
| **Mgat5** | 0.354495 | 2.23E-03 | **Fam189a1** | -1.05167 | 5.57E-03 |
| **Spns1** | 0.352382 | 1.33E-02 | **Mxra7** | -1.06153 | 2.98E-03 |
| **Zcchc2** | 0.351399 | 3.24E-02 | **Kctd14** | -1.06513 | 2.98E-05 |
| **Stx16** | 0.350217 | 2.39E-02 | **Sybu** | -1.07228 | 5.09E-03 |
| **Tmc6** | 0.350126 | 2.06E-02 | **Rps4l** | -1.08 | 2.63E-04 |
| **Ccnt2** | 0.349246 | 3.67E-02 | **Ywhaq-ps3** | -1.08463 | 3.34E-02 |
| **Vps13a** | 0.349218 | 3.84E-02 | **AI463229** | -1.09007 | 3.30E-02 |
| **Slk** | 0.348775 | 1.55E-02 | **Ttyh1** | -1.09735 | 1.72E-07 |
| **Zfp639** | 0.348615 | 4.47E-02 | **Hpd** | -1.10266 | 1.56E-04 |
| **Phf20l1** | 0.347108 | 3.44E-02 | **Habp2** | -1.10736 | 3.19E-02 |
| **Khdc4** | 0.345963 | 4.75E-02 | **Armh4** | -1.11171 | 3.19E-02 |
| **Dnajc16** | 0.342853 | 8.81E-03 | **Klk1** | -1.11545 | 3.51E-08 |
| **Sirt6** | 0.341891 | 4.74E-03 | **Ces2a** | -1.12055 | 1.77E-02 |
| **Pdcd11** | 0.341447 | 4.41E-02 | **Gm14303** | -1.12838 | 3.79E-03 |
| **Psip1** | 0.340729 | 3.66E-02 | **Tmem44** | -1.13641 | 6.28E-03 |
| **Hnrnph1** | 0.340652 | 3.00E-02 | **2010007H06Rik** | -1.14002 | 4.63E-03 |
| **Zmym6** | 0.338896 | 2.50E-02 | **Tst** | -1.14876 | 8.61E-08 |
| **Ano9** | 0.338611 | 1.48E-02 | **Mgll** | -1.14936 | 1.28E-03 |
| **Slc38a2** | 0.338442 | 7.67E-03 | **Fhit** | -1.15668 | 8.90E-03 |
| **Parp10** | 0.337041 | 2.97E-02 | **Prr5l** | -1.15836 | 4.77E-03 |
| **Ndufaf4** | 0.335878 | 4.32E-02 | **Sytl5** | -1.16466 | 3.28E-02 |
| **Gm49336** | 0.333299 | 1.03E-02 | **Gm6969** | -1.17576 | 9.81E-06 |
| **Pign** | 0.332624 | 1.38E-02 | **Zmat4** | -1.18063 | 2.57E-02 |
| **Relb** | 0.332502 | 3.00E-02 | **2010001M07Rik** | -1.18168 | 6.49E-03 |
| **Smpd2** | 0.331763 | 9.53E-03 | **Frmd3** | -1.1857 | 9.53E-03 |
| **Traf2** | 0.330459 | 4.53E-03 | **Cldn8** | -1.19292 | 2.11E-02 |
| **Ddx17** | 0.32943 | 3.91E-02 | **Slc2a13** | -1.19869 | 1.77E-03 |
| **Map11** | 0.328487 | 4.66E-02 | **Syne3** | -1.19945 | 9.72E-04 |
| **Uxs1** | 0.32824 | 3.82E-02 | **Cyp2c55** | -1.21651 | 1.12E-02 |
| **Adam10** | 0.326911 | 3.33E-02 | **Pnmal2** | -1.21704 | 7.66E-04 |
| **Cyb561a3** | 0.325819 | 1.74E-02 | **Zfp9** | -1.2191 | 2.43E-03 |
| **Ndst2** | 0.324228 | 3.15E-02 | **Klhdc7a** | -1.233 | 1.15E-02 |
| **Cars2** | 0.322166 | 3.34E-02 | **Ltc4s** | -1.23555 | 4.76E-04 |
| **Txndc11** | 0.321462 | 2.96E-02 | **2610528A11Rik** | -1.23655 | 2.35E-04 |
| **Zfp445** | 0.321245 | 1.51E-02 | **Gm44386** | -1.24804 | 1.84E-02 |
| **Plekhm2** | 0.319516 | 4.28E-02 | **Slc1a3** | -1.25377 | 1.50E-03 |
| **Atp2c1** | 0.318257 | 6.72E-03 | **Samd12** | -1.27925 | 4.66E-02 |
| **Colgalt1** | 0.317551 | 4.76E-04 | **Prss12** | -1.30392 | 3.83E-02 |
| **Ints8** | 0.317101 | 3.63E-02 | **Fsd1** | -1.32263 | 3.83E-02 |
| **Adcy6** | 0.317011 | 3.03E-02 | **Gm28875** | -1.32931 | 3.59E-03 |
| **Tmem82** | 0.31625 | 3.29E-03 | **Rnf150** | -1.34413 | 7.19E-04 |
| **Usp12** | 0.31297 | 2.53E-02 | **Acsm3** | -1.36887 | 2.19E-07 |
| **Med23** | 0.312227 | 1.44E-03 | **Rps23rg1** | -1.36926 | 1.65E-02 |
| **Sdc1** | 0.308455 | 2.56E-02 | **Slc1a2** | -1.37466 | 2.12E-02 |
| **Fbxw5** | 0.305263 | 8.18E-03 | **Pex5l** | -1.39236 | 6.57E-03 |
| **Spats2** | 0.298718 | 3.91E-02 | **Pdia2** | -1.39437 | 4.63E-02 |
| **Birc2** | 0.295809 | 2.79E-02 | **Gm16863** | -1.40901 | 1.93E-02 |
| **Cbfb** | 0.292644 | 2.48E-02 | **Gm15200** | -1.41316 | 1.75E-02 |
| **Pcnx3** | 0.286392 | 1.15E-02 | **Defa3** | -1.42868 | 2.87E-02 |
| **Armt1** | 0.284311 | 4.88E-02 | **Rbp7** | -1.43032 | 5.27E-04 |
| **Otud4** | 0.282475 | 7.78E-03 | **Defa5** | -1.44013 | 2.48E-02 |
| **Zfp598** | 0.281787 | 3.34E-03 | **Nrn1** | -1.44581 | 3.72E-03 |
| **Tug1** | 0.278093 | 2.73E-02 | **Apol10c-ps** | -1.45927 | 3.13E-02 |
| **Sart3** | 0.26759 | 3.42E-02 | **Ccbe1** | -1.47637 | 6.28E-03 |
| **Rbm26** | 0.267538 | 2.58E-02 | **Fras1** | -1.48563 | 6.75E-03 |
| **Spata2** | 0.266611 | 4.62E-02 | **Gm5159** | -1.48919 | 4.17E-04 |
| **Cd2ap** | 0.26505 | 4.16E-02 | **Insrr** | -1.50202 | 8.89E-04 |
| **Phf12** | 0.264254 | 1.70E-02 | **Sypl2** | -1.5075 | 3.92E-06 |
| **Mink1** | 0.263531 | 6.77E-03 | **Lect2** | -1.53889 | 4.82E-02 |
| **Rtn4** | 0.262204 | 4.24E-02 | **Sh3bgr** | -1.53984 | 3.50E-02 |
| **Ncln** | 0.260656 | 1.03E-02 | **Gm32742** | -1.55031 | 9.70E-06 |
| **R3hdm4** | 0.260159 | 1.30E-02 | **Hao2** | -1.55057 | 1.46E-03 |
| **Araf** | 0.252689 | 4.01E-02 | **Defa17** | -1.5906 | 1.97E-02 |
| **Ubp1** | 0.252436 | 2.82E-02 | **Clps** | -1.59839 | 1.38E-05 |
| **Polrmt** | 0.247684 | 4.88E-02 | **Sstr1** | -1.60496 | 6.72E-03 |
| **Senp3** | 0.237545 | 3.60E-02 | **Ccl24** | -1.60835 | 3.03E-03 |
| **U2surp** | 0.227105 | 1.90E-02 | **Defa-ps6** | -1.6339 | 2.91E-02 |
| **Dock7** | 0.22411 | 3.79E-02 | **Gsdmc2** | -1.64943 | 3.93E-02 |
| **Dnm1l** | 0.221835 | 4.75E-02 | **Defa29** | -1.67444 | 8.79E-04 |
| **Unc93b1** | 0.218734 | 4.93E-02 | **Lyz1** | -1.68569 | 2.61E-02 |
| **Prrc2a** | 0.217955 | 4.78E-02 | **Dpysl5** | -1.70274 | 3.04E-02 |
| **Atp13a1** | 0.217015 | 4.71E-03 | **Tdrd1** | -1.72416 | 5.11E-03 |
| **Serinc2** | 0.214855 | 4.95E-02 | **Bglap3** | -1.73835 | 1.33E-03 |
| **Oga** | 0.199255 | 4.47E-02 | **Gm27162** | -1.74343 | 6.39E-03 |
| **Scrib** | 0.198577 | 4.66E-02 | **Afp** | -1.76958 | 2.91E-06 |
| **H13** | 0.195992 | 4.83E-02 | **Defa38** | -1.79091 | 3.71E-02 |
| **Ncstn** | 0.192232 | 4.76E-02 | **Gnat3** | -1.88482 | 2.17E-04 |
| **Ranbp3** | 0.17617 | 3.72E-02 | **Asb16** | -1.8906 | 8.66E-03 |
| **Scaf1** | 0.171151 | 1.62E-02 | **Cabp1** | -1.91194 | 4.28E-02 |
| **Camsap3** | -0.14981 | 4.33E-02 | **Gm14403** | -1.95611 | 7.71E-03 |
| **Qars** | -0.17409 | 2.18E-02 | **Erdr1** | -1.97276 | 7.71E-03 |
| **Tmem234** | -0.17895 | 4.64E-02 | **Ugt1a9** | -1.98856 | 1.74E-02 |
| **Mindy1** | -0.20436 | 1.03E-02 | **Defa40** | -2.01334 | 1.18E-03 |
| **Tmco1** | -0.21244 | 2.30E-02 | **Gcg** | -2.06874 | 3.34E-10 |
| **Dazap2** | -0.21393 | 3.25E-02 | **Grip2** | -2.09204 | 2.90E-03 |
| **Skp1a** | -0.21565 | 2.60E-02 | **Wfikkn2** | -2.19229 | 5.45E-03 |
| **Arpc5** | -0.21682 | 2.97E-02 | **Gm830** | -2.21104 | 4.01E-07 |
| **Atg13** | -0.22266 | 4.29E-02 | **Gsdmc4** | -2.28123 | 3.78E-04 |
| **Tpd52** | -0.22617 | 4.32E-02 | **Defa26** | -2.29505 | 9.78E-04 |
| **Rab40c** | -0.24042 | 3.92E-02 | **Nts** | -2.37754 | <1e-10 |
| **Pcbp2** | -0.24367 | 2.46E-02 | **Cyp2f2** | -2.39182 | 3.97E-02 |
| **Rap1gap2** | -0.24601 | 4.02E-02 | **Gm15408** | -2.43501 | 1.66E-02 |
| **Idh3b** | -0.24752 | 4.41E-02 | **Defa-ps7** | -2.43772 | 6.08E-05 |
| **Mrpl10** | -0.24786 | 1.50E-02 | **Tat** | -2.58818 | 1.33E-03 |
| **Morf4l1** | -0.24827 | 1.86E-02 | **H2-Ob** | -2.68491 | 4.28E-02 |
| **Mapk3** | -0.24939 | 2.17E-02 | **H2-Eb2** | -2.71822 | 1.09E-02 |
| **Mkrn1** | -0.25289 | 1.98E-02 | **Kcns3** | -2.72671 | 3.48E-05 |
| **Elp5** | -0.25387 | 3.46E-02 | **Shank1** | -2.73833 | 4.26E-02 |
| **Mrfap1** | -0.25711 | 2.51E-02 | **Ighg2b** | -2.85187 | 2.29E-02 |
| **Gpr108** | -0.25754 | 1.84E-02 | **Ms4a1** | -3.26691 | 2.66E-02 |
| **Tmed2** | -0.25769 | 1.90E-02 | **Npy1r** | -3.48924 | 2.43E-02 |
| **Psmc3** | -0.26121 | 4.03E-02 | **Cr2** | -3.92154 | 4.13E-02 |
| **Cox19** | -0.26194 | 3.37E-02 | **Bank1** | -4.60917 | 4.40E-02 |
| **Ppp1cc** | -0.26203 | 2.52E-02 | **Flrt2** | -4.76748 | 3.95E-03 |
| **Akr1a1** | -0.26373 | 4.59E-02 | **Ddx3y** | -10.5035 | 3.03E-02 |
| **Rtcb** | -0.26415 | 3.13E-02 | **Kdm5d** | -11.0008 | 6.52E-03 |
| **Pmpcb** | -0.2642 | 3.72E-02 | **Uty** | -24.9464 | <1e-10 |
| **Cops4** | -0.26477 | 3.62E-02 | **Eif2s3y** | -26.5049 | <1e-10 |
| **Spop** | -0.26488 | 1.59E-02 |  |  |  |
