# Supplementary material for: CARD14 signaling in intestinal epithelial cells induces intestinal inflammation and intestinal transit delay
Source: EMBO Mol Med. 2025 Oct 23;17(12):3300–28. doi: 10.1038/s44321-025-00321-4 (PMC12686530; doi:10.1038/s44321-025-00321-4)
Supplement: Supplementary file 2 — Table EV2 [file 44321_2025_321_MOESM2_ESM.docx]

**Table EV2: Genes that are differentially expressed between WT and *CARD14*(E138A)^IEC^ mice in IECs of the colon**

A total of 1,627 genes were differentially expressed (FDR < 0.05). Among these, 290 genes were upregulated and 202 genes were downregulated with at least a two-fold change (|log_2_FC| ≥ 1). Notably, 62 genes were upregulated and 44 genes were downregulated with at least a five-fold change (|log_2_FC| ≥ 2.32). Log_2_FC, Log fold change; FDR, False discovery rate.

| Gene | LogFC | FDR | Gene | LogFC | FDR |
| --- | --- | --- | --- | --- | --- |
| Defb1 | 4.549744 | 4.56E-04 | **Tbc1d22b** | -0.2675 | 3.01E-02 |
| Pdyn | 4.371165 | 3.06E-09 | **Galnt4** | -0.26877 | 3.90E-03 |
| Sema3e | 3.954843 | <1e-10 | **Copa** | -0.27098 | 2.80E-02 |
| Pigz | 3.901815 | 8.66E-06 | **Cnot6l** | -0.27199 | 4.09E-02 |
| Cxcl2 | 3.777268 | 1.44E-03 | **Acss2** | -0.27266 | 4.17E-02 |
| Ubd | 3.715574 | 1.13E-09 | **Pgm3** | -0.27297 | 3.94E-02 |
| Slitrk6 | 3.489387 | 2.82E-02 | **Slc35a1** | -0.27606 | 1.87E-02 |
| Ankfn1 | 3.473407 | 5.61E-04 | **Tab2** | -0.27749 | 3.67E-02 |
| Nos2 | 3.439758 | 1.38E-04 | **Pura** | -0.27807 | 3.96E-02 |
| C3 | 3.436929 | 2.80E-05 | **Coq9** | -0.27886 | 4.21E-02 |
| Ighv3-6 | 3.383488 | 2.64E-02 | **Fpgt** | -0.27974 | 4.52E-02 |
| Tff2 | 3.297206 | 1.61E-02 | **Lims1** | -0.27988 | 1.43E-02 |
| Dlk1 | 3.279311 | 8.32E-06 | **Galk2** | -0.28198 | 1.79E-02 |
| Nr1h5 | 3.177775 | 5.54E-03 | **Abhd13** | -0.28204 | 3.11E-02 |
| Col17a1 | 3.168109 | 1.04E-03 | **Mtdh** | -0.28275 | 2.55E-03 |
| Tnf | 3.163797 | <1e-10 | **Ctsc** | -0.28309 | 4.26E-02 |
| Megf6 | 3.093289 | 1.06E-05 | **Gdi2** | -0.28348 | 1.73E-02 |
| Tmem213 | 3.087279 | 4.11E-04 | **Yipf3** | -0.28349 | 7.48E-03 |
| Chit1 | 3.045617 | 5.71E-05 | **Arhgef12** | -0.28376 | 3.98E-02 |
| Ncf4 | 2.950592 | 2.21E-09 | **Crkl** | -0.28412 | 3.03E-02 |
| Vcam1 | 2.933813 | 3.23E-09 | **Sucla2** | -0.28432 | 3.08E-02 |
| Igfals | 2.929023 | 1.03E-10 | **Cdc42ep4** | -0.28475 | 4.89E-02 |
| Ky | 2.915804 | <1e-10 | **Osbpl10** | -0.28624 | 1.68E-02 |
| Pxdn | 2.87289 | 6.34E-08 | **Sar1a** | -0.28743 | 1.01E-03 |
| Tspan32 | 2.867898 | 3.18E-02 | **Hepacam2** | -0.28761 | 1.20E-02 |
| Rarb | 2.843625 | 1.51E-08 | **Map3k5** | -0.28777 | 2.96E-02 |
| 4732419C18Rik | 2.748391 | 4.90E-03 | **Pdia3** | -0.28958 | 1.29E-02 |
| Fam89a | 2.738748 | 2.27E-02 | **Rab18** | -0.29007 | 3.30E-02 |
| Cxcl5 | 2.725626 | 1.03E-03 | **Copb1** | -0.29226 | 3.98E-02 |
| Ccl20 | 2.72201 | 1.62E-10 | **Rdh11** | -0.29307 | 4.91E-02 |
| Chst8 | 2.657916 | <1e-10 | **Golga5** | -0.294 | 4.99E-03 |
| Ighv10-1 | 2.657038 | 1.64E-02 | **Foxp1** | -0.2942 | 2.65E-02 |
| Tnfrsf8 | 2.646624 | 1.56E-08 | **Gars** | -0.29529 | 1.63E-02 |
| Igkv3-2 | 2.622891 | 1.68E-02 | **Copb2** | -0.29557 | 3.98E-02 |
| 2410018L13Rik | 2.611708 | 1.02E-03 | **Atp2a3** | -0.29619 | 3.35E-02 |
| Mmp7 | 2.611167 | 6.27E-08 | **Polr3b** | -0.29648 | 1.68E-02 |
| Pirt | 2.549305 | 6.86E-06 | **Mgat4b** | -0.29905 | 8.71E-03 |
| Il1b | 2.542885 | 3.19E-02 | **Klhl21** | -0.30066 | 3.74E-02 |
| Gm29684 | 2.515113 | 9.79E-04 | **Rmnd5a** | -0.30077 | 3.10E-02 |
| Npnt | 2.500869 | 1.88E-04 | **Ilk** | -0.30094 | 9.18E-04 |
| Gabbr2 | 2.483291 | 5.43E-04 | **Eif3l** | -0.30124 | 2.89E-02 |
| Col4a1 | 2.458593 | 2.89E-02 | **Eif2a** | -0.3022 | 8.11E-03 |
| Rasal1 | 2.453751 | <1e-10 | **Opa1** | -0.30252 | 1.27E-02 |
| Sirpa | 2.446817 | <1e-10 | **Edem1** | -0.30288 | 1.05E-02 |
| Icam1 | 2.442536 | <1e-10 | **Ap3m1** | -0.30405 | 1.97E-02 |
| Frmd5 | 2.433502 | 1.90E-09 | **Ap3b1** | -0.30422 | 4.17E-02 |
| Stx11 | 2.431014 | <1e-10 | **Arfip2** | -0.30435 | 3.39E-02 |
| Cxcl1 | 2.414447 | 1.35E-03 | **Tcf12** | -0.30457 | 1.67E-02 |
| Gabrp | 2.413894 | 7.40E-05 | **Isca1** | -0.30481 | 2.12E-02 |
| Flt3l | 2.41113 | <1e-10 | **Uqcc1** | -0.30491 | 4.02E-02 |
| Fgf15 | 2.407414 | 1.68E-02 | **Slc25a32** | -0.30502 | 3.82E-02 |
| Zmynd15 | 2.403745 | <1e-10 | **Dhrs7** | -0.30515 | 3.54E-02 |
| Atp6v0a4 | 2.382969 | 2.84E-09 | **Rap2a** | -0.30599 | 4.14E-02 |
| 1700016C15Rik | 2.372459 | 7.81E-04 | **Ssr3** | -0.308 | 3.29E-02 |
| Cldn14 | 2.358518 | 1.36E-10 | **Zfp652** | -0.30839 | 4.58E-04 |
| Gm31219 | 2.357158 | 1.35E-02 | **Slc35c1** | -0.30937 | 1.76E-02 |
| Gm17182 | 2.350511 | 3.00E-02 | **Atg13** | -0.30962 | 1.52E-03 |
| Podnl1 | 2.345313 | 3.88E-02 | **Setd5** | -0.30992 | 1.21E-04 |
| Guca2b | 2.337915 | <1e-10 | **Canx** | -0.30993 | 2.67E-02 |
| Msln | 2.323763 | 1.66E-05 | **Atp5b** | -0.311 | 2.67E-02 |
| 9130204K15Rik | 2.323156 | 1.47E-02 | **Snd1** | -0.31192 | 2.75E-02 |
| Kcnip3 | 2.320624 | 1.02E-03 | **Smim13** | -0.3127 | 4.73E-02 |
| Gpr37l1 | 2.311104 | 3.74E-02 | **Nr3c1** | -0.31548 | 2.18E-02 |
| Gm6637 | 2.30288 | 1.56E-02 | **Klf9** | -0.31588 | 8.11E-03 |
| Adamts7 | 2.290246 | 1.29E-05 | **Pabpc1** | -0.31591 | 4.85E-02 |
| Bend4 | 2.282651 | 3.93E-03 | **Tmem170b** | -0.31608 | 3.81E-02 |
| Sec14l4 | 2.276808 | 1.16E-04 | **Tm9sf1** | -0.31755 | 9.47E-03 |
| Megf11 | 2.276388 | 1.64E-02 | **Ostc** | -0.31828 | 2.46E-02 |
| Kl | 2.269368 | 6.87E-03 | **Eif3k** | -0.31881 | 8.78E-03 |
| Dusp13 | 2.253146 | 1.16E-04 | **Mcoln2** | -0.31969 | 1.68E-02 |
| Efs | 2.222731 | 7.76E-05 | **Znrf2** | -0.32077 | 1.33E-02 |
| Gp2 | 2.222409 | 1.77E-02 | **Pcyox1** | -0.32122 | 1.12E-02 |
| Reg3g | 2.182181 | 6.90E-04 | **Arf1** | -0.32189 | 1.83E-02 |
| Plxdc1 | 2.171983 | 4.99E-02 | **Clint1** | -0.32197 | 3.86E-03 |
| Cap2 | 2.165063 | 6.73E-09 | **Slc25a24** | -0.32212 | 8.39E-03 |
| Serpina3g | 2.146485 | 1.67E-04 | **Mrps24** | -0.32258 | 5.69E-03 |
| Parp8 | 2.137648 | 6.02E-05 | **Sec16a** | -0.32292 | 2.09E-02 |
| Grb10 | 2.130234 | 2.32E-05 | **Stard10** | -0.32422 | 4.97E-02 |
| Dmrta1 | 2.117097 | 3.31E-07 | **Mknk2** | -0.32448 | 1.54E-03 |
| Slc43a3 | 2.115803 | 8.36E-07 | **Apbb2** | -0.32493 | 4.26E-02 |
| Tnfrsf9 | 2.099953 | 6.38E-05 | **Cd164** | -0.325 | 4.90E-03 |
| Wnt10b | 2.081612 | 7.01E-03 | **Usf3** | -0.32611 | 2.37E-02 |
| Csdc2 | 2.07793 | 8.18E-03 | **Larp4** | -0.32709 | 2.45E-02 |
| Nox1 | 2.072303 | 1.37E-10 | **Slc39a11** | -0.32892 | 2.04E-02 |
| Slpi | 2.071564 | 7.91E-04 | **Ppp1r2** | -0.32903 | 1.98E-03 |
| Lama4 | 2.069071 | 2.09E-03 | **Lrp5** | -0.32904 | 1.16E-02 |
| Osmr | 2.047178 | 1.19E-02 | **Yif1a** | -0.32931 | 4.06E-02 |
| Ccdc190 | 2.041804 | 3.46E-03 | **Vdac3-ps1** | -0.32934 | 1.35E-02 |
| D630011A20Rik | 2.029058 | 4.19E-03 | **Arid1b** | -0.33004 | 3.14E-02 |
| Cd300c2 | 2.023491 | 3.19E-02 | **Cndp2** | -0.33005 | 1.68E-02 |
| Atp12a | 2.018702 | 3.11E-02 | **Dpep1** | -0.33039 | 3.35E-02 |
| Shank3 | 2.0027 | 3.08E-04 | **Copz1** | -0.33317 | 2.00E-02 |
| Cd33 | 1.986049 | 2.20E-02 | **Stard7** | -0.3342 | 1.13E-02 |
| Gm2420 | 1.976931 | 3.16E-02 | **Sertad2** | -0.3361 | 8.38E-03 |
| Rgl1 | 1.961775 | 6.25E-04 | **Hadha** | -0.3361 | 1.42E-02 |
| Loxl2 | 1.956685 | 3.62E-02 | **Kif16b** | -0.33623 | 1.20E-02 |
| Slc2a6 | 1.942365 | 6.22E-07 | **Sec62** | -0.33645 | 4.07E-03 |
| Adora1 | 1.922083 | 1.86E-10 | **Fcsk** | -0.338 | 9.08E-03 |
| Gpr34 | 1.921055 | 5.24E-03 | **Eif4b** | -0.3391 | 9.08E-03 |
| Gbp8 | 1.908422 | 1.44E-05 | **Kdelr2** | -0.33997 | 1.05E-02 |
| Steap4 | 1.889492 | <1e-10 | **Prdm2** | -0.34037 | 3.43E-02 |
| Nfkbid | 1.87836 | <1e-10 | **Tmf1** | -0.34102 | 1.60E-02 |
| Cyp2d12 | 1.874071 | 9.72E-03 | **Mtmr9** | -0.34259 | 4.15E-02 |
| Syne4 | 1.865287 | 3.87E-08 | **Dnajb11** | -0.34291 | 2.85E-02 |
| Cxcl16 | 1.86463 | <1e-10 | **Arl1** | -0.34327 | 1.27E-02 |
| Slc7a11 | 1.848618 | <1e-10 | **Ndufa10** | -0.34341 | 2.05E-02 |
| Nfkbie | 1.842234 | <1e-10 | **Tomm20** | -0.344 | 4.62E-02 |
| Lst1 | 1.839155 | 7.10E-03 | **Tmtc2** | -0.34504 | 1.25E-02 |
| Csf1 | 1.832808 | 1.07E-04 | **Hdac1** | -0.3454 | 3.08E-02 |
| Gm14137 | 1.824521 | <1e-10 | **Hipk3** | -0.34605 | 1.66E-02 |
| Cytip | 1.819438 | <1e-10 | **Ccng1** | -0.3487 | 1.25E-02 |
| Fam20c | 1.810011 | 7.63E-06 | **Rad21** | -0.34964 | 1.94E-02 |
| Ripply1 | 1.807932 | 5.81E-04 | **Cpt1a** | -0.34974 | 1.04E-03 |
| Epdr1 | 1.807074 | 7.78E-07 | **Pde4d** | -0.34988 | 9.76E-03 |
| Tnip3 | 1.80571 | 4.03E-07 | **Sgsm1** | -0.34992 | 1.49E-02 |
| B430010I23Rik | 1.799999 | 9.30E-03 | **Sugt1** | -0.35098 | 1.10E-02 |
| Zp2 | 1.799633 | 2.69E-03 | **Golph3** | -0.35118 | 1.70E-02 |
| Ltb | 1.786192 | 3.06E-06 | **Dip2a** | -0.35131 | 6.03E-03 |
| Nfkbia | 1.775157 | <1e-10 | **Ppard** | -0.35329 | 1.80E-02 |
| St8sia5 | 1.771314 | 2.08E-02 | **Hgfac** | -0.35375 | 9.99E-03 |
| Adgrg2 | 1.770568 | 1.04E-04 | **Alg5** | -0.35445 | 1.02E-02 |
| Sertad4 | 1.761966 | 3.80E-06 | **Qsox1** | -0.35459 | 2.24E-03 |
| Gm36283 | 1.75782 | 1.97E-02 | **Vps13b** | -0.35469 | 1.42E-02 |
| Cxcl10 | 1.742511 | 5.27E-05 | **Nfix** | -0.35523 | 1.64E-02 |
| Ahnak2 | 1.737655 | 1.64E-02 | **Nf2** | -0.35525 | 3.86E-03 |
| Mapk11 | 1.732942 | <1e-10 | **Pdk3** | -0.3565 | 1.62E-02 |
| Itgb8 | 1.732288 | 1.44E-02 | **Asph** | -0.35695 | 3.35E-02 |
| Spink1 | 1.721215 | 2.45E-07 | **Suclg2** | -0.35811 | 4.69E-03 |
| Mpdz | 1.719974 | 1.03E-04 | **Tbc1d15** | -0.35881 | 3.78E-02 |
| Adcy1 | 1.716958 | 3.02E-04 | **Fau** | -0.36026 | 2.76E-02 |
| Trgv7 | 1.712907 | 6.87E-03 | **Arcn1** | -0.36138 | 2.14E-02 |
| Abcc8 | 1.710263 | 9.70E-10 | **Pld1** | -0.36168 | 3.16E-02 |
| Cntnap1 | 1.700473 | <1e-10 | **Rnf115** | -0.36349 | 3.92E-02 |
| Mpeg1 | 1.697874 | 1.66E-03 | **Prrc1** | -0.36385 | 5.65E-03 |
| Il12rb2 | 1.682875 | 3.57E-03 | **Rab5b** | -0.36396 | 5.97E-03 |
| Pamr1 | 1.662431 | 2.54E-02 | **Fam210a** | -0.36403 | 5.79E-04 |
| Cd86 | 1.649509 | 2.58E-02 | **Prkar2a** | -0.36421 | 1.54E-02 |
| Sync | 1.640051 | 8.05E-06 | **Stard4** | -0.3645 | 1.57E-02 |
| Gzmm | 1.637773 | 1.68E-02 | **Spty2d1** | -0.3652 | 2.17E-03 |
| Ascl2 | 1.634556 | 9.95E-05 | **Specc1l** | -0.36649 | 1.82E-02 |
| Ranbp17 | 1.62784 | 1.95E-04 | **Deptor** | -0.36681 | 6.28E-04 |
| Cd53 | 1.623083 | 1.02E-02 | **Ankrd13a** | -0.36705 | 3.20E-03 |
| Gm12055 | 1.614751 | 3.77E-02 | **Cpd** | -0.36745 | 3.92E-04 |
| Pld2 | 1.600854 | <1e-10 | **Golm1** | -0.36761 | 1.22E-02 |
| Zfp57 | 1.598986 | 3.96E-03 | **Ccs** | -0.36863 | 2.18E-02 |
| Cd200r4 | 1.58823 | 2.70E-02 | **Acly** | -0.36866 | 1.23E-02 |
| Gm19276 | 1.581709 | 5.09E-04 | **Erlec1** | -0.37013 | 9.30E-03 |
| Dnah7b | 1.569164 | 2.22E-02 | **Nbas** | -0.37191 | 4.55E-02 |
| Abcc4 | 1.560404 | <1e-10 | **Selenom** | -0.37242 | 3.42E-02 |
| Gm33091 | 1.550615 | 1.73E-02 | **Mrs2** | -0.37389 | 4.50E-04 |
| Fam129c | 1.549382 | 8.47E-06 | **Fech** | -0.37478 | 1.72E-03 |
| Sh3tc2 | 1.548764 | 1.86E-03 | **Slc37a3** | -0.37585 | 9.55E-04 |
| Cxcr6 | 1.544645 | 2.79E-03 | **Lclat1** | -0.37645 | 4.27E-03 |
| Neu3 | 1.53612 | 1.51E-02 | **Mrpl30** | -0.37746 | 1.14E-02 |
| Clca3a2 | 1.533121 | <1e-10 | **Bri3bp** | -0.37845 | 1.94E-02 |
| Dnah10 | 1.532266 | 6.34E-04 | **Rpl10** | -0.38064 | 2.29E-02 |
| Ppy | 1.528452 | 2.65E-03 | **Cog6** | -0.38095 | 3.37E-03 |
| Gdf15 | 1.522617 | 1.17E-03 | **Chchd3** | -0.38124 | 1.45E-02 |
| Tnnt2 | 1.50825 | 2.92E-03 | **Ndrg3** | -0.38234 | 2.10E-03 |
| Clca3a1 | 1.498819 | 7.97E-09 | **Tmem30b** | -0.38259 | 3.24E-03 |
| Gprc5c | 1.492497 | 7.48E-05 | **Rab8a** | -0.38281 | 4.89E-02 |
| Gem | 1.491842 | 2.76E-03 | **Dynll2** | -0.38348 | 1.02E-03 |
| Gsap | 1.485901 | 3.35E-05 | **Agps** | -0.38424 | 2.72E-03 |
| Gm30042 | 1.482035 | 2.50E-02 | **Cnksr3** | -0.38429 | 4.20E-02 |
| Dubr | 1.478108 | 6.29E-07 | **Rpl37rt** | -0.38458 | 2.37E-02 |
| Plxnd1 | 1.474529 | <1e-10 | **Hopx** | -0.38476 | 2.61E-02 |
| Kcnj11 | 1.469062 | 2.92E-05 | **Rabgap1l** | -0.38499 | 2.04E-02 |
| Pf4 | 1.46634 | 2.50E-02 | **Edem3** | -0.38565 | 1.78E-02 |
| Pglyrp1 | 1.459839 | <1e-10 | **Galnt10** | -0.38591 | 1.18E-02 |
| Tnfsf10 | 1.457671 | <1e-10 | **Cmip** | -0.38611 | 4.16E-02 |
| Cd101 | 1.450273 | 9.32E-03 | **Prkca** | -0.38635 | 4.35E-04 |
| Il27ra | 1.449204 | 2.61E-03 | **Eif2ak2** | -0.38657 | 2.78E-02 |
| Gm11735 | 1.443025 | 1.47E-04 | **Pygb** | -0.38684 | 2.56E-02 |
| Cyba | 1.441733 | <1e-10 | **Cyp4f40** | -0.38747 | 2.73E-03 |
| Il4i1 | 1.438244 | 4.06E-03 | **Gorasp2** | -0.38934 | 8.81E-03 |
| Spns3 | 1.43721 | 3.10E-04 | **Fzd1** | -0.38937 | 4.98E-02 |
| Syngap1 | 1.433731 | 6.33E-08 | **Prkaa2** | -0.3894 | 6.03E-03 |
| Cacna2d1 | 1.429109 | 5.65E-06 | **Isoc1** | -0.38994 | 1.88E-02 |
| Prkar2b | 1.420614 | 1.48E-04 | **Mrpl12** | -0.39101 | 2.69E-02 |
| Eno3 | 1.41153 | 2.04E-03 | **Fut8** | -0.39367 | 2.21E-02 |
| Rims4 | 1.411343 | 4.38E-04 | **Chm** | -0.39393 | 1.49E-02 |
| Ankrd29 | 1.403701 | 8.30E-03 | **Gne** | -0.39517 | 2.22E-02 |
| Zc3h12c | 1.403291 | 1.68E-03 | **Srpr** | -0.39522 | 2.96E-04 |
| Slc16a3 | 1.402787 | 1.07E-04 | **Glud1** | -0.39579 | 1.88E-03 |
| AW551984 | 1.392514 | 1.25E-02 | **Tbc1d8** | -0.39639 | 2.61E-02 |
| Edn1 | 1.387801 | 1.56E-03 | **Rps9** | -0.39662 | 4.76E-02 |
| Pard6g | 1.385116 | 1.09E-03 | **Mansc1** | -0.39711 | 1.93E-02 |
| Ms4a4b | 1.376133 | 2.83E-03 | **Elovl5** | -0.39795 | 1.43E-03 |
| Map3k14 | 1.368085 | <1e-10 | **Tpsg1** | -0.39879 | 2.78E-02 |
| Slc27a2 | 1.365028 | 6.94E-07 | **Slc25a15** | -0.39978 | 4.20E-02 |
| Tnfaip2 | 1.364572 | 4.81E-03 | **Hibch** | -0.39993 | 1.66E-02 |
| Rgs9 | 1.358107 | 2.10E-02 | **Gm10039** | -0.40069 | 4.17E-02 |
| Cxcl11 | 1.357201 | 1.01E-08 | **Bahcc1** | -0.40115 | 4.53E-02 |
| Csf2rb | 1.350863 | 6.30E-03 | **Eif3h** | -0.40434 | 7.01E-03 |
| Sspo | 1.348237 | 9.56E-05 | **Pon3** | -0.40541 | 3.39E-02 |
| Tnfaip3 | 1.347011 | <1e-10 | **Kank1** | -0.40584 | 4.77E-02 |
| Slco3a1 | 1.341984 | 7.33E-05 | **Qdpr** | -0.40766 | 2.28E-02 |
| Rian | 1.340224 | 1.99E-02 | **Mmut** | -0.40796 | 4.46E-04 |
| Cldn4 | 1.329959 | 1.95E-08 | **Rasa4** | -0.40803 | 3.35E-02 |
| Il34 | 1.315767 | 8.10E-04 | **Rpl19** | -0.40965 | 4.17E-02 |
| Foxq1 | 1.311429 | 5.60E-03 | **Pcca** | -0.4117 | 3.16E-02 |
| Azgp1 | 1.309908 | 7.83E-05 | **Adk** | -0.41317 | 4.25E-03 |
| Ackr3 | 1.307008 | 4.76E-02 | **Fmn1** | -0.41332 | 3.52E-02 |
| Loxl3 | 1.304799 | 1.14E-04 | **Abhd17a** | -0.41336 | 1.97E-02 |
| Rnase1 | 1.302604 | 6.23E-03 | **Ybx1** | -0.41492 | 2.65E-03 |
| Gm35028 | 1.296934 | 5.96E-04 | **Ncor1** | -0.41532 | 3.74E-02 |
| Mirg | 1.296614 | 2.06E-02 | **Lmo4** | -0.41816 | 3.79E-02 |
| Galnt18 | 1.292857 | 9.47E-03 | **Mtch2** | -0.41989 | 6.65E-03 |
| Tcp11 | 1.290754 | 5.67E-03 | **Dmbt1** | -0.42006 | 4.62E-03 |
| E130208F15Rik | 1.287635 | 1.84E-02 | **Tmed3** | -0.42037 | 6.01E-03 |
| Ms4a6b | 1.280156 | 7.10E-03 | **Fn3krp** | -0.4208 | 4.93E-02 |
| Slfn2 | 1.277935 | 3.80E-06 | **Glrx** | -0.42109 | 1.20E-02 |
| Slc6a20a | 1.272799 | 4.35E-06 | **Mrps36** | -0.42128 | 1.24E-02 |
| Trpv3 | 1.272363 | 2.01E-02 | **Ell2** | -0.42202 | 4.17E-02 |
| Gm9926 | 1.268699 | 7.51E-07 | **Prkci** | -0.42213 | 3.37E-02 |
| Cck | 1.263035 | 3.07E-03 | **Hsdl2** | -0.42324 | 4.99E-02 |
| Gm47171 | 1.262503 | 3.74E-03 | **Snhg18** | -0.42336 | 1.05E-02 |
| Rasgef1a | 1.255856 | 3.35E-02 | **Hid1** | -0.42393 | 9.47E-03 |
| Ttr | 1.253556 | 4.27E-03 | **Rpl7a** | -0.42502 | 2.54E-02 |
| Lbp | 1.237224 | 2.14E-02 | **Rapgef5** | -0.42607 | 2.51E-03 |
| Trpm6 | 1.237143 | 1.88E-02 | **Rpl22** | -0.42703 | 4.28E-02 |
| Meg3 | 1.236989 | 3.82E-02 | **Sypl** | -0.42863 | 1.25E-03 |
| Fbxo32 | 1.234387 | 1.25E-02 | **Sfxn1** | -0.42873 | 4.69E-03 |
| Trabd2b | 1.229117 | 5.26E-03 | **Rpl11** | -0.42913 | 2.61E-02 |
| Muc1 | 1.227889 | 1.55E-03 | **Nek6** | -0.42927 | 2.01E-02 |
| Cd5 | 1.225347 | 4.11E-02 | **Cobl** | -0.42948 | 2.22E-02 |
| Gbp9 | 1.224695 | 2.36E-03 | **B3glct** | -0.42981 | 1.74E-03 |
| Plekhs1 | 1.224147 | 3.12E-06 | **Aldh4a1** | -0.43165 | 2.87E-02 |
| Slc25a48 | 1.220499 | 3.11E-03 | **Mfsd4a** | -0.43184 | 3.37E-02 |
| Bcam | 1.213403 | 8.98E-08 | **Rps17** | -0.43208 | 4.00E-02 |
| Tnfrsf19 | 1.209272 | 5.69E-03 | **Gorasp1** | -0.43301 | 3.86E-03 |
| Il7r | 1.2059 | 4.85E-02 | **Eif3f** | -0.43441 | 8.71E-03 |
| Casp4 | 1.197997 | <1e-10 | **Gm18541** | -0.43447 | 4.78E-02 |
| Clca3b | 1.194564 | 4.83E-05 | **Galnt3** | -0.43518 | 8.03E-03 |
| Gm20539 | 1.19059 | 4.31E-02 | **Rps15** | -0.43568 | 3.98E-02 |
| Gm15635 | 1.179932 | 4.71E-03 | **Lrrc8d** | -0.43705 | 9.72E-03 |
| Rhov | 1.173237 | 6.83E-04 | **L2hgdh** | -0.43722 | 4.31E-02 |
| Trgv2 | 1.170815 | 3.29E-02 | **Cux1** | -0.43754 | 4.35E-04 |
| AW112010 | 1.16926 | <1e-10 | **Lpcat2** | -0.43865 | 3.98E-02 |
| 5730507C01Rik | 1.160686 | 1.97E-02 | **Sec24a** | -0.4388 | 4.41E-03 |
| Itgax | 1.157556 | 3.77E-03 | **Slc50a1** | -0.43897 | 1.67E-04 |
| Tmem176a | 1.15179 | <1e-10 | **Ergic1** | -0.44003 | 1.70E-02 |
| Gm35551 | 1.148222 | 9.64E-05 | **Serp1** | -0.44078 | 4.81E-03 |
| Trbc1 | 1.144768 | 8.88E-03 | **Carhsp1** | -0.44129 | 9.79E-03 |
| Relb | 1.142155 | <1e-10 | **Ubiad1** | -0.44198 | 8.52E-03 |
| Gdpd5 | 1.140978 | <1e-10 | **Hdlbp** | -0.4437 | 1.44E-03 |
| Adh1 | 1.136915 | 1.31E-03 | **Eef2** | -0.44432 | 4.57E-03 |
| Tmem176b | 1.11652 | <1e-10 | **Lzic** | -0.44443 | 1.25E-02 |
| Kif19a | 1.115653 | 8.45E-04 | **Rps11** | -0.4449 | 2.63E-02 |
| Irf5 | 1.109939 | 3.98E-02 | **Rufy2** | -0.44527 | 1.82E-02 |
| Rin3 | 1.105714 | 1.59E-04 | **Il1rap** | -0.44717 | 1.70E-02 |
| Gm20548 | 1.101802 | 3.52E-02 | **Siah2** | -0.44949 | 2.67E-02 |
| Gsta3 | 1.100559 | 4.55E-07 | **Pdss1** | -0.44987 | 8.52E-04 |
| Ltk | 1.099431 | 2.26E-03 | **Rps14** | -0.45025 | 2.59E-02 |
| Ttc16 | 1.098498 | 1.94E-02 | **Kitl** | -0.45063 | 3.71E-02 |
| Cnpy1 | 1.097124 | 4.77E-02 | **Rpl39** | -0.4508 | 1.99E-02 |
| Gm9902 | 1.093333 | 4.96E-02 | **Eef1g** | -0.45112 | 7.86E-03 |
| Myo1f | 1.091939 | 2.74E-02 | **Pdcd4** | -0.45144 | 1.20E-03 |
| Ms4a7 | 1.090861 | 1.93E-02 | **Rpl30** | -0.45167 | 7.11E-03 |
| Micu3 | 1.088609 | 2.01E-02 | **Slc7a7** | -0.45238 | 2.15E-02 |
| Ccr1 | 1.088131 | 4.16E-02 | **Ern2** | -0.45692 | 2.51E-06 |
| Pyy | 1.087989 | 2.27E-04 | **Rpl3** | -0.45808 | 3.11E-02 |
| Cyp2d9 | 1.081252 | 8.14E-03 | **Rpl37a** | -0.45845 | 3.04E-02 |
| Il1rn | 1.079859 | 7.81E-04 | **Itpr2** | -0.46028 | 6.16E-03 |
| Eya2 | 1.078771 | 2.66E-02 | **Hsd17b7** | -0.46084 | 3.15E-02 |
| Btbd11 | 1.076652 | 5.16E-03 | **Rps29** | -0.46087 | 5.70E-03 |
| Vwa7 | 1.074128 | 4.19E-03 | **Msra** | -0.46181 | 4.92E-02 |
| 2900093K20Rik | 1.071253 | 5.78E-03 | **Bace1** | -0.46326 | 1.24E-02 |
| Icosl | 1.066878 | 9.99E-06 | **Fam107b** | -0.46336 | 2.66E-02 |
| Cd3e | 1.062843 | 9.14E-03 | **Hus1** | -0.46363 | 1.12E-02 |
| Clcn1 | 1.057432 | 4.78E-02 | **Rpl29** | -0.46483 | 3.74E-02 |
| Adam8 | 1.048622 | 1.19E-02 | **Arhgap31** | -0.46698 | 1.76E-02 |
| Atp10a | 1.047904 | 4.41E-03 | **Apol6** | -0.46805 | 2.42E-02 |
| Lama5 | 1.047366 | 5.45E-04 | **Suclg1** | -0.46832 | 8.53E-04 |
| Fam43a | 1.040688 | 1.69E-03 | **Rpl6** | -0.46961 | 2.62E-02 |
| Gbp4 | 1.037513 | 1.44E-02 | **Amigo3** | -0.47005 | 2.74E-02 |
| Nfkb2 | 1.037217 | <1e-10 | **Rplp1** | -0.4705 | 1.86E-02 |
| Arhgef6 | 1.036814 | 2.80E-02 | **Rplp2** | -0.47053 | 3.21E-02 |
| Il3ra | 1.033505 | 1.04E-03 | **Vps13c** | -0.47101 | 4.60E-02 |
| Ccdc88b | 1.032219 | 3.87E-05 | **Cpne3** | -0.47188 | 9.07E-04 |
| Dmwd | 1.031282 | 3.33E-03 | **Pdha1** | -0.47278 | 7.13E-04 |
| Mapk4 | 1.030368 | 4.83E-02 | **Zfp568** | -0.47317 | 1.64E-02 |
| Ecscr | 1.029627 | 1.97E-04 | **Prkrip1** | -0.47333 | 8.67E-03 |
| Lfng | 1.029026 | 1.56E-08 | **Adam9** | -0.47484 | 1.97E-04 |
| Cd14 | 1.021874 | 4.76E-07 | **Lsm11** | -0.47601 | 3.92E-02 |
| Ankrd37 | 1.019021 | 6.66E-03 | **Aqp11** | -0.47608 | 2.93E-02 |
| Chst11 | 1.006519 | 2.42E-04 | **Rps18** | -0.47622 | 4.65E-02 |
| Clcf1 | 1.00339 | 2.63E-02 | **Sorbs1** | -0.47661 | 1.97E-02 |
| Prkch | 1.001274 | 7.90E-03 | **Rpl35a** | -0.47666 | 2.03E-02 |
| Fmnl1 | 1.000063 | 2.76E-02 | **Rpl7** | -0.47715 | 2.99E-03 |
| Ptp4a3 | 0.999803 | 8.04E-07 | **Hmgn5** | -0.47825 | 1.17E-02 |
| Plk3 | 0.999232 | 1.38E-02 | **Rps3a1** | -0.4785 | 2.45E-02 |
| Omp | 0.99918 | 3.76E-09 | **Pdxdc1** | -0.47997 | 3.35E-07 |
| Rgs14 | 0.999137 | 4.81E-03 | **Rps3** | -0.48113 | 1.73E-02 |
| Klhdc8b | 0.99649 | 7.64E-04 | **Agr2** | -0.48171 | 3.34E-02 |
| Map3k8 | 0.994478 | 1.59E-02 | **Eef1b2** | -0.4819 | 9.82E-03 |
| Far2 | 0.99227 | 8.36E-07 | **St3gal4** | -0.48201 | 1.04E-02 |
| Noxo1 | 0.992086 | 3.19E-10 | **Rpl37** | -0.4823 | 8.58E-03 |
| Ier3 | 0.985796 | 7.68E-06 | **Lgals12** | -0.48354 | 4.00E-02 |
| Zc3h12a | 0.985607 | 1.86E-09 | **Mrpl23** | -0.48533 | 1.05E-02 |
| Cpm | 0.981625 | 5.00E-03 | **Sgpp1** | -0.48549 | 7.85E-05 |
| Cpe | 0.98071 | 9.96E-06 | **Gm12254** | -0.48613 | 4.78E-02 |
| Bok | 0.98063 | 1.75E-05 | **Fmo4** | -0.48669 | 4.96E-02 |
| Chd5 | 0.979785 | 1.24E-02 | **Sec24d** | -0.48682 | 3.50E-02 |
| A930001C03Rik | 0.979246 | 7.85E-04 | **Pdpr** | -0.48687 | 1.60E-03 |
| Nuak1 | 0.97647 | 5.33E-03 | **Rpl8** | -0.48719 | 1.61E-02 |
| Pdzd4 | 0.971452 | 4.39E-02 | **Gcc2** | -0.48737 | 5.51E-06 |
| Ptprm | 0.970255 | 1.23E-02 | **Klf11** | -0.48781 | 3.80E-02 |
| Arhgef10l | 0.965247 | 5.68E-05 | **Tbl3** | -0.48794 | 1.22E-03 |
| Ugt8a | 0.964315 | 3.99E-02 | **Tpmt** | -0.48813 | 3.53E-02 |
| Rhbdf1 | 0.961627 | 1.87E-09 | **Snx7** | -0.48949 | 1.06E-02 |
| Rras | 0.959428 | 1.58E-07 | **Gxylt1** | -0.48968 | 5.78E-04 |
| Rhbdl2 | 0.957627 | 1.09E-03 | **Ppif** | -0.4897 | 8.14E-04 |
| Gimap6 | 0.956686 | 1.89E-02 | **Gm10073** | -0.48976 | 3.43E-02 |
| Gm32358 | 0.954459 | 4.65E-02 | **Lrrc59** | -0.48982 | 1.19E-02 |
| C4bp | 0.946179 | 1.69E-04 | **Reep5** | -0.49002 | 9.30E-03 |
| Csad | 0.94263 | 3.80E-09 | **Prelid2** | -0.4909 | 2.54E-02 |
| Lama3 | 0.942432 | 4.05E-04 | **Gm15163** | -0.49267 | 2.36E-03 |
| Gimap4 | 0.93949 | 1.47E-02 | **Asns** | -0.4931 | 3.56E-02 |
| Sectm1a | 0.936525 | 4.35E-04 | **Rps27a** | -0.49372 | 1.42E-02 |
| Slc12a4 | 0.930246 | 3.82E-02 | **Got2** | -0.49377 | 5.96E-03 |
| Chrnb1 | 0.929891 | 8.49E-03 | **Rpl38** | -0.49395 | 1.75E-02 |
| Slc26a3 | 0.927524 | 1.13E-02 | **Ppargc1a** | -0.49485 | 2.07E-02 |
| Egln3 | 0.927407 | 8.73E-05 | **Rnase4** | -0.49519 | 8.75E-04 |
| Itgb2 | 0.926965 | 3.04E-03 | **Fam174b** | -0.49548 | 6.52E-03 |
| Lif | 0.924288 | 1.81E-06 | **Eef1a1** | -0.49682 | 4.88E-03 |
| Bik | 0.920519 | 1.44E-04 | **Gm8437** | -0.49901 | 2.02E-03 |
| Pfkfb3 | 0.918775 | 2.29E-03 | **Rpl12** | -0.5003 | 3.35E-02 |
| Snhg14 | 0.915716 | 1.97E-02 | **Gpsm2** | -0.50033 | 1.97E-03 |
| Tmem184c | 0.912261 | 3.46E-06 | **Gbe1** | -0.50067 | 1.25E-02 |
| H2-T10 | 0.90887 | 2.61E-02 | **Tspan13** | -0.50376 | 3.02E-04 |
| Plxnb1 | 0.906621 | 2.05E-08 | **Me2** | -0.50393 | 8.23E-05 |
| Gm49327 | 0.903983 | 2.72E-03 | **Camk1d** | -0.50497 | 3.06E-03 |
| Frs3 | 0.900739 | 1.94E-02 | **Rps5** | -0.5054 | 1.93E-02 |
| Gm16576 | 0.900007 | 1.55E-02 | **Rps2** | -0.50728 | 3.40E-02 |
| Hdhd3 | 0.900001 | 2.35E-06 | **Dglucy** | -0.5076 | 3.05E-02 |
| Ncmap | 0.895508 | 1.93E-02 | **Acads** | -0.50945 | 1.86E-03 |
| Bmp2 | 0.895171 | 2.77E-02 | **Cox7a2l** | -0.51068 | 7.64E-04 |
| Itgb6 | 0.893585 | 2.96E-05 | **Gfpt1** | -0.51105 | 7.51E-06 |
| Gstp3 | 0.892257 | 1.03E-04 | **Rpl36** | -0.51111 | 3.10E-02 |
| Birc3 | 0.889568 | 2.82E-07 | **Rps16** | -0.51165 | 2.22E-02 |
| Usp35 | 0.887045 | 1.38E-03 | **Galnt6** | -0.51229 | 5.41E-03 |
| Gm12320 | 0.883021 | 4.51E-02 | **Cbs** | -0.51251 | 3.48E-02 |
| Tm2d3 | 0.880774 | 9.02E-10 | **Prps2** | -0.51304 | 1.15E-02 |
| Hrh1 | 0.875537 | 5.65E-03 | **Aldh9a1** | -0.51321 | 7.81E-04 |
| Gsdmd | 0.87323 | 2.65E-06 | **Rps15a** | -0.51382 | 2.26E-02 |
| Pdzd2 | 0.868084 | 7.16E-03 | **Adi1** | -0.51459 | 9.70E-03 |
| Crlf2 | 0.867927 | 3.93E-05 | **H1f0** | -0.51522 | 5.10E-04 |
| Ffar4 | 0.864914 | 7.74E-05 | **Rps23** | -0.51534 | 9.24E-03 |
| Nod2 | 0.864638 | 1.07E-04 | **Tox** | -0.5154 | 1.26E-03 |
| Tmem132a | 0.862211 | 2.95E-03 | **Casp6** | -0.51808 | 3.11E-02 |
| Pmaip1 | 0.861456 | 1.18E-02 | **Myadm** | -0.51909 | 1.53E-02 |
| Tmem86a | 0.860541 | 1.98E-06 | **Lss** | -0.51977 | 1.64E-02 |
| Itgb7 | 0.858738 | 2.58E-02 | **Rpl23a** | -0.52006 | 3.63E-02 |
| S1pr2 | 0.856592 | 5.69E-03 | **Gm4332** | -0.52012 | 1.08E-02 |
| Gm47218 | 0.856428 | 1.25E-02 | **Inpp1** | -0.52013 | 6.42E-03 |
| Gabbr1 | 0.856214 | 9.51E-04 | **Gmds** | -0.52013 | 2.17E-02 |
| Scg5 | 0.853828 | 1.44E-02 | **Gpd1l** | -0.52263 | 2.49E-06 |
| Vwf | 0.849085 | 4.31E-02 | **Rap1gap** | -0.52276 | 2.20E-03 |
| Mreg | 0.848085 | 4.00E-02 | **Brca2** | -0.52414 | 4.26E-02 |
| Srgn | 0.84718 | 3.39E-02 | **Gpam** | -0.52469 | 9.79E-03 |
| Cyp2d37-ps | 0.845008 | 1.79E-04 | **Plcxd2** | -0.52513 | 6.12E-03 |
| Lpo | 0.842134 | 7.64E-04 | **Khk** | -0.52514 | 4.00E-02 |
| Stx1a | 0.839925 | 1.43E-03 | **Tmem135** | -0.52579 | 1.25E-02 |
| Traf3 | 0.83231 | <1e-10 | **Rps27** | -0.52617 | 1.88E-03 |
| Doc2g | 0.829954 | 1.38E-03 | **Rpl26** | -0.52647 | 1.29E-02 |
| Tmed8 | 0.828617 | 6.00E-03 | **Thrb** | -0.52675 | 9.58E-04 |
| Bnip3 | 0.825553 | 2.89E-02 | **Nsa2** | -0.52705 | 4.25E-04 |
| Vill | 0.824415 | 3.57E-02 | **Thsd4** | -0.52825 | 2.89E-02 |
| Fabp5 | 0.82307 | 1.79E-04 | **Decr1** | -0.52858 | 4.29E-03 |
| Ifit2 | 0.821957 | 4.04E-02 | **Rpl4** | -0.53193 | 5.81E-03 |
| Ppp1r16b | 0.821113 | 4.15E-02 | **Wls** | -0.53354 | 1.49E-02 |
| Ifitm3 | 0.820669 | 1.76E-02 | **Ahcy** | -0.53471 | 1.24E-02 |
| Gcnt1 | 0.815967 | 3.64E-02 | **Rpl27a** | -0.53549 | 1.14E-02 |
| Tat | 0.815752 | 1.99E-02 | **Slc2a10** | -0.53554 | 1.36E-02 |
| Sdhaf1 | 0.815488 | 6.91E-07 | **Acsf2** | -0.5363 | 1.26E-02 |
| Vav3 | 0.814376 | 3.85E-04 | **Ttl** | -0.53769 | 3.35E-02 |
| Ptpn13 | 0.81385 | 2.57E-05 | **Rpl38-ps2** | -0.5392 | 3.36E-02 |
| Ssh1 | 0.809082 | 4.88E-06 | **Gm9794** | -0.53993 | 3.05E-03 |
| Spsb1 | 0.808985 | 2.97E-03 | **Pank3** | -0.54194 | 3.42E-04 |
| Ikbke | 0.805911 | <1e-10 | **Fzd7** | -0.54276 | 1.72E-03 |
| 4833438C02Rik | 0.80413 | 6.84E-07 | **Hmgcs1** | -0.54313 | 2.31E-02 |
| Gm12854 | 0.803414 | 8.88E-03 | **Rpl17** | -0.54323 | 1.29E-02 |
| Prss30 | 0.803055 | 5.43E-05 | **Gjb1** | -0.54327 | 2.58E-02 |
| Selplg | 0.800499 | 3.76E-02 | **Shank2** | -0.54434 | 1.64E-02 |
| Nid2 | 0.79812 | 4.23E-04 | **Rps7** | -0.54465 | 1.01E-02 |
| Laptm5 | 0.79701 | 2.82E-02 | **Hacd1** | -0.54484 | 1.79E-02 |
| Kyat1 | 0.79664 | 3.55E-04 | **Fdx1** | -0.54699 | 1.93E-02 |
| Htra2 | 0.793054 | 1.36E-10 | **Aldh1l1** | -0.54874 | 1.94E-02 |
| Lamc2 | 0.792758 | 9.57E-04 | **Rack1** | -0.55107 | 9.63E-03 |
| Epn2 | 0.792265 | 1.57E-04 | **Heatr5a** | -0.55133 | 3.99E-03 |
| S100a11 | 0.791889 | 4.64E-08 | **Rpl18a** | -0.55505 | 3.57E-03 |
| Il20rb | 0.79111 | 2.98E-02 | **Rps20** | -0.55596 | 1.59E-02 |
| Gpr17 | 0.789688 | 4.14E-02 | **Fam114a1** | -0.55606 | 8.47E-04 |
| Ccl27a | 0.789132 | 3.35E-02 | **Maged2** | -0.55633 | 1.84E-03 |
| Mdfic | 0.779624 | 4.04E-02 | **Fzd4** | -0.55675 | 1.85E-02 |
| Sema7a | 0.776758 | 6.45E-04 | **Rpl21** | -0.55769 | 4.72E-04 |
| Mal | 0.775927 | 4.65E-02 | **Ldb3** | -0.55846 | 2.27E-02 |
| Glipr2 | 0.77545 | 2.11E-03 | **Prnp** | -0.55849 | 3.29E-02 |
| Ing4 | 0.773236 | 4.20E-02 | **Rps24** | -0.56012 | 1.04E-02 |
| Marcksl1 | 0.771428 | 3.08E-04 | **Galnt7** | -0.56016 | 1.60E-07 |
| Pls3 | 0.770041 | 2.38E-03 | **Scrn3** | -0.56079 | 1.86E-02 |
| Slc6a7 | 0.767228 | 2.10E-02 | **Rpl31** | -0.56132 | 5.12E-03 |
| Tfrc | 0.765319 | 2.67E-03 | **Setbp1** | -0.5614 | 3.84E-02 |
| Nr1i3 | 0.763125 | 3.64E-02 | **Dtd1** | -0.5621 | 2.58E-02 |
| B9d2 | 0.758949 | 2.55E-02 | **Gfod1** | -0.56327 | 3.08E-02 |
| Zyx | 0.757604 | 3.27E-05 | **Yif1b** | -0.56346 | 3.46E-02 |
| Slc11a2 | 0.754003 | 2.78E-04 | **Zfp608** | -0.56369 | 4.00E-04 |
| Myom3 | 0.748931 | 1.11E-02 | **Rplp0** | -0.56525 | 9.30E-03 |
| Ptrh1 | 0.748857 | 1.08E-02 | **Lars2** | -0.56749 | 1.60E-02 |
| Ctsh | 0.748596 | 2.85E-10 | **Ppm1l** | -0.57019 | 3.92E-02 |
| Prdm1 | 0.744929 | 8.11E-03 | **Plaat3** | -0.57116 | 2.26E-03 |
| Anks6 | 0.742437 | 4.34E-04 | **Malrd1** | -0.57262 | 2.30E-02 |
| Neurl3 | 0.740685 | 4.68E-07 | **Gm9616** | -0.57504 | 1.91E-03 |
| Pik3cd | 0.740477 | 2.96E-02 | **Gramd2** | -0.57602 | 1.01E-03 |
| Mycl | 0.740418 | 7.33E-05 | **Pla2g12a** | -0.57739 | 1.72E-02 |
| Ly6g2 | 0.73682 | 4.88E-03 | **Syde2** | -0.57955 | 2.07E-02 |
| Dpy19l1 | 0.735662 | 3.82E-04 | **Dennd11** | -0.57984 | 9.06E-03 |
| Inpp5d | 0.734298 | 1.93E-03 | **Rpl5** | -0.58109 | 3.57E-03 |
| Pla2g3 | 0.73242 | 3.08E-02 | **Tmppe** | -0.58165 | 1.08E-02 |
| Rdh10 | 0.731589 | 1.62E-04 | **Kyat3** | -0.58244 | 3.52E-02 |
| Mycbpap | 0.730571 | 2.86E-03 | **Rpl35** | -0.584 | 2.44E-02 |
| Parp3 | 0.730013 | 3.32E-03 | **Tcea3** | -0.58441 | 2.56E-04 |
| Aadac | 0.727395 | 3.63E-02 | **Rpl14-ps1** | -0.58479 | 2.00E-02 |
| Resp18 | 0.72556 | 1.88E-02 | **Gk5** | -0.58739 | 1.13E-02 |
| Glb1l | 0.725309 | 3.13E-02 | **Rpl32** | -0.58986 | 2.29E-02 |
| Dok1 | 0.723522 | 4.37E-04 | **Rps6** | -0.59045 | 1.39E-02 |
| Abcc1 | 0.722571 | 3.15E-07 | **Gm609** | -0.59342 | 9.81E-03 |
| Rhoc | 0.721872 | 9.79E-03 | **5330417C22Rik** | -0.59455 | 9.31E-04 |
| Tff3 | 0.720157 | 2.77E-03 | **Klk1** | -0.59932 | 7.61E-03 |
| Sun2 | 0.716148 | 9.24E-03 | **Rpl36a-ps2** | -0.59958 | 4.36E-02 |
| AI413582 | 0.715475 | 5.45E-04 | **Gk** | -0.60026 | 8.63E-03 |
| Tm4sf20 | 0.714956 | 3.10E-02 | **Cpne8** | -0.60066 | 2.58E-02 |
| Nlrp9b | 0.714525 | 2.13E-02 | **Rpl36a** | -0.60269 | 1.10E-02 |
| AU041133 | 0.712895 | 3.27E-02 | **2310022B05Rik** | -0.60304 | 6.73E-09 |
| Pdzk1ip1 | 0.708935 | 1.49E-02 | **Slco2b1** | -0.6037 | 2.79E-03 |
| Guca2a | 0.705656 | 5.17E-08 | **Rps8** | -0.60376 | 2.31E-03 |
| Zfp667 | 0.704011 | 4.96E-02 | **Rpl28** | -0.60591 | 2.96E-03 |
| Mmp15 | 0.701911 | 2.57E-04 | **Cacna2d2** | -0.60638 | 1.32E-02 |
| Nab2 | 0.700873 | 4.94E-02 | **Fabp2** | -0.60664 | 2.23E-02 |
| Sema4c | 0.69951 | 2.78E-02 | **Gm37233** | -0.61125 | 2.86E-02 |
| Tmem37 | 0.699282 | 2.40E-02 | **Rpl14** | -0.61311 | 5.99E-03 |
| Tlr2 | 0.696403 | 5.12E-03 | **Fam117b** | -0.61362 | 9.53E-05 |
| Whrn | 0.695287 | 3.92E-07 | **Rps4x** | -0.61438 | 4.29E-03 |
| Stap2 | 0.694478 | 1.41E-08 | **Rpl23** | -0.61724 | 4.93E-04 |
| Arrb2 | 0.692634 | 7.27E-03 | **Rpl13** | -0.61775 | 4.00E-03 |
| Xylt1 | 0.690495 | 8.16E-03 | **Oat** | -0.61859 | 3.81E-03 |
| 1500009L16Rik | 0.690125 | 2.86E-03 | **Cracr2a** | -0.6189 | 2.87E-03 |
| Tnfrsf1b | 0.689877 | 1.04E-03 | **Cd302** | -0.61938 | 1.87E-02 |
| Fam189a2 | 0.688445 | 2.50E-04 | **Dmd** | -0.62425 | 2.32E-04 |
| Celf3 | 0.684816 | 1.93E-02 | **Igtp** | -0.62707 | 4.81E-02 |
| Malat1 | 0.684667 | 1.69E-02 | **Maged1** | -0.62887 | 7.64E-04 |
| 2010003K11Rik | 0.68283 | 3.63E-02 | **Fads1** | -0.63065 | 8.75E-03 |
| Ctss | 0.682808 | 1.10E-02 | **Ceacam18** | -0.63582 | 3.46E-02 |
| Prkg2 | 0.682086 | 3.09E-02 | **Muc2** | -0.6407 | 9.74E-07 |
| Cd9 | 0.676242 | 1.71E-02 | **Sipa1l2** | -0.64531 | 6.63E-05 |
| Entpd2 | 0.674032 | 1.36E-04 | **Farp2** | -0.64582 | 3.58E-06 |
| Dtx3 | 0.673514 | 2.62E-02 | **Rps12** | -0.64728 | 3.56E-03 |
| Id1 | 0.672771 | 2.61E-07 | **Trp53i13** | -0.64909 | 5.85E-04 |
| Slc39a4 | 0.672622 | 1.18E-10 | **Tmem263** | -0.64965 | 9.30E-03 |
| Fuz | 0.671653 | 3.82E-04 | **Tpt1** | -0.6497 | 2.95E-07 |
| Junb | 0.669721 | 7.74E-05 | **Gm6136** | -0.6498 | 9.24E-03 |
| 5830408C22Rik | 0.669172 | 3.57E-02 | **Rps26** | -0.65265 | 2.42E-03 |
| H2-K2 | 0.66314 | 4.10E-02 | **Acat1** | -0.65407 | 6.01E-03 |
| Tcim | 0.662888 | 2.11E-06 | **Aqp1** | -0.6552 | 1.40E-03 |
| Znrf1 | 0.657226 | 3.80E-06 | **Fcna** | -0.65565 | 2.62E-02 |
| Mab21l4 | 0.656 | 1.18E-02 | **Ehd4** | -0.65759 | 5.46E-04 |
| Pik3r3 | 0.654444 | 2.38E-04 | **Tox3** | -0.65827 | 7.61E-03 |
| Fam187b | 0.65045 | 3.09E-02 | **Smco4** | -0.65871 | 1.88E-05 |
| Oplah | 0.64558 | 8.21E-08 | **A1cf** | -0.6596 | 3.88E-04 |
| B4galnt4 | 0.637849 | 1.46E-02 | **Greb1** | -0.66012 | 6.12E-03 |
| Unc13d | 0.637189 | 3.41E-05 | **Hpd** | -0.66257 | 2.46E-02 |
| Poglut3 | 0.636259 | 4.02E-05 | **Gga2** | -0.66337 | 7.99E-03 |
| Prmt2 | 0.63039 | 9.26E-06 | **Plk2** | -0.67273 | 2.72E-03 |
| Mpzl3 | 0.630228 | 8.59E-03 | **Creb3l1** | -0.67306 | 2.72E-03 |
| Lamb3 | 0.623447 | 2.56E-06 | **Acsm3** | -0.67734 | 1.29E-02 |
| Pdgfa | 0.620767 | 5.92E-04 | **Mageh1** | -0.67751 | 1.62E-02 |
| Ccdc122 | 0.617105 | 3.93E-02 | **Dusp7** | -0.68016 | 1.28E-02 |
| Tnip1 | 0.612921 | 6.99E-04 | **Tm6sf2** | -0.68338 | 1.69E-02 |
| Cast | 0.612516 | 3.63E-08 | **Klhl11** | -0.68489 | 2.05E-02 |
| Gm26532 | 0.611922 | 4.65E-02 | **Srcap** | -0.68603 | 2.14E-02 |
| Bckdhb | 0.610206 | 3.30E-02 | **Gm15564** | -0.6905 | 5.33E-03 |
| Leng1 | 0.610106 | 2.56E-03 | **Gpt2** | -0.69476 | 4.05E-04 |
| Ccdc116 | 0.609472 | 4.49E-02 | **Fgfr3** | -0.69607 | 1.33E-05 |
| Herpud1 | 0.608892 | 2.47E-09 | **Slc39a7** | -0.69651 | 5.54E-03 |
| Lcp1 | 0.604934 | 4.11E-02 | **Nrp2** | -0.70002 | 1.19E-02 |
| Pitpnm2 | 0.604198 | 3.67E-02 | **Gm12944** | -0.70014 | 1.15E-02 |
| Rab31 | 0.600838 | 3.83E-02 | **Rnf217** | -0.70043 | 4.16E-03 |
| Mov10 | 0.599518 | 2.88E-03 | **Fut4** | -0.70163 | 4.15E-07 |
| Gm36266 | 0.598865 | 3.76E-02 | **Unc5cl** | -0.70372 | 7.33E-05 |
| Atp6v0e2 | 0.598792 | 8.09E-06 | **Dpyd** | -0.70426 | 4.09E-05 |
| Mbip | 0.592208 | 1.98E-03 | **Psd3** | -0.70829 | 5.92E-04 |
| Ifngr1 | 0.591965 | 2.11E-04 | **Lap3** | -0.71053 | 1.49E-03 |
| Dnajc18 | 0.591199 | 2.43E-03 | **Pdzrn3** | -0.71154 | 1.94E-02 |
| Apbb3 | 0.590078 | 2.57E-03 | **Pnmal2** | -0.7129 | 4.69E-03 |
| Ccdc120 | 0.589235 | 3.99E-03 | **Cyp2c65** | -0.71659 | 6.12E-03 |
| Rhbdf2 | 0.588624 | <1e-10 | **Rps18-ps6** | -0.72038 | 2.80E-02 |
| Tuft1 | 0.58476 | 2.44E-04 | **Nos1ap** | -0.72468 | 2.42E-02 |
| Socs2 | 0.583303 | 1.02E-03 | **Amt** | -0.74029 | 1.12E-02 |
| Traf2 | 0.57584 | 7.73E-09 | **Steap3** | -0.74149 | 6.59E-03 |
| Pitpnm1 | 0.574452 | 3.52E-05 | **Ppara** | -0.7421 | 1.57E-03 |
| Atat1 | 0.569367 | 7.61E-03 | **Slc18a1** | -0.74377 | 3.86E-03 |
| Lpin2 | 0.56822 | 1.61E-02 | **Gmpr** | -0.74442 | 3.88E-03 |
| Fes | 0.567833 | 1.61E-02 | **Acpp** | -0.74444 | 7.81E-04 |
| Ier5 | 0.566575 | 6.17E-06 | **Sema5a** | -0.74882 | 2.91E-02 |
| Gramd1b | 0.566099 | 1.33E-02 | **Abca1** | -0.75703 | 2.74E-02 |
| Stard5 | 0.564527 | 1.66E-03 | **AI427809** | -0.76052 | 3.61E-02 |
| Etv3 | 0.563576 | 1.02E-03 | **Ptprn2** | -0.7609 | 5.27E-05 |
| Lsr | 0.561109 | 1.02E-10 | **Kcnc3** | -0.76176 | 1.85E-02 |
| Kctd12 | 0.558222 | 2.43E-02 | **Kcnk6** | -0.7622 | 9.60E-09 |
| Dnase1l2 | 0.558161 | 3.67E-02 | **Foxo6** | -0.76486 | 3.30E-02 |
| A930005H10Rik | 0.557525 | 2.55E-02 | **Impdh1** | -0.76789 | 1.85E-03 |
| Muc13 | 0.556666 | 1.41E-02 | **Hoga1** | -0.76846 | 4.72E-02 |
| Strip2 | 0.55605 | 1.85E-02 | **Piwil4** | -0.77327 | 1.13E-02 |
| Sting1 | 0.555897 | 2.67E-02 | **Sfxn2** | -0.77653 | 3.18E-04 |
| S100a6 | 0.553535 | 1.42E-02 | **Lcor** | -0.77654 | 4.28E-05 |
| Ccdc9b | 0.548998 | 1.19E-02 | **Sh3bp1** | -0.77831 | 1.97E-02 |
| 1700066B19Rik | 0.54322 | 3.69E-02 | **Tspan33** | -0.77889 | 3.46E-06 |
| Nyap1 | 0.53875 | 2.35E-02 | **Galnt5** | -0.78318 | 7.63E-06 |
| Impact | 0.538171 | 3.88E-02 | **Akr1c14** | -0.78421 | 1.29E-02 |
| Fgfr2 | 0.535182 | 2.32E-02 | **Lrat** | -0.78866 | 8.58E-03 |
| Mmp28 | 0.531208 | 2.13E-02 | **Isg20** | -0.79758 | 3.83E-05 |
| Zfp429 | 0.530802 | 1.99E-02 | **Steap1** | -0.79878 | 4.78E-02 |
| Naaa | 0.528763 | 1.05E-02 | **Rftn2** | -0.80038 | 1.84E-02 |
| Snx25 | 0.524571 | 1.67E-02 | **Prxl2b** | -0.80095 | 1.70E-05 |
| Ttll3 | 0.523993 | 2.16E-02 | **Dpf3** | -0.80198 | 1.12E-03 |
| Pdlim2 | 0.521821 | 1.23E-02 | **Sybu** | -0.80271 | 3.36E-03 |
| Mrgbp | 0.521699 | 8.01E-03 | **Hilpda** | -0.80323 | 1.56E-02 |
| Dqx1 | 0.520202 | 8.71E-03 | **Egf** | -0.80653 | 2.16E-03 |
| Nek8 | 0.516869 | 2.09E-02 | **Vnn1** | -0.80975 | 1.46E-02 |
| Eps8l2 | 0.516444 | 6.51E-03 | **Syne3** | -0.814 | 3.57E-02 |
| Cep162 | 0.515939 | 4.37E-04 | **Irgm2** | -0.81542 | 1.68E-03 |
| Gtpbp2 | 0.513754 | 4.81E-03 | **Nupr1** | -0.81887 | 1.26E-02 |
| Rhou | 0.512204 | 1.41E-02 | **Fam222a** | -0.82148 | 2.00E-02 |
| Atp11a | 0.510949 | 1.60E-03 | **Maob** | -0.82184 | 1.20E-03 |
| Plekhn1 | 0.508905 | 3.90E-03 | **Aldob** | -0.82233 | 5.12E-03 |
| Fbxl6 | 0.508359 | 1.94E-04 | **Rab43** | -0.82241 | 2.08E-02 |
| Arhgap27 | 0.508277 | 1.39E-04 | **Hnf4g** | -0.8252 | 1.87E-04 |
| Cd47 | 0.508212 | 6.34E-04 | **Slc35f2** | -0.82653 | 1.17E-03 |
| Dpysl3 | 0.507368 | 3.58E-02 | **Vipr2** | -0.82797 | 3.42E-02 |
| Zfp653 | 0.507079 | 4.92E-03 | **AW011738** | -0.82914 | 2.71E-02 |
| Stk25 | 0.501904 | 2.80E-02 | **Kdelr3** | -0.83711 | 5.79E-04 |
| Prkab2 | 0.501194 | 3.27E-02 | **1810055G02Rik** | -0.84258 | 1.64E-04 |
| Hacd2 | 0.501102 | 5.96E-04 | **Cbr2** | -0.8428 | 1.44E-04 |
| Cenpj | 0.500464 | 6.78E-03 | **Kcnh2** | -0.84435 | 2.94E-02 |
| Rc3h1 | 0.500337 | 2.05E-03 | **Mgll** | -0.84475 | 7.16E-03 |
| Ern1 | 0.500161 | 9.00E-04 | **Glod5** | -0.84588 | 8.18E-06 |
| Sat1 | 0.500076 | 1.41E-03 | **Lratd1** | -0.85512 | 1.13E-02 |
| Pigw | 0.499976 | 4.62E-02 | **Usp2** | -0.85788 | 1.15E-02 |
| Nav2 | 0.49863 | 1.24E-02 | **Selenbp2** | -0.86507 | 2.79E-04 |
| Lpar6 | 0.497971 | 1.05E-02 | **Tubal3** | -0.86683 | 9.76E-10 |
| Polr1c | 0.497361 | 1.79E-03 | **Gm10734** | -0.86696 | 1.33E-02 |
| Myl12b | 0.491714 | 1.54E-02 | **Gm12250** | -0.86779 | 3.67E-02 |
| B4galt1 | 0.491564 | 1.97E-02 | **Pdgfb** | -0.87143 | 8.07E-03 |
| Paxbp1 | 0.490321 | 8.88E-03 | **Slc9a3r2** | -0.87218 | 2.38E-04 |
| Eps8l3 | 0.490187 | 7.02E-03 | **Prelp** | -0.87692 | 3.79E-02 |
| Fat1 | 0.48788 | 8.14E-03 | **Chrnb4** | -0.87734 | 1.73E-02 |
| Zdhhc18 | 0.48593 | 2.27E-04 | **Gm1123** | -0.8785 | <1e-10 |
| Dock6 | 0.485864 | 5.00E-03 | **Sult1a1** | -0.88179 | 4.34E-04 |
| Map1s | 0.485243 | 3.18E-04 | **Tpt1-ps3** | -0.8844 | 4.89E-08 |
| 9530082P21Rik | 0.482325 | 1.81E-03 | **Gpd1** | -0.88499 | 2.56E-03 |
| Rnd3 | 0.481742 | 1.41E-03 | **Hc** | -0.88578 | 4.27E-02 |
| Entpd8 | 0.48157 | 4.29E-02 | **Ssbp2** | -0.88648 | 4.04E-02 |
| Phf1 | 0.481467 | 3.53E-02 | **Slc22a1** | -0.88683 | 3.80E-06 |
| Tapbp | 0.479654 | 1.88E-04 | **Clic6** | -0.89268 | 1.90E-02 |
| E230025N22Rik | 0.479365 | 2.82E-02 | **Zfpm1** | -0.89325 | 1.38E-02 |
| Mink1 | 0.478598 | 2.76E-09 | **Efna3** | -0.89682 | 2.23E-02 |
| Uba7 | 0.477924 | 6.02E-03 | **Palld** | -0.8981 | 9.02E-10 |
| Dapp1 | 0.477395 | 4.62E-02 | **Card11** | -0.89906 | 1.21E-04 |
| Neurl1b | 0.476457 | 5.08E-03 | **Shf** | -0.90092 | 5.03E-03 |
| Afmid | 0.475852 | 3.39E-02 | **Rps4x-ps** | -0.90328 | 3.61E-03 |
| Il6st | 0.475754 | 1.97E-04 | **Ceacam12** | -0.90685 | 1.19E-02 |
| Wdr53 | 0.474939 | 1.24E-02 | **Cand2** | -0.90704 | 1.46E-03 |
| Plgrkt | 0.474042 | 3.05E-03 | **Gal3st2** | -0.90949 | 2.57E-04 |
| Vmp1 | 0.473848 | 4.18E-03 | **Nt5dc2** | -0.90998 | 6.63E-03 |
| Csnk1e | 0.472973 | 4.37E-04 | **Mogat2** | -0.91186 | 1.70E-02 |
| Neu1 | 0.467965 | 1.23E-02 | **Cib2** | -0.91754 | 3.43E-02 |
| 2810013P06Rik | 0.465516 | 2.54E-02 | **Stc2** | -0.92568 | 7.49E-06 |
| C130074G19Rik | 0.465196 | 4.34E-03 | **Ptprt** | -0.92691 | 1.64E-03 |
| Pkp3 | 0.464911 | 4.76E-07 | **Cdr2** | -0.93159 | 4.21E-03 |
| Vps37c | 0.464553 | 2.00E-04 | **Smim10l2a** | -0.93518 | 4.77E-02 |
| BC016579 | 0.463406 | 1.05E-02 | **Chpt1** | -0.93863 | 1.60E-03 |
| Gas2l1 | 0.462637 | 5.88E-03 | **Slc43a2** | -0.94126 | 4.38E-03 |
| Hyal2 | 0.462617 | 1.74E-05 | **Adamts17** | -0.94312 | 4.27E-02 |
| Btg1 | 0.462067 | 1.33E-03 | **Slc12a8** | -0.94433 | 2.45E-09 |
| Cables2 | 0.460643 | 2.35E-05 | **Slc28a2** | -0.94465 | 5.00E-02 |
| Tchp | 0.458547 | 2.44E-02 | **Gpsm1** | -0.94472 | 3.47E-05 |
| Nectin1 | 0.458465 | 3.28E-04 | **Casc4** | -0.94658 | 1.87E-04 |
| Tmc4 | 0.457283 | 4.09E-05 | **Palmd** | -0.94737 | 1.60E-03 |
| Tmem106a | 0.45554 | 3.19E-03 | **Gcnt4** | -0.94909 | 2.40E-03 |
| Otub1 | 0.455344 | 2.58E-02 | **Dpp4** | -0.95094 | 3.05E-07 |
| Amotl2 | 0.455138 | 4.18E-02 | **Ass1** | -0.95633 | 2.09E-03 |
| Hs3st3b1 | 0.454304 | 3.05E-02 | **Sh3bp5** | -0.95651 | 6.28E-04 |
| Pdia5 | 0.453812 | 2.26E-03 | **Rab3c** | -0.95926 | 3.50E-02 |
| Mt1 | 0.453464 | 1.12E-02 | **Gm6192** | -0.96074 | 1.97E-02 |
| Spns1 | 0.452763 | 4.81E-04 | **Spink4** | -0.96166 | 1.06E-03 |
| Ddit4 | 0.451321 | 6.04E-04 | **Gm13657** | -0.96817 | 1.54E-02 |
| Prr3 | 0.448334 | 4.98E-02 | **Ace** | -0.96977 | 6.62E-03 |
| Zfr2 | 0.4465 | 9.99E-03 | **Armh4** | -0.9717 | 3.29E-02 |
| Cdc14b | 0.44648 | 4.85E-02 | **Coq8a** | -0.97299 | 3.35E-07 |
| 1810026B05Rik | 0.446282 | 2.61E-02 | **B3gnt9** | -0.97446 | 4.51E-03 |
| Ccdc68 | 0.442007 | 3.55E-02 | **Kcnd1** | -0.97463 | 2.73E-03 |
| Rilpl2 | 0.439928 | 1.62E-02 | **Hipk2** | -0.97754 | 1.57E-04 |
| Zhx2 | 0.437877 | 1.96E-02 | **Hao2** | -0.98087 | 4.67E-06 |
| Trp53 | 0.435768 | 5.08E-03 | **Zfhx3** | -0.98453 | 3.56E-04 |
| 3830406C13Rik | 0.435749 | 1.14E-02 | **Spsb4** | -0.98798 | 2.55E-02 |
| Abca7 | 0.435726 | 1.29E-03 | **Depdc7** | -0.98998 | 1.51E-02 |
| Gm20559 | 0.431775 | 2.00E-02 | **Ptger3** | -0.99561 | 4.92E-03 |
| Pea15a | 0.431459 | 3.70E-03 | **Slc7a9** | -0.99597 | 7.33E-05 |
| Zfp160 | 0.428575 | 2.78E-02 | **Rgcc** | -0.99722 | 1.52E-03 |
| Aplp1 | 0.42723 | 8.91E-03 | **Mfap3l** | -0.99923 | 1.06E-03 |
| Igsf9b | 0.423359 | 1.36E-02 | **Pycr1** | -1.00093 | 1.67E-03 |
| Crybg2 | 0.423195 | 1.37E-02 | **Trim38** | -1.00211 | 4.09E-03 |
| Nectin2 | 0.422849 | 3.85E-04 | **Agbl2** | -1.00211 | 2.01E-02 |
| Mapk7 | 0.420237 | 9.96E-04 | **4930447F24Rik** | -1.00436 | 5.78E-03 |
| Ccdc66 | 0.41791 | 3.88E-02 | **Tenm4** | -1.00813 | 5.49E-04 |
| Rfx5 | 0.417526 | 4.49E-04 | **Vwa1** | -1.00975 | 1.07E-02 |
| Scly | 0.417425 | 1.62E-02 | **Anxa13** | -1.01581 | 1.32E-05 |
| Megf8 | 0.415713 | 1.24E-02 | **Mcub** | -1.01823 | 6.85E-04 |
| Cnksr1 | 0.415504 | 2.83E-03 | **Eml1** | -1.02354 | 2.93E-02 |
| Ripk3 | 0.415288 | 2.14E-02 | **Cgref1** | -1.0251 | 1.74E-05 |
| Sufu | 0.411656 | 2.26E-03 | **Rn18s‑rs5** | -1.03671 | 5.86E-04 |
| Grpel2 | 0.411549 | 1.08E-03 | **Plet1** | -1.0383 | 2.55E-04 |
| Igf2bp2 | 0.409724 | 6.90E-08 | **Kcnh3** | -1.03973 | 6.18E-07 |
| Prpf40b | 0.407381 | 3.01E-02 | **Nr2e3** | -1.04007 | 1.09E-02 |
| Sbno2 | 0.406433 | 1.46E-02 | **Etv5** | -1.04284 | 2.10E-06 |
| Mafg | 0.404723 | 1.97E-02 | **Rab11fip5** | -1.04948 | 2.88E-02 |
| Rtkn | 0.402689 | 1.66E-04 | **Copz2** | -1.04959 | 5.00E-03 |
| Mapk8ip3 | 0.401613 | 1.16E-02 | **Creb3l3** | -1.0571 | 2.23E-02 |
| Tmem82 | 0.398589 | 7.33E-05 | **Gpr20** | -1.05851 | 4.43E-06 |
| Dcaf15 | 0.397329 | 7.34E-04 | **Slc7a8** | -1.05877 | 1.84E-03 |
| Samd1 | 0.395695 | 7.13E-04 | **Erich4** | -1.06148 | 3.06E-04 |
| Sema4b | 0.395664 | 1.28E-02 | **Bmp7** | -1.07465 | 8.39E-03 |
| Ndst2 | 0.394054 | 4.21E-03 | **Nr4a2** | -1.07854 | 1.94E-02 |
| Npc1 | 0.393884 | 4.89E-02 | **H2-DMb1** | -1.08355 | 4.71E-03 |
| Mief2 | 0.39329 | 1.97E-02 | **Fstl3** | -1.0841 | 5.33E-03 |
| Nbeal2 | 0.393238 | 4.80E-03 | **Nanos1** | -1.09451 | 3.43E-02 |
| Tjp3 | 0.39298 | 1.03E-10 | **Fam83c** | -1.0971 | 1.06E-03 |
| Rai14 | 0.392564 | 3.70E-03 | **Pyroxd2** | -1.10376 | 4.18E-08 |
| Bcl2l12 | 0.391858 | 3.42E-02 | **D930020B18Rik** | -1.11586 | 4.08E-02 |
| Arhgef40 | 0.391701 | 1.46E-02 | **Ms4a18** | -1.12194 | 9.24E-03 |
| Ppox | 0.391638 | 3.43E-02 | **Rasgrf2** | -1.13225 | 1.17E-04 |
| Alkbh6 | 0.38949 | 6.14E-03 | **Sult1c2** | -1.13562 | 4.59E-09 |
| Cchcr1 | 0.388658 | 2.89E-02 | **Upb1** | -1.14553 | 8.49E-03 |
| Pogk | 0.387791 | 1.61E-02 | **Efr3b** | -1.14726 | 5.12E-03 |
| Tnk1 | 0.387484 | 2.67E-03 | **Tnfrsf23** | -1.15895 | 1.97E-04 |
| Cgn | 0.385244 | 1.79E-02 | **Btn1a1** | -1.15916 | 3.35E-02 |
| Adap1 | 0.383601 | 6.66E-03 | **Gm10095** | -1.16152 | 4.26E-02 |
| Ano9 | 0.382853 | 3.48E-03 | **Nxf7** | -1.16278 | 3.49E-02 |
| Lzts3 | 0.381711 | 1.97E-04 | **Col28a1** | -1.17108 | 3.92E-02 |
| Gmip | 0.381642 | 4.11E-02 | **Sst** | -1.17713 | 1.21E-03 |
| Zmym6 | 0.381512 | 2.66E-03 | **H2-Q10** | -1.17843 | 1.53E-06 |
| Fancg | 0.381166 | 1.28E-02 | **Dixdc1** | -1.18164 | 1.25E-06 |
| Wdyhv1 | 0.380766 | 1.34E-02 | **Sardh** | -1.19044 | 8.47E-06 |
| Ap5z1 | 0.379565 | 3.74E-02 | **Myrf** | -1.19374 | 1.02E-03 |
| Krit1 | 0.375646 | 4.50E-02 | **Lpl** | -1.19958 | 6.05E-05 |
| Dapk2 | 0.374265 | 8.13E-03 | **Ntrk2** | -1.19993 | 2.76E-03 |
| Nxf1 | 0.373166 | 1.70E-02 | **Gm1965** | -1.20505 | 1.12E-02 |
| Plscr3 | 0.3721 | 1.20E-02 | **Gm16863** | -1.20697 | 2.58E-02 |
| Mtmr11 | 0.37197 | 2.77E-02 | **Fads6** | -1.21265 | 1.61E-02 |
| Mettl17 | 0.370497 | 3.25E-02 | **Prlr** | -1.2166 | <1e-10 |
| Nfkbil1 | 0.369026 | 1.70E-02 | **Slc16a2** | -1.21979 | 1.14E-02 |
| Zfp217 | 0.368091 | 3.39E-02 | **Arhgap24** | -1.22154 | 4.66E-05 |
| Stoml1 | 0.368068 | 1.19E-02 | **Akr1c18** | -1.23619 | 2.27E-02 |
| Ppp1r16a | 0.367911 | 1.48E-02 | **E230034D01Rik** | -1.2406 | 1.16E-03 |
| Eppk1 | 0.367757 | 3.04E-02 | **Asah2** | -1.24278 | 1.17E-04 |
| Wsb1 | 0.36653 | 3.13E-02 | **Stard8** | -1.24623 | 3.52E-04 |
| Trim7 | 0.366325 | 3.71E-02 | **Naaladl1** | -1.24995 | <1e-10 |
| Dvl1 | 0.364299 | 1.14E-02 | **Clps** | -1.25936 | 1.33E-03 |
| Cd82 | 0.363951 | 3.66E-02 | **Soat2** | -1.26075 | 2.42E-04 |
| Tmc6 | 0.363542 | 1.12E-02 | **Nxpe2** | -1.27078 | 1.52E-05 |
| Anxa7 | 0.363212 | 1.79E-02 | **Sp5** | -1.27504 | 3.43E-02 |
| Fam98c | 0.361099 | 2.81E-02 | **Gatm** | -1.27947 | 5.70E-03 |
| Bicd2 | 0.360937 | 5.08E-03 | **Gnat2** | -1.28579 | 2.22E-02 |
| Vamp4 | 0.359507 | 3.19E-02 | **Maf** | -1.28607 | 7.84E-07 |
| Baiap2l2 | 0.357964 | 7.94E-03 | **Gata4** | -1.29518 | 1.46E-02 |
| Rassf6 | 0.357373 | 3.14E-02 | **Gcg** | -1.30433 | 1.63E-04 |
| Prkd2 | 0.35682 | 1.05E-02 | **Myrfl** | -1.3409 | 3.56E-06 |
| Id3 | 0.356151 | 4.07E-03 | **Fhit** | -1.34538 | 9.02E-04 |
| Fermt1 | 0.353735 | 2.77E-04 | **Ly6e** | -1.35169 | 2.45E-09 |
| Tfe3 | 0.352229 | 1.41E-02 | **Mfsd4b2** | -1.35514 | 8.13E-04 |
| Cdc42bpa | 0.351463 | 5.88E-03 | **Tpd52l1** | -1.35533 | 7.65E-05 |
| Fbxo33 | 0.3501 | 7.00E-03 | **Sec14l2** | -1.36404 | 8.58E-03 |
| Stx16 | 0.348953 | 1.90E-02 | **Tnfaip8l3** | -1.36814 | 1.49E-02 |
| Tmem238l | 0.346588 | 7.00E-03 | **Zfp9** | -1.3711 | <1e-10 |
| Pnkp | 0.345381 | 7.43E-03 | **Gsdmcl-ps** | -1.38021 | 2.28E-02 |
| Zdhhc12 | 0.345381 | 8.73E-03 | **Slc26a6** | -1.38892 | 1.42E-02 |
| Zfand5 | 0.344551 | 3.57E-02 | **Gpr155** | -1.39103 | 3.85E-02 |
| Bicra | 0.34431 | 2.62E-02 | **Npl** | -1.39371 | 2.23E-02 |
| Naprt | 0.344123 | 3.63E-02 | **AI838599** | -1.4072 | 1.58E-03 |
| Mib2 | 0.341418 | 3.35E-02 | **Iapp** | -1.42801 | 3.99E-02 |
| Mvp | 0.341197 | 4.53E-05 | **Apob** | -1.43496 | 3.19E-08 |
| Rps6kb2 | 0.341121 | 1.17E-02 | **Smpdl3b** | -1.44339 | 7.78E-07 |
| Pgpep1 | 0.340392 | 1.64E-02 | **Dab1** | -1.44488 | 4.80E-03 |
| Neil1 | 0.338991 | 1.68E-02 | **Syngr1** | -1.45744 | 4.16E-05 |
| Zfp219 | 0.338559 | 1.64E-02 | **Gm3054** | -1.46812 | 3.85E-02 |
| Fam214a | 0.337389 | 4.02E-02 | **Pex5l** | -1.4727 | 7.30E-06 |
| Rchy1 | 0.336532 | 9.55E-05 | **Slc16a10** | -1.47642 | 3.72E-07 |
| Zfp276 | 0.333253 | 1.76E-02 | **Olfr78** | -1.48266 | 9.55E-05 |
| Med23 | 0.332918 | 4.46E-04 | **Fmo1** | -1.48954 | 2.24E-03 |
| Gatd1 | 0.331747 | 3.06E-03 | **Syna** | -1.49305 | 7.96E-03 |
| Dmap1 | 0.331561 | 8.59E-03 | **Fzd9** | -1.49379 | 7.31E-03 |
| Usf2 | 0.330923 | 2.78E-03 | **Ada** | -1.49609 | 3.88E-02 |
| Plbd2 | 0.327535 | 1.11E-03 | **Agmo** | -1.51952 | 1.59E-09 |
| E4f1 | 0.326328 | 4.36E-02 | **Abcc2** | -1.52574 | 1.18E-02 |
| Bspry | 0.326245 | 1.04E-03 | **Slc16a12** | -1.52944 | 4.37E-04 |
| Lyrm9 | 0.323896 | 4.22E-02 | **Aldh1l2** | -1.52952 | 4.51E-03 |
| Myo19 | 0.323809 | 4.74E-02 | **Enpep** | -1.54598 | 4.35E-04 |
| Tnfrsf11a | 0.321503 | 3.05E-02 | **Grpr** | -1.54867 | 1.42E-02 |
| Man2c1 | 0.319473 | 1.68E-02 | **Ephx4** | -1.54894 | 7.16E-04 |
| Rassf7 | 0.318393 | 8.13E-03 | **Kif26a** | -1.55152 | 1.47E-02 |
| Hdgfl2 | 0.318357 | 6.63E-03 | **Vwce** | -1.55246 | 3.05E-03 |
| E2f2 | 0.31681 | 4.20E-02 | **Zyg11a** | -1.55905 | 3.88E-02 |
| Gstm5 | 0.316794 | 4.43E-02 | **Hapln4** | -1.58512 | 1.06E-03 |
| Hook2 | 0.315176 | 9.47E-03 | **Klhl13** | -1.58765 | 4.20E-02 |
| Arrdc1 | 0.312389 | 1.25E-02 | **Sstr1** | -1.59713 | 3.94E-04 |
| Colgalt1 | 0.31172 | 2.15E-04 | **Miat** | -1.60556 | 1.67E-04 |
| Recql5 | 0.311499 | 2.12E-02 | **Adcy8** | -1.64305 | 3.45E-02 |
| Commd4 | 0.311261 | 5.00E-03 | **Casp14** | -1.64831 | 3.43E-02 |
| Sirt7 | 0.311221 | 9.90E-03 | **Tesc** | -1.70359 | 1.67E-04 |
| Zfp513 | 0.310133 | 2.64E-02 | **Ndnf** | -1.71192 | 9.99E-03 |
| Erbb2 | 0.305108 | 3.04E-02 | **Cps1** | -1.71639 | 2.81E-04 |
| Skiv2l | 0.304129 | 9.24E-03 | **Pbld1** | -1.71992 | 7.83E-05 |
| Tmem134 | 0.3022 | 5.65E-03 | **Fabp6** | -1.72098 | 2.22E-02 |
| Spint2 | 0.301745 | 2.55E-02 | **Alppl2** | -1.74058 | 5.12E-03 |
| Pbxip1 | 0.300711 | 1.60E-02 | **Nexmif** | -1.74164 | 1.43E-03 |
| Kazn | 0.297551 | 1.39E-02 | **Acot12** | -1.76168 | 3.28E-02 |
| Map4k4 | 0.297322 | 2.09E-02 | **Insrr** | -1.77885 | 3.80E-06 |
| Spint1 | 0.296505 | 4.89E-02 | **Scara5** | -1.77899 | 9.80E-04 |
| Rela | 0.295673 | 2.93E-02 | **Mt3** | -1.78077 | 1.22E-03 |
| Nfkb1 | 0.294536 | 6.20E-03 | **Dclk3** | -1.79463 | 5.20E-04 |
| Zfp598 | 0.293914 | 1.00E-03 | **Ccn3** | -1.79755 | 2.00E-04 |
| Fzr1 | 0.292733 | 3.41E-03 | **Slc4a5** | -1.81583 | 3.76E-02 |
| Aplp2 | 0.287367 | 1.99E-02 | **Tmem45a** | -1.81722 | 5.65E-03 |
| Parp2 | 0.283922 | 1.73E-02 | **Slc6a19** | -1.83371 | 1.26E-02 |
| Pdzd3 | 0.283903 | 4.41E-02 | **Gm11127** | -1.83431 | 1.02E-02 |
| Lrrc45 | 0.283673 | 1.25E-02 | **Cd36** | -1.84414 | 9.19E-04 |
| Asb6 | 0.283138 | 2.54E-02 | **Gsdmc4** | -1.8504 | 4.80E-03 |
| B4galt3 | 0.28289 | 5.83E-03 | **Eda2r** | -1.87029 | 6.01E-03 |
| Unc93b1 | 0.282378 | 3.70E-03 | **Chrm3** | -1.87964 | 3.80E-06 |
| Phgr1 | 0.278332 | 1.20E-02 | **9030619P08Rik** | -1.89952 | 8.24E-04 |
| Ttc4 | 0.277179 | 8.55E-03 | **Abcg8** | -1.90162 | 8.85E-06 |
| R3hdm4 | 0.270874 | 3.08E-03 | **Otc** | -1.90711 | <1e-10 |
| Yrdc | 0.270362 | 4.81E-02 | **Pvt1** | -1.91379 | 1.05E-02 |
| Senp3 | 0.269929 | 8.26E-03 | **Ggt1** | -1.95278 | 5.33E-05 |
| Usf1 | 0.267688 | 3.76E-02 | **Slc34a1** | -1.97254 | 1.64E-02 |
| Rexo1 | 0.266163 | 1.43E-02 | **Sypl2** | -1.99236 | 7.39E-05 |
| Ap1g2 | 0.262716 | 3.09E-02 | **2010106E10Rik** | -1.99976 | 5.12E-03 |
| Zbtb18 | 0.261739 | 2.54E-02 | **Gm23935** | -2.00641 | 8.65E-03 |
| Tada1 | 0.258518 | 2.74E-02 | **Cabp1** | -2.02843 | 1.89E-02 |
| Plekhg3 | 0.258248 | 4.37E-02 | **C2cd4a** | -2.03212 | 2.73E-03 |
| Atp9b | 0.253719 | 1.18E-02 | **Cyp4x1** | -2.06192 | 4.70E-03 |
| Fbxl19 | 0.253243 | 1.41E-02 | **Susd2** | -2.06602 | 3.52E-02 |
| Camta2 | 0.251179 | 3.37E-02 | **Gsdmc2** | -2.06885 | 4.09E-03 |
| Lmnb2 | 0.248279 | 1.97E-02 | **Rragd** | -2.11816 | 1.04E-03 |
| Polrmt | 0.245496 | 4.13E-02 | **Treh** | -2.12426 | 3.74E-02 |
| Foxp4 | 0.245065 | 4.06E-02 | **Nat8f5** | -2.18203 | 1.83E-02 |
| Tspan15 | 0.244846 | 7.31E-03 | **Enpp3** | -2.19273 | 6.64E-03 |
| Cpsf1 | 0.244523 | 2.35E-02 | **Nptx1** | -2.20459 | 1.12E-03 |
| Sestd1 | 0.24343 | 2.91E-02 | **Gm8714** | -2.20799 | 2.79E-02 |
| Ggt6 | 0.243152 | 1.13E-02 | **Nrn1** | -2.21061 | 1.55E-05 |
| Fbxw5 | 0.239657 | 4.21E-02 | **Ace2** | -2.21908 | 2.30E-04 |
| Zbtb17 | 0.238916 | 2.53E-02 | **Asb16** | -2.24822 | 1.44E-04 |
| Fibp | 0.236597 | 4.74E-02 | **Abcg5** | -2.25068 | <1e-10 |
| Ranbp3 | 0.230528 | 1.65E-03 | **Rdh7** | -2.25202 | 3.37E-02 |
| Oga | 0.224521 | 1.31E-02 | **Prrx1** | -2.25538 | 1.70E-02 |
| Vps28 | 0.223713 | 4.82E-02 | **Mme** | -2.26387 | 2.33E-02 |
| Lzts2 | 0.211905 | 4.53E-02 | **Nccrp1** | -2.28565 | 1.79E-05 |
| Vps52 | 0.202819 | 1.90E-02 | **Reg3a** | -2.286 | 6.45E-04 |
| Cdk9 | 0.201664 | 1.12E-02 | **Iigp1** | -2.32174 | 2.14E-04 |
| Fxr2 | 0.19456 | 4.61E-02 | **Anpep** | -2.41953 | 2.71E-03 |
| Aggf1 | 0.190131 | 3.99E-02 | **Apol10c-ps** | -2.43182 | 5.43E-05 |
| Ehmt2 | 0.159872 | 2.42E-02 | **Adh6a** | -2.4344 | 2.52E-02 |
| Camsap3 | -0.17241 | 1.05E-02 | **Klf15** | -2.43906 | 1.10E-07 |
| Brd1 | -0.19334 | 3.98E-02 | **Aqp7** | -2.44241 | 1.38E-04 |
| Mindy1 | -0.19504 | 7.28E-03 | **Mttp** | -2.45712 | 5.88E-03 |
| Cggbp1 | -0.19723 | 2.02E-02 | **Pdzk1** | -2.46985 | 1.08E-02 |
| Wdr48 | -0.19831 | 3.99E-02 | **Apoa4** | -2.48081 | 4.85E-02 |
| Ptdss1 | -0.20155 | 3.88E-02 | **Sis** | -2.48193 | 4.57E-04 |
| Lig3 | -0.20747 | 3.99E-02 | **Nat8** | -2.50428 | 1.05E-02 |
| Gopc | -0.21502 | 4.77E-02 | **Fabp1** | -2.59869 | 6.50E-03 |
| Pmm2 | -0.21666 | 6.50E-04 | **Apoc3** | -2.60169 | 1.64E-02 |
| Slc30a7 | -0.21951 | 3.74E-02 | **Slc5a4a** | -2.62071 | 3.16E-04 |
| Itpr3 | -0.22017 | 3.42E-02 | **Gimd1** | -2.63327 | 3.08E-02 |
| Nectin3 | -0.22225 | 3.32E-02 | **Slc5a11** | -2.63494 | 1.90E-02 |
| Pcsk7 | -0.22316 | 3.43E-02 | **Slc30a2** | -2.64609 | 8.08E-04 |
| Stt3b | -0.22383 | 4.22E-02 | **Apoa1** | -2.65239 | 2.43E-02 |
| Mapk9 | -0.22513 | 3.06E-02 | **Bcan** | -2.65618 | 4.15E-05 |
| Dap3 | -0.22664 | 4.96E-02 | **Cyp2b10** | -2.6562 | 2.70E-02 |
| Papola | -0.22979 | 8.01E-03 | **Npc1l1** | -2.67025 | 4.07E-03 |
| Tspan7 | -0.23055 | 4.36E-02 | **Ocm** | -2.69794 | 1.42E-02 |
| Morf4l1 | -0.23157 | 2.42E-02 | **Agmat** | -2.7642 | 3.99E-03 |
| Ccny | -0.23236 | 3.56E-02 | **Cyp3a11** | -2.77725 | 2.72E-02 |
| Uqcrc2 | -0.23269 | 4.54E-02 | **Ugt2a3** | -2.77734 | 1.57E-02 |
| Golph3l | -0.23518 | 3.64E-02 | **Gip** | -2.80617 | 2.15E-02 |
| Hnrnpul1 | -0.23831 | 6.76E-03 | **Cyp3a44** | -2.93168 | 1.64E-03 |
| Eif4ebp2 | -0.23909 | 1.99E-02 | **Lrrc75b** | -3.04613 | 2.96E-05 |
| Mrpl9 | -0.24069 | 4.02E-02 | **Cyp3a25** | -3.11037 | 3.56E-04 |
| Mlxip | -0.24073 | 4.37E-04 | **G6pc** | -3.14314 | 3.80E-02 |
| Gnl3l | -0.24113 | 4.26E-02 | **Mptx1** | -3.16128 | 1.14E-04 |
| Itga6 | -0.24189 | 1.97E-02 | **Pla2g4c** | -3.17233 | 5.00E-03 |
| Tmed2 | -0.24344 | 2.06E-02 | **Enpp7** | -3.19747 | 1.87E-02 |
| Idh3b | -0.24366 | 4.02E-02 | **Fam151a** | -3.27079 | 4.67E-02 |
| Stk38l | -0.24712 | 4.22E-02 | **Ang4** | -3.41497 | 9.55E-05 |
| Dnajc1 | -0.24872 | 3.76E-02 | **Pcdh17** | -3.41749 | <1e-10 |
| Pccb | -0.25565 | 1.24E-02 | **Unc93a** | -3.69645 | 1.36E-02 |
| Fam120a | -0.25711 | 3.29E-02 | **Spta1** | -3.84736 | 4.42E-02 |
| Poldip2 | -0.25796 | 1.25E-02 | **Plb1** | -3.88297 | 1.97E-02 |
| Magt1 | -0.26031 | 3.99E-02 | **Mbl2** | -4.45431 | 9.68E-03 |
| Tfam | -0.26168 | 4.17E-02 | **Retnlb** | -4.78346 | 4.51E-08 |
| Mrpl15 | -0.26254 | 3.45E-02 | **Kdm5d** | -10.9528 | 5.49E-03 |
| Tfdp2 | -0.26468 | 4.81E-02 | **Eif2s3y** | -11.0077 | 2.12E-02 |
| Immt | -0.26506 | 2.58E-02 | **Ddx3y** | -23.6968 | 9.02E-10 |
| Tpd52 | -0.26593 | 9.86E-03 |  |  |  |
