# Supplementary figures and images for: CARD14 signaling in intestinal epithelial cells induces intestinal inflammation and intestinal transit delay
Source: EMBO Mol Med. 2025 Oct 23;17(12):3300–28. doi: 10.1038/s44321-025-00321-4 (PMC12686530; doi:10.1038/s44321-025-00321-4)

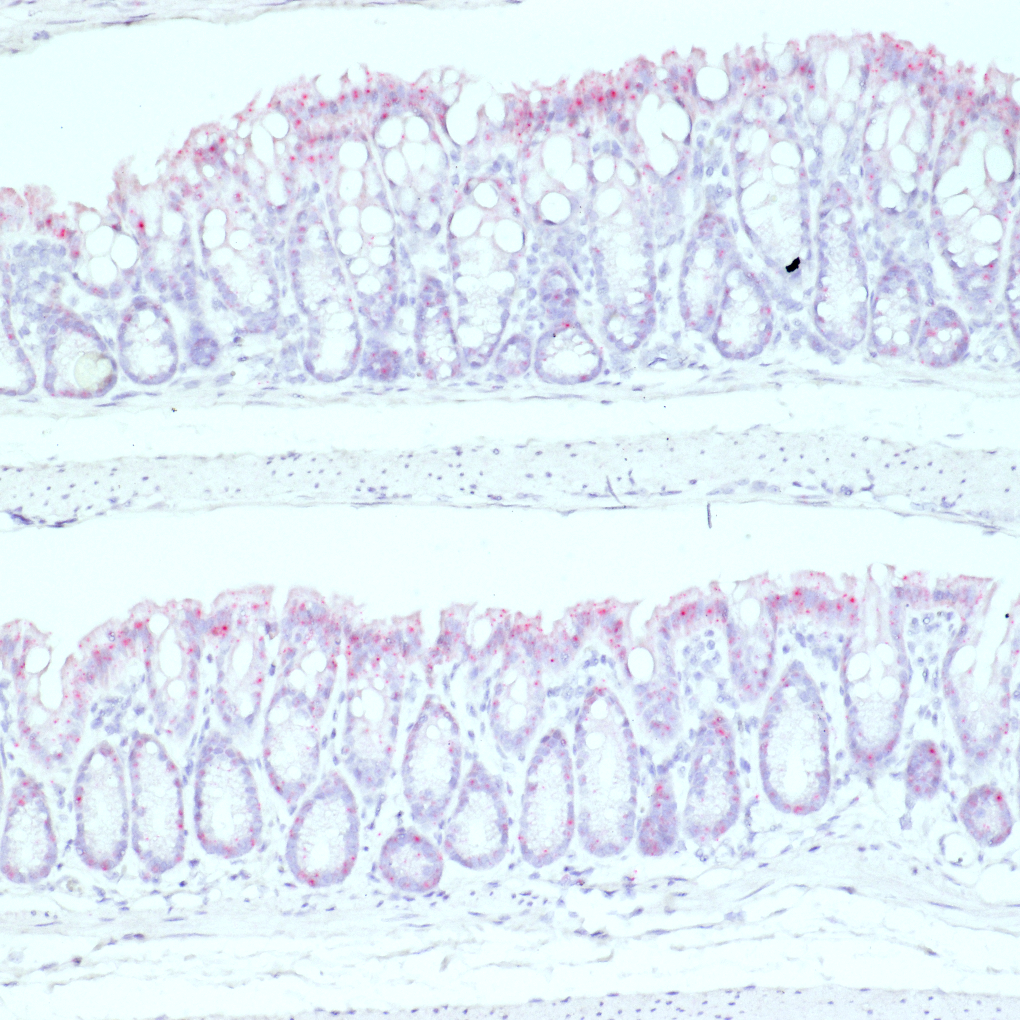

Supplement: Supplementary file 4 — Source data Fig. 1 [file 44321_2025_321_MOESM4_ESM.zip › Figure 1/1A/1A RNA scope Colon.tif]

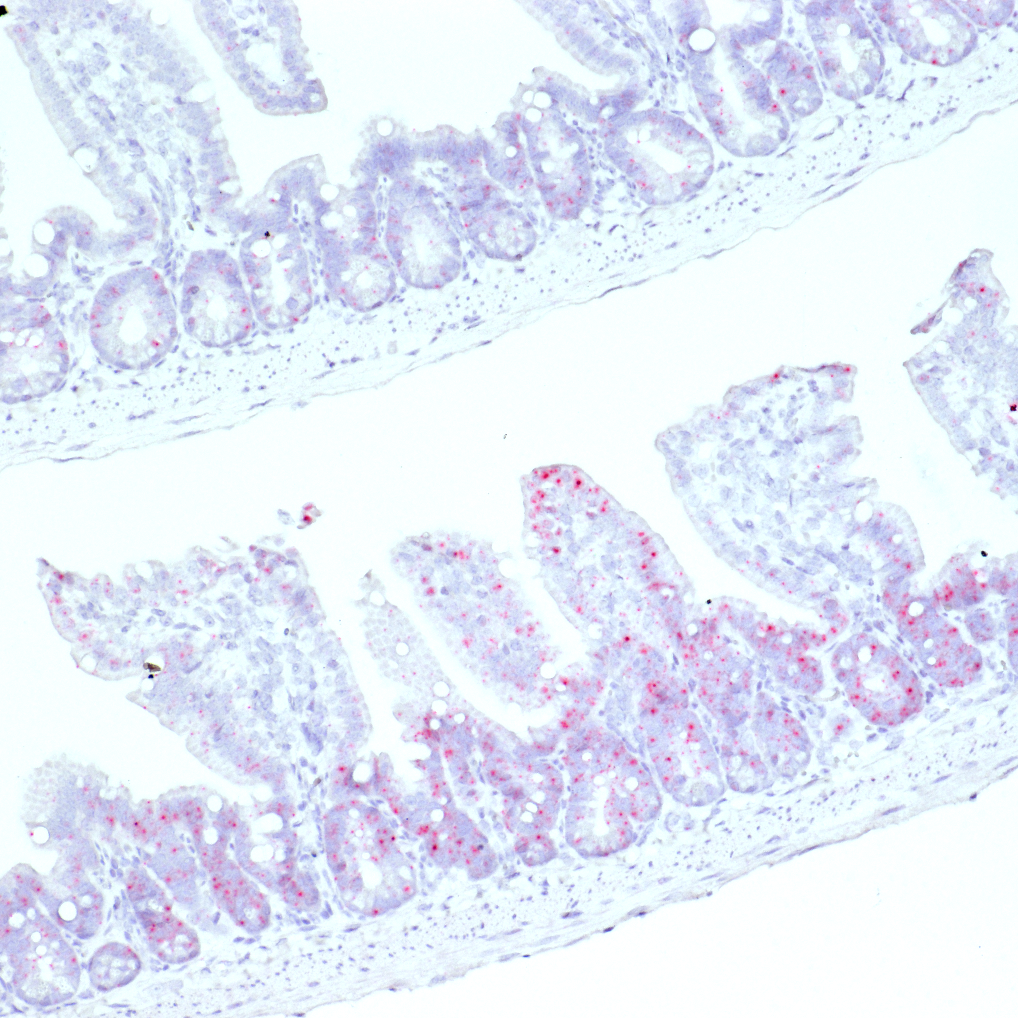

Supplement: Supplementary file 4 — Source data Fig. 1 [file 44321_2025_321_MOESM4_ESM.zip › Figure 1/1A/1A RNA scope Small intestine.tif]

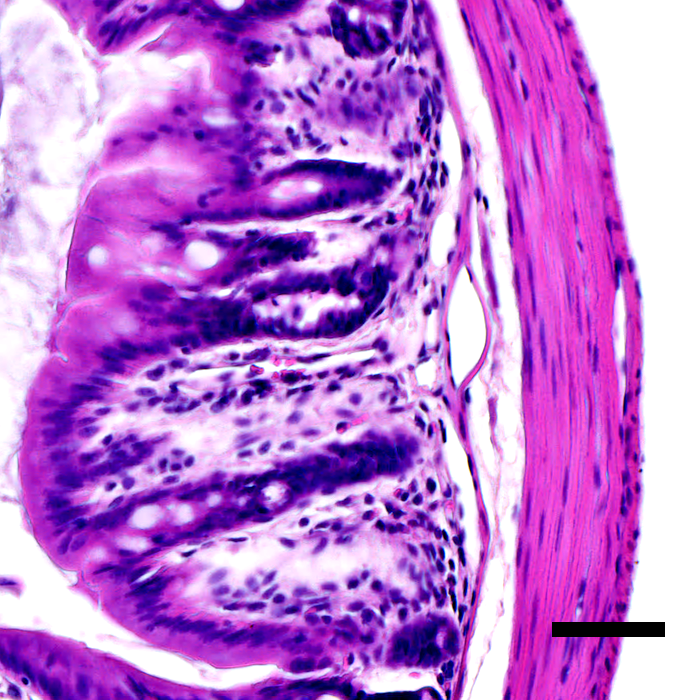

Supplement: Supplementary file 4 — Source data Fig. 1 [file 44321_2025_321_MOESM4_ESM.zip › Figure 1/1B/1B Colon E138A cropped scale bar.tif]

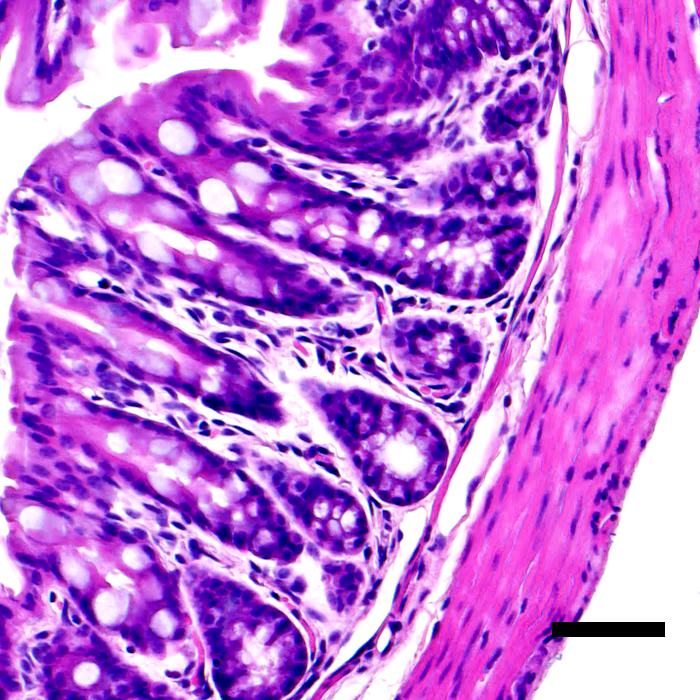

Supplement: Supplementary file 4 — Source data Fig. 1 [file 44321_2025_321_MOESM4_ESM.zip › Figure 1/1B/1B Colon WT cropped scale bar.tif]

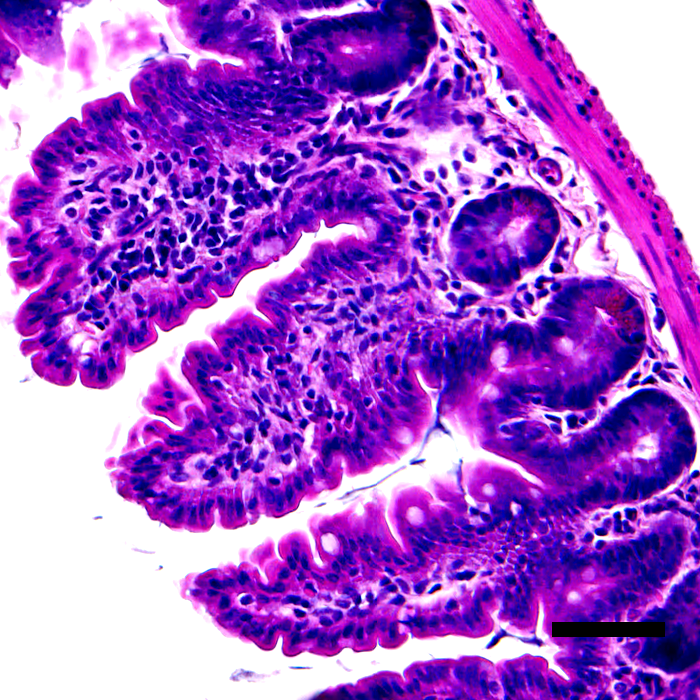

Supplement: Supplementary file 4 — Source data Fig. 1 [file 44321_2025_321_MOESM4_ESM.zip › Figure 1/1B/1B Small intestine E138A cropped scale bar.tif]

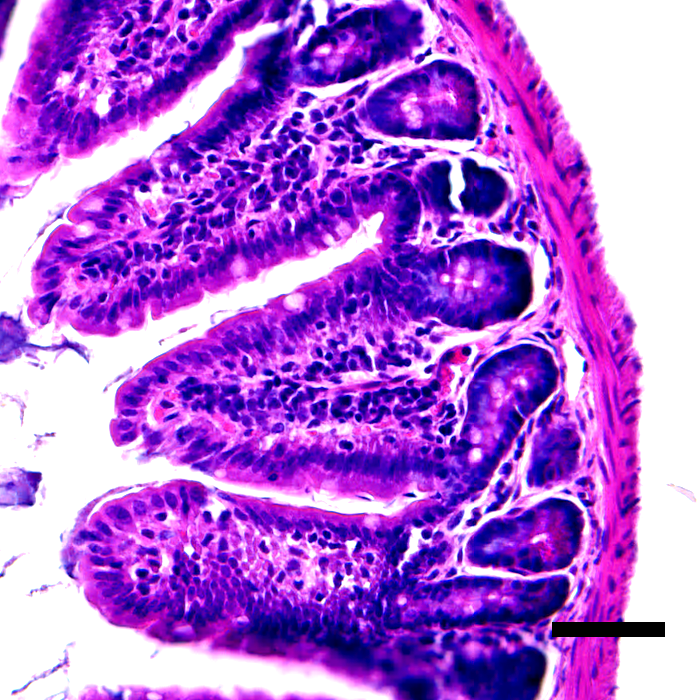

Supplement: Supplementary file 4 — Source data Fig. 1 [file 44321_2025_321_MOESM4_ESM.zip › Figure 1/1B/1B Small intestine WT cropped scale bar.tif]

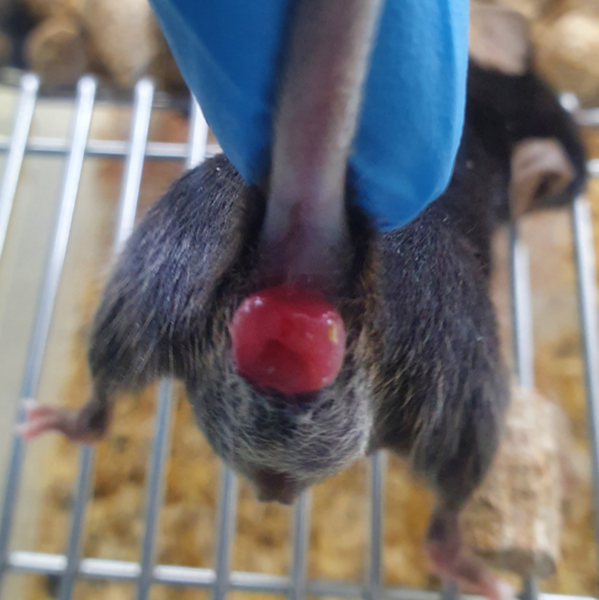

Supplement: Supplementary file 5 — Source data Fig. 2 [file 44321_2025_321_MOESM5_ESM.zip › Figure 2/2A/2A Prolapse image E138A cropped.png]

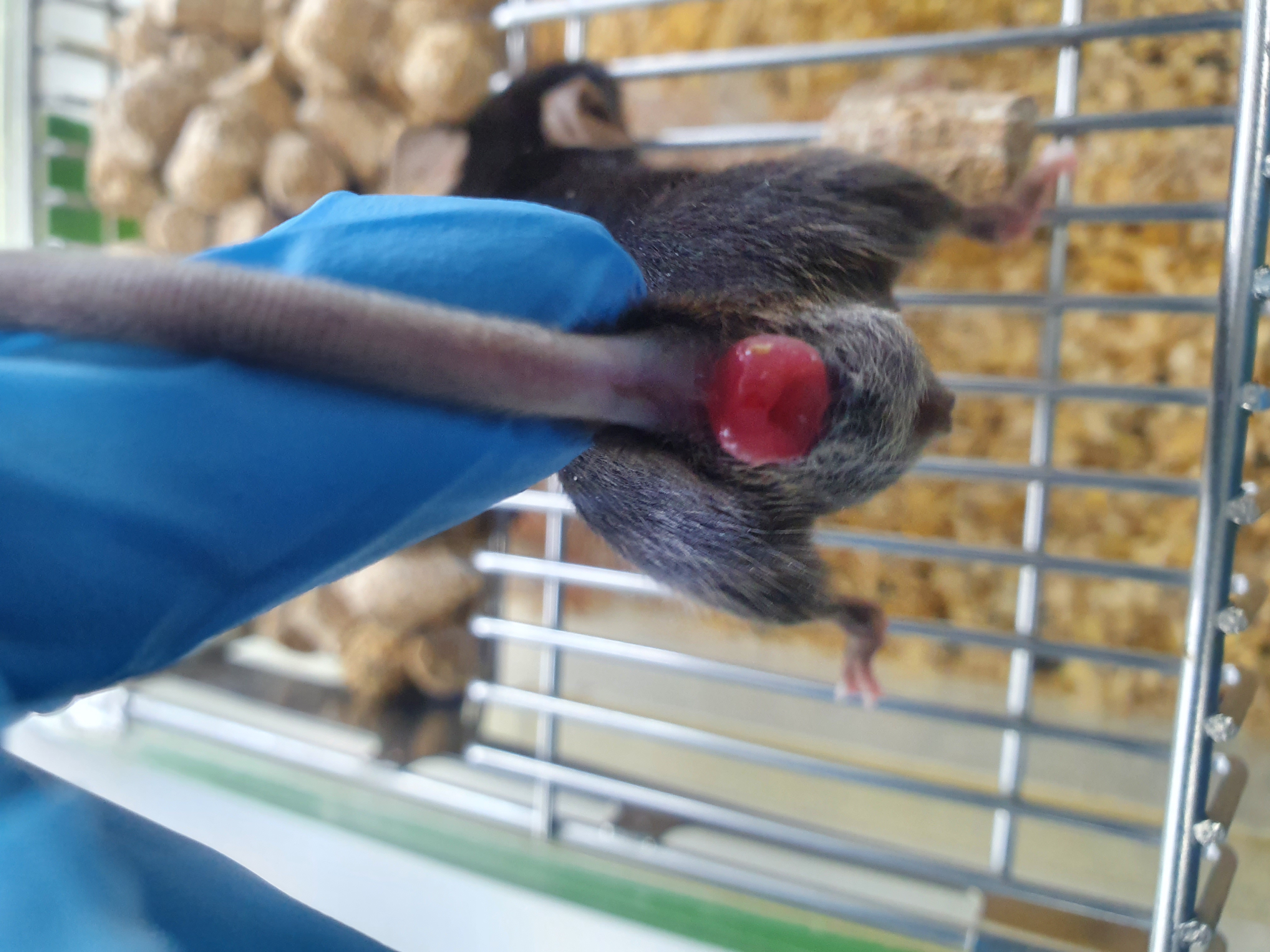

Supplement: Supplementary file 5 — Source data Fig. 2 [file 44321_2025_321_MOESM5_ESM.zip › Figure 2/2A/2A Prolapse image E138A original.jpg]

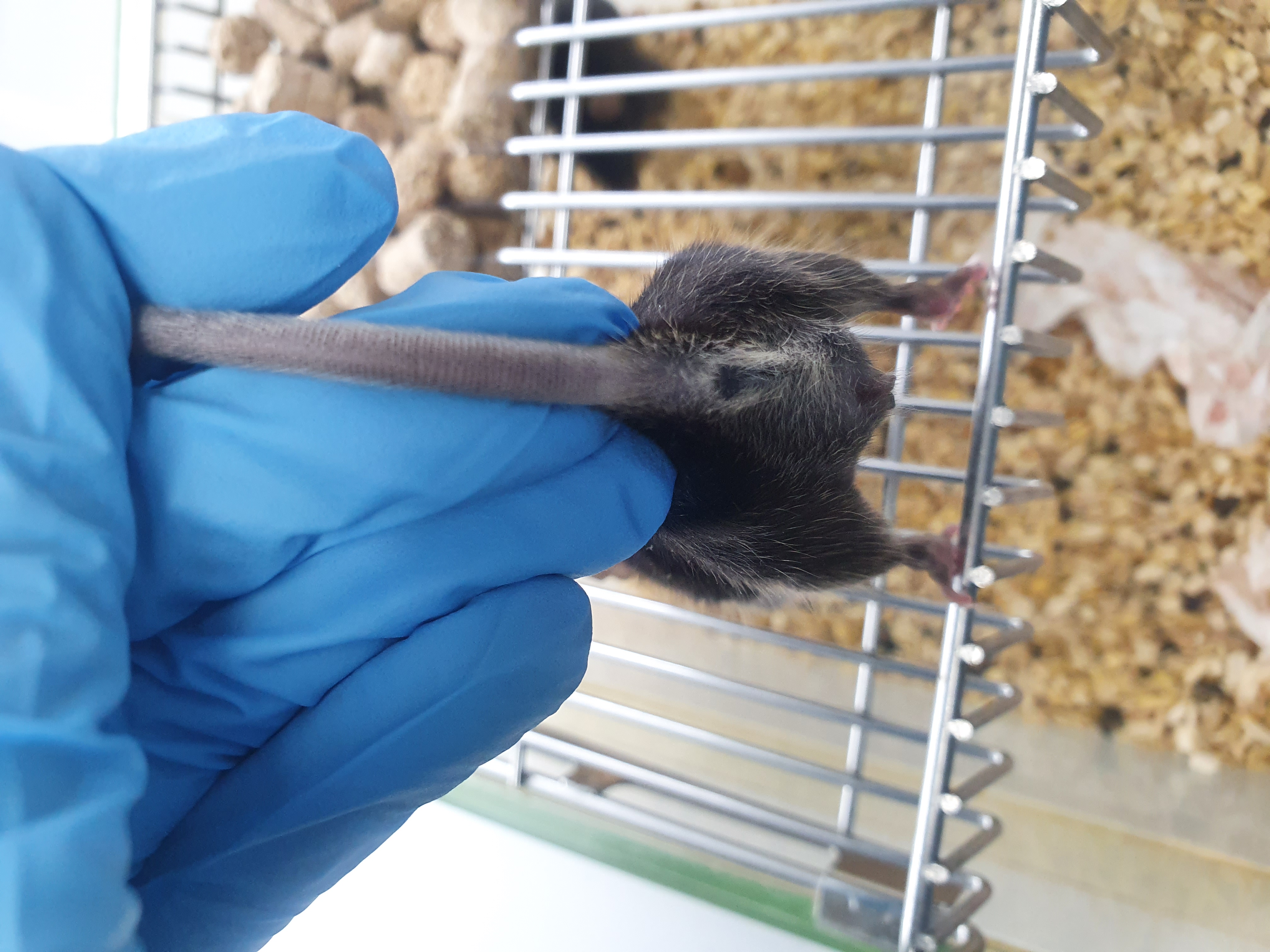

Supplement: Supplementary file 5 — Source data Fig. 2 [file 44321_2025_321_MOESM5_ESM.zip › Figure 2/2A/2A Prolapse Image WT original.jpg]

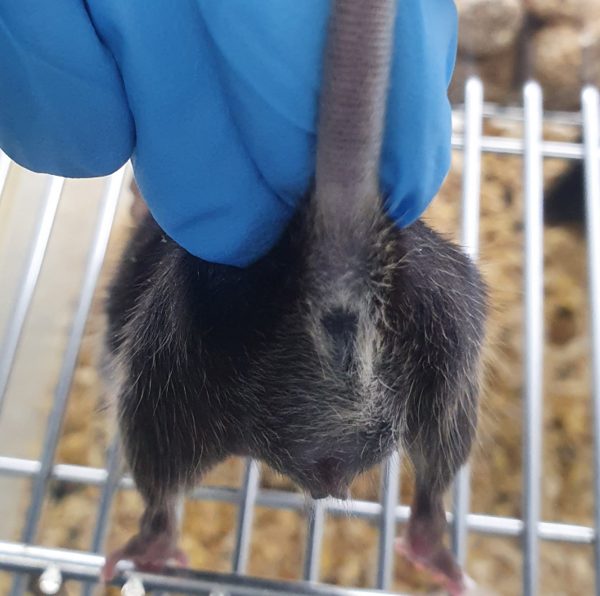

Supplement: Supplementary file 5 — Source data Fig. 2 [file 44321_2025_321_MOESM5_ESM.zip › Figure 2/2A/2A Prolapse image WTcropped.png]

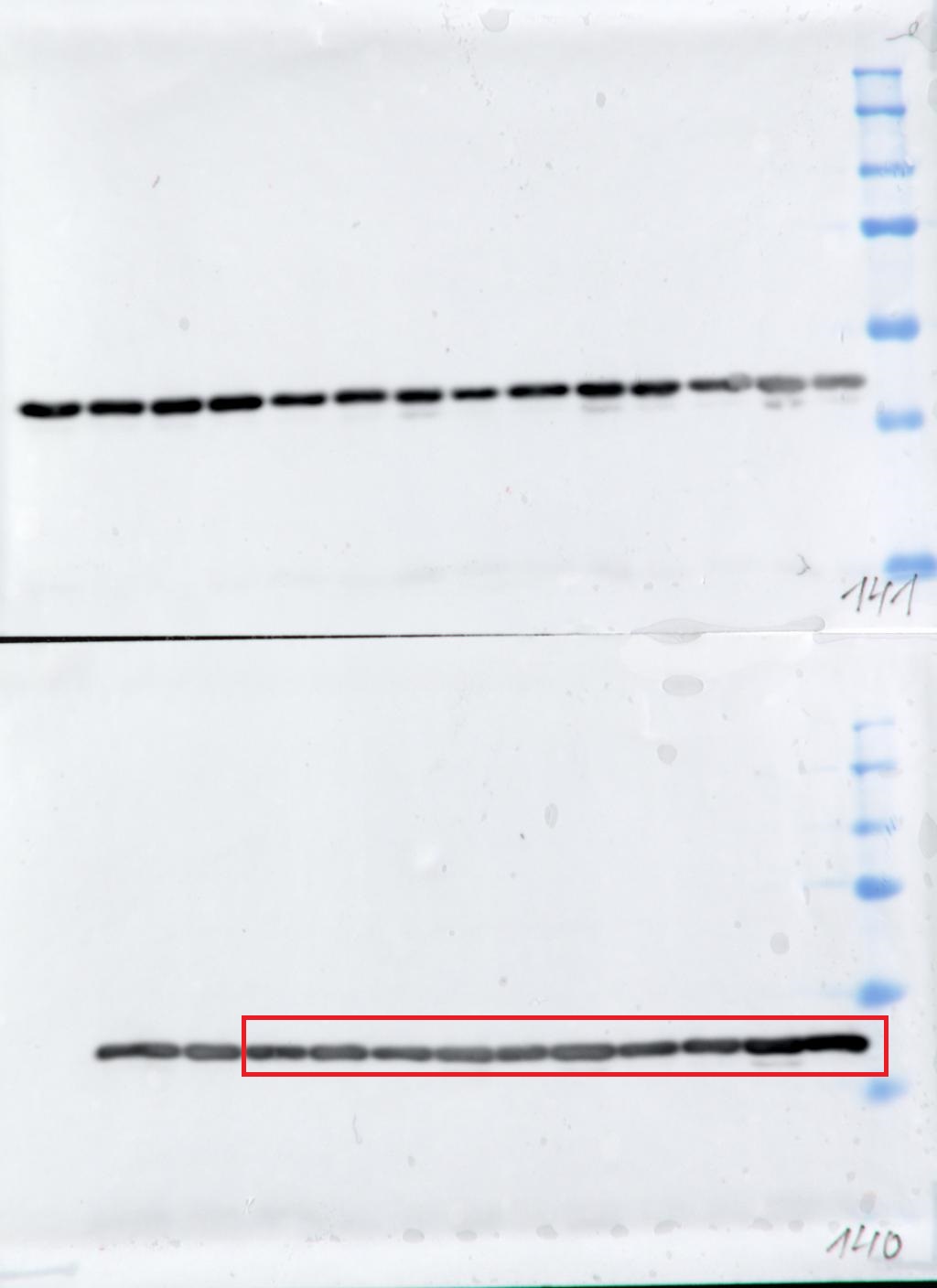

Supplement: Supplementary file 6 — Source data Fig. 3 [file 44321_2025_321_MOESM6_ESM.zip › Figure 3/3D/3D WB Actin original blot.jpg]

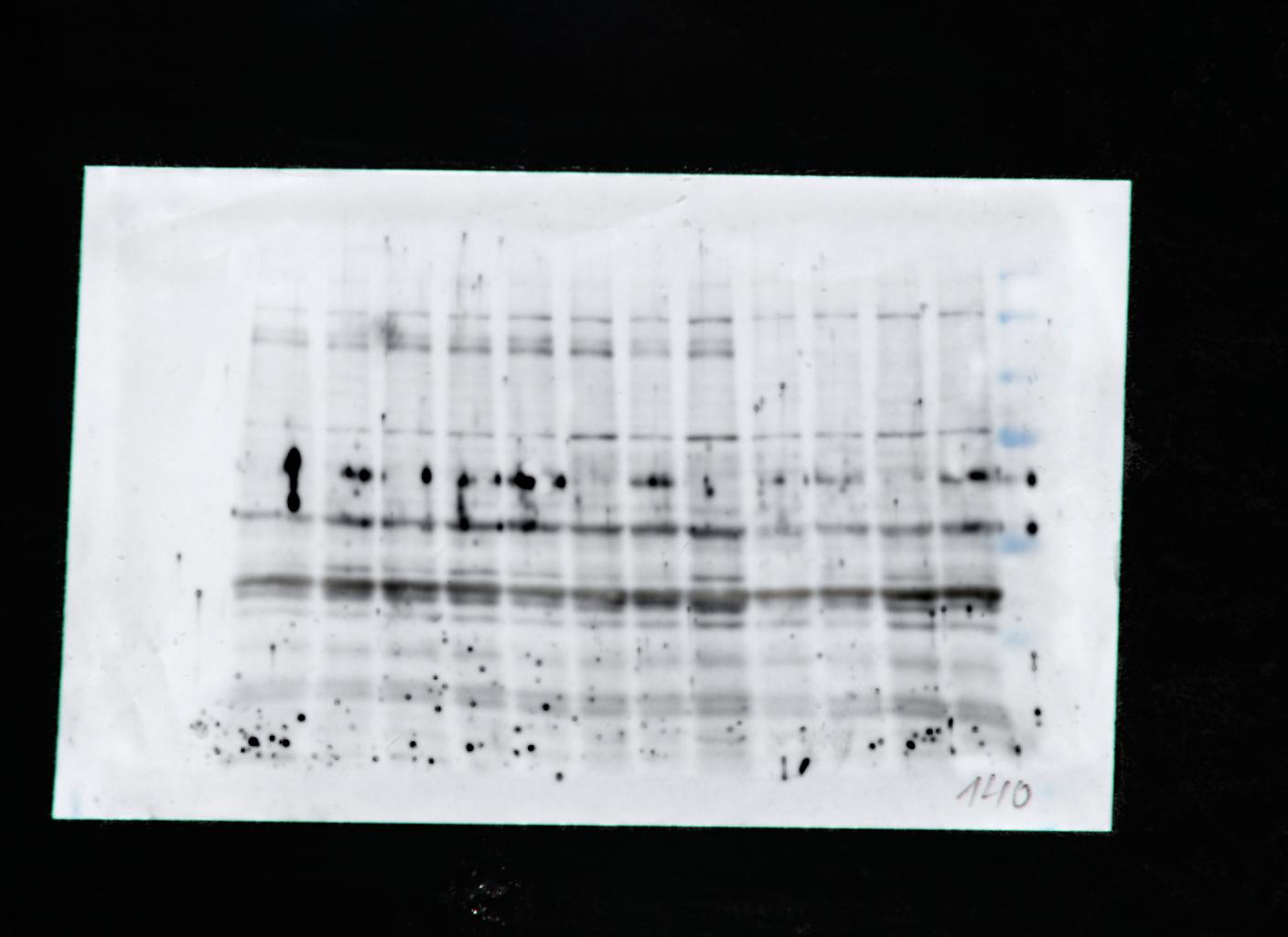

Supplement: Supplementary file 6 — Source data Fig. 3 [file 44321_2025_321_MOESM6_ESM.zip › Figure 3/3D/3D WB CARD original blot.jpg]

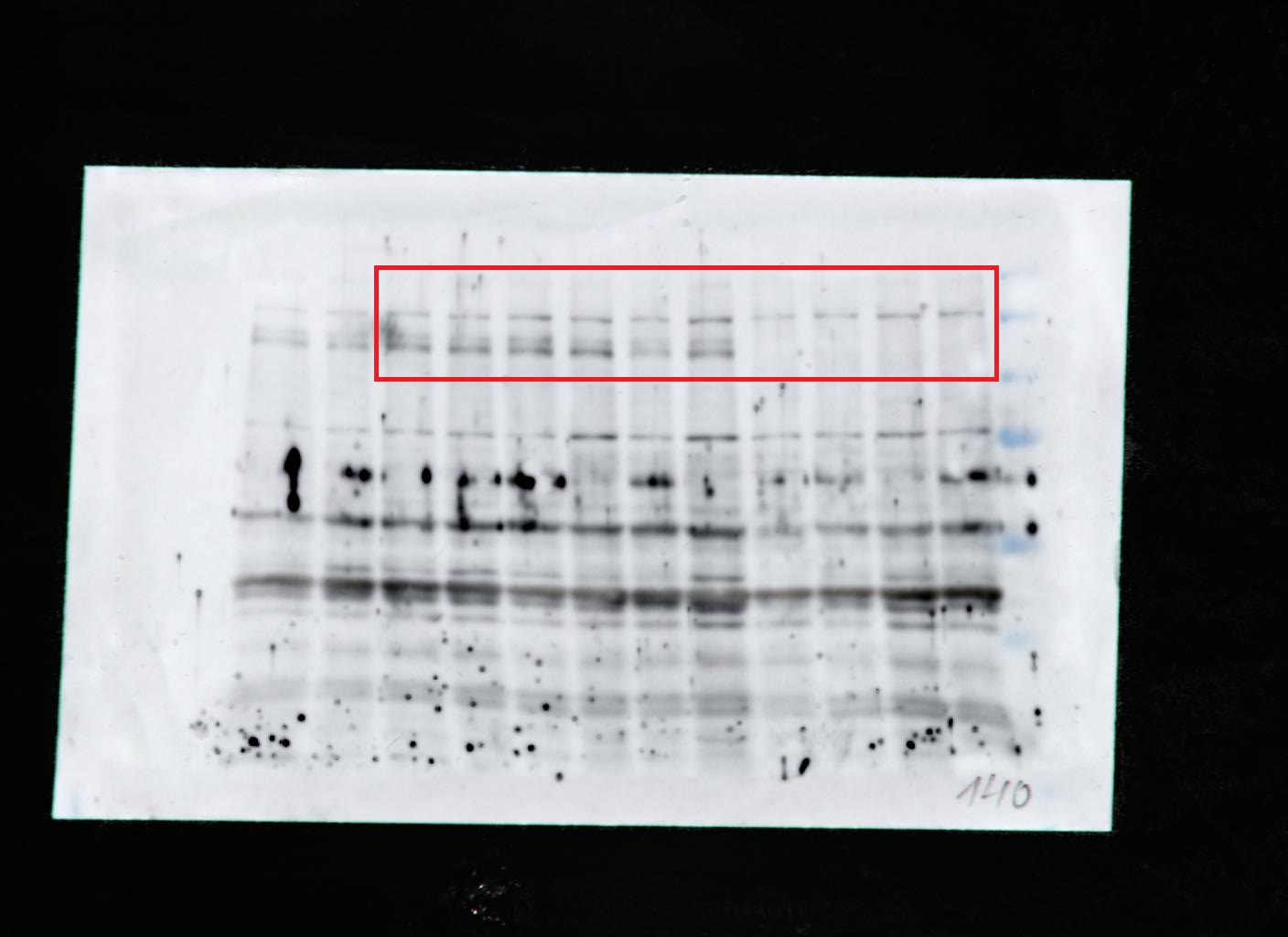

Supplement: Supplementary file 6 — Source data Fig. 3 [file 44321_2025_321_MOESM6_ESM.zip › Figure 3/3D/3D WB CARD14 original blot.jpg]

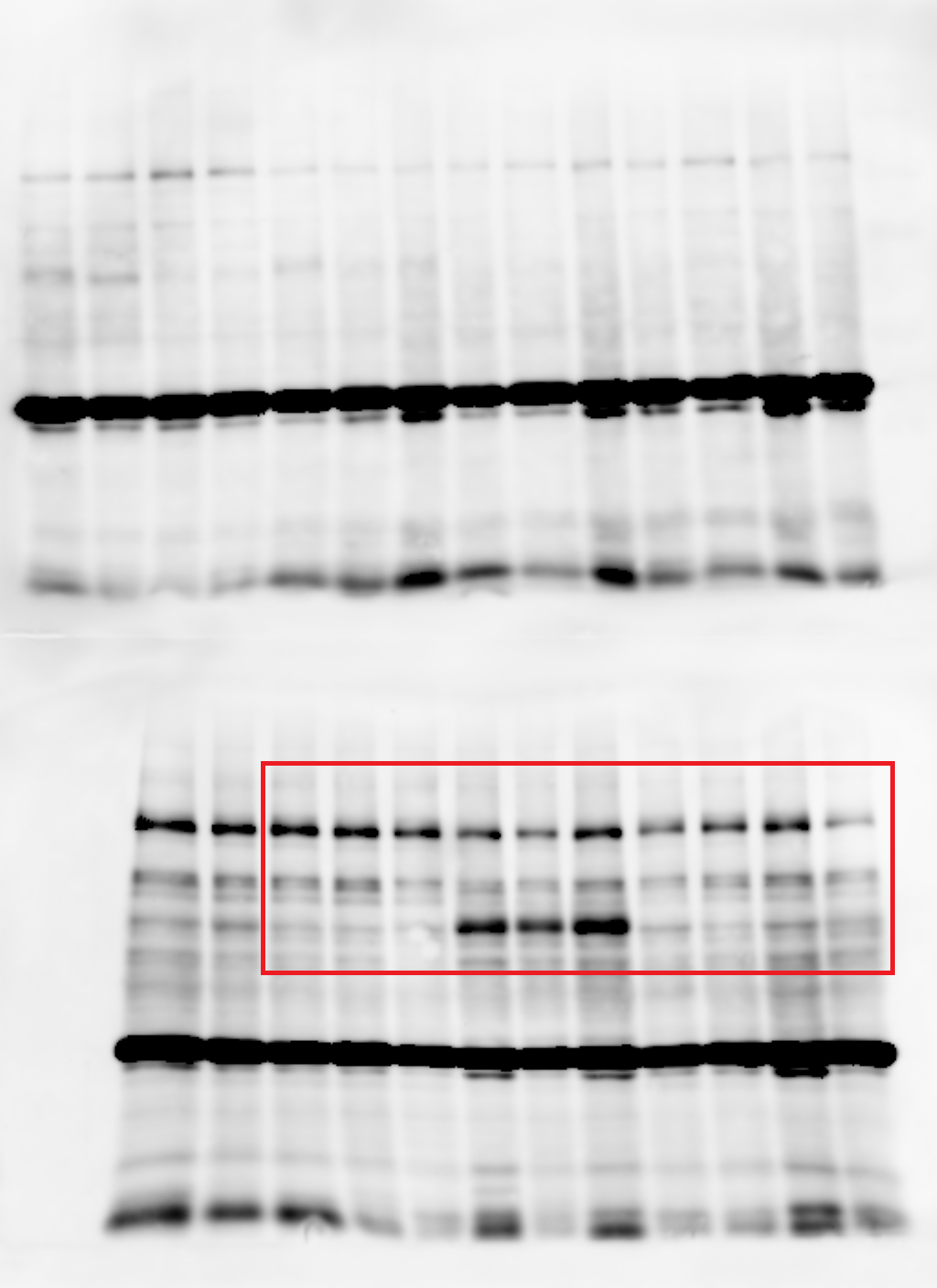

Supplement: Supplementary file 6 — Source data Fig. 3 [file 44321_2025_321_MOESM6_ESM.zip › Figure 3/3D/3D WB CYLD original blot.tif]

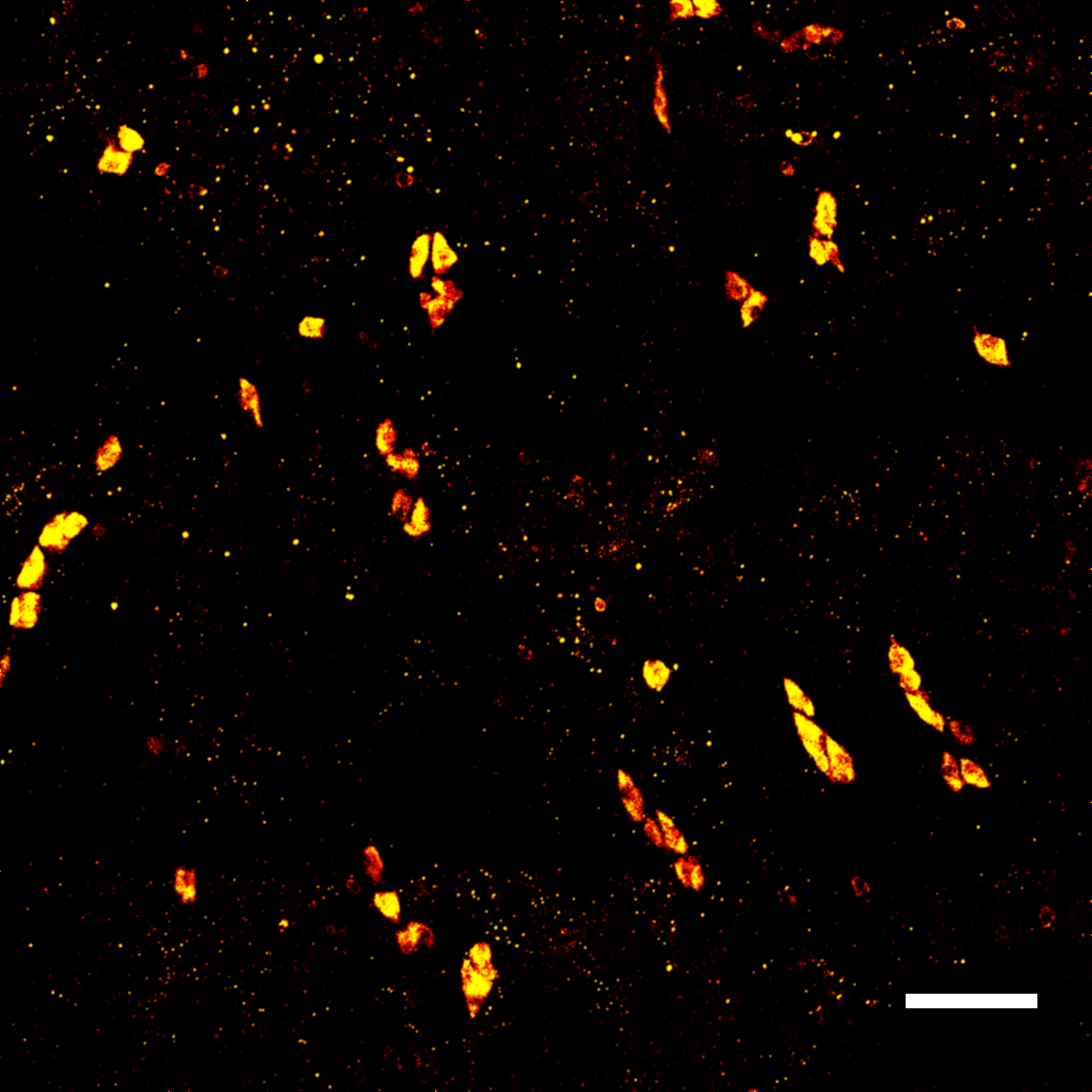

Supplement: Supplementary file 7 — Source data Fig. 4 [file 44321_2025_321_MOESM7_ESM.zip › Figure 4/4C/4C HucCD E138A modified.png]

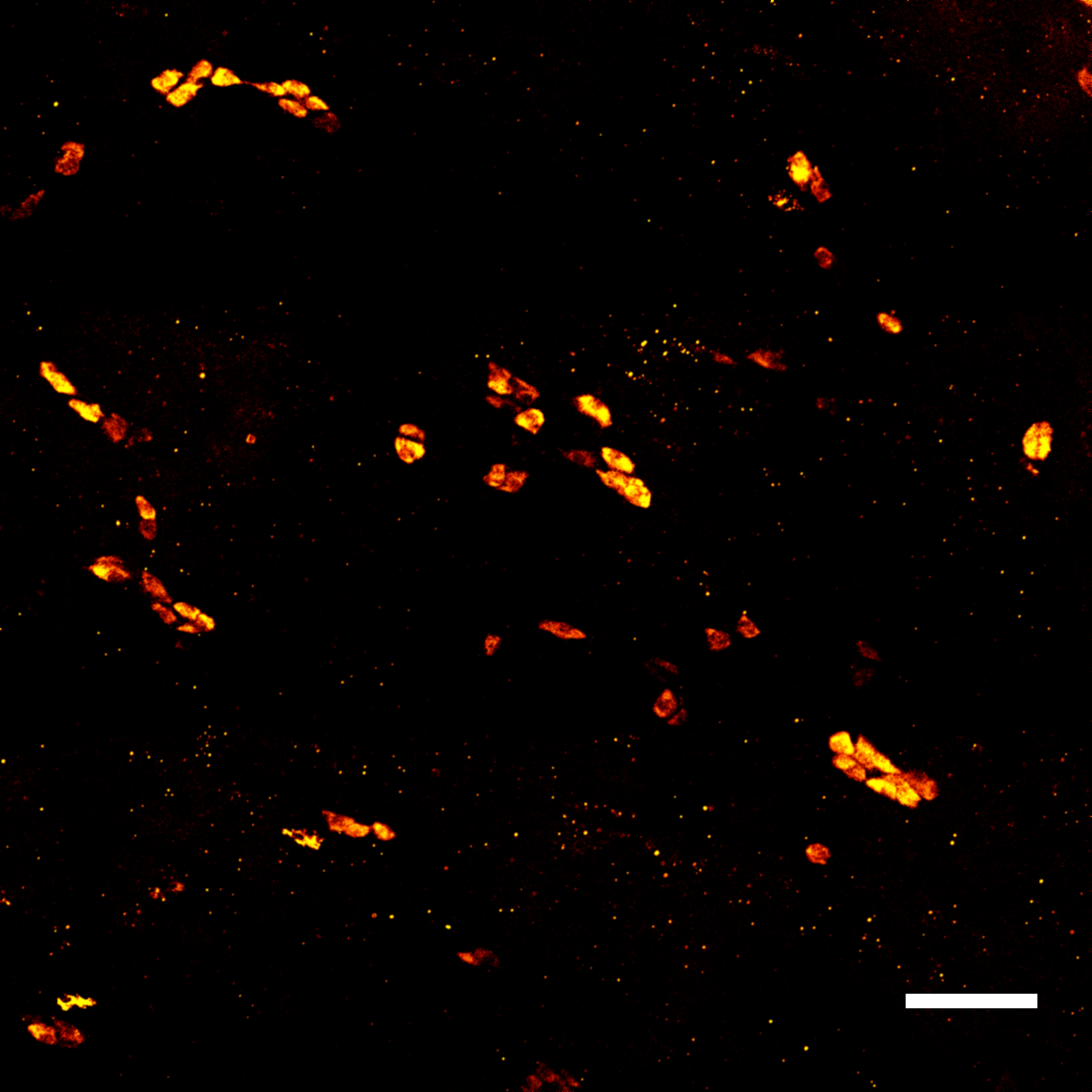

Supplement: Supplementary file 7 — Source data Fig. 4 [file 44321_2025_321_MOESM7_ESM.zip › Figure 4/4C/4C HucCD WT modified.png]

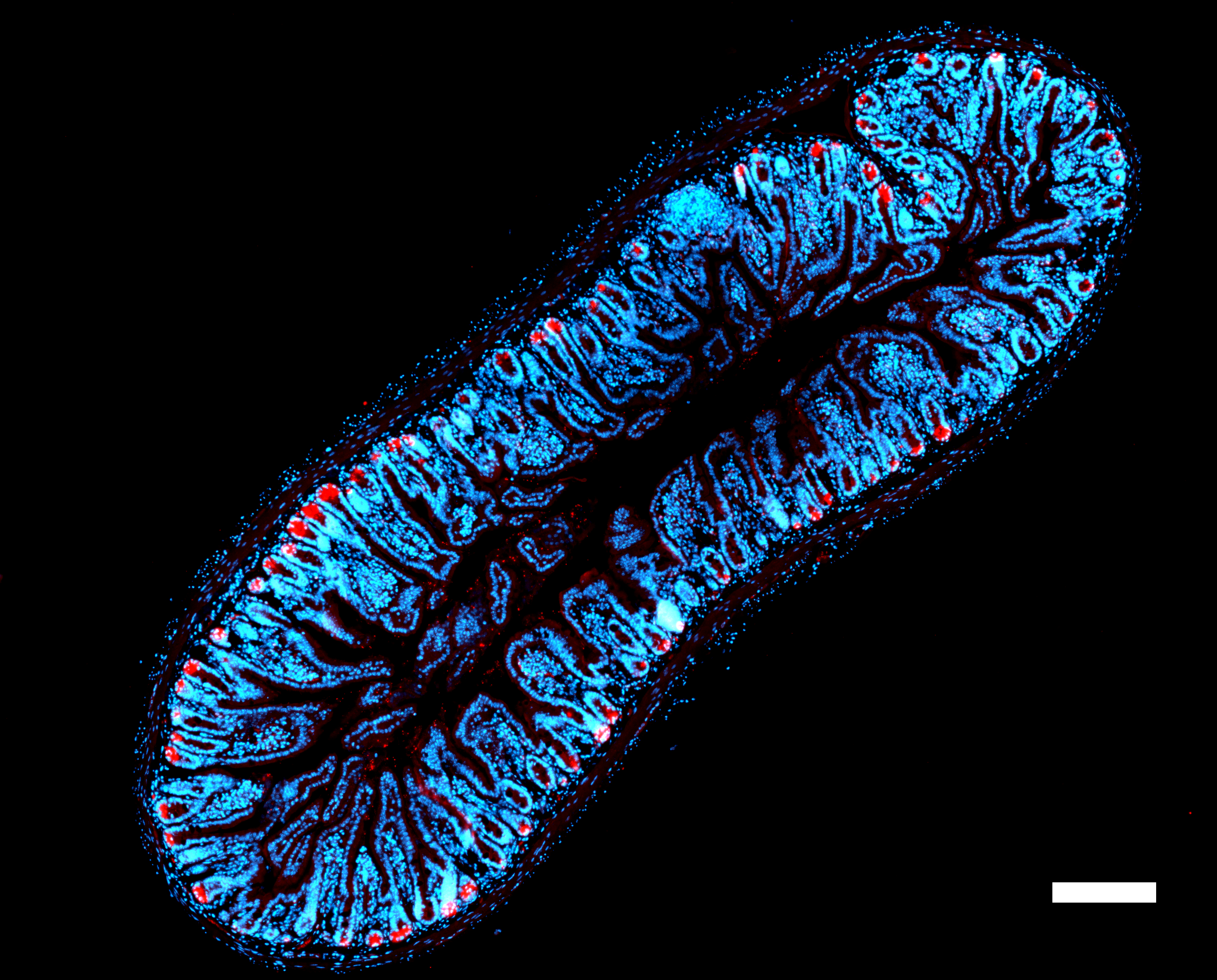

Supplement: Supplementary file 9 — Source data Fig. 6 [file 44321_2025_321_MOESM9_ESM.zip › Figure 6/6A/6A Lysozyme E138A modified.tif]

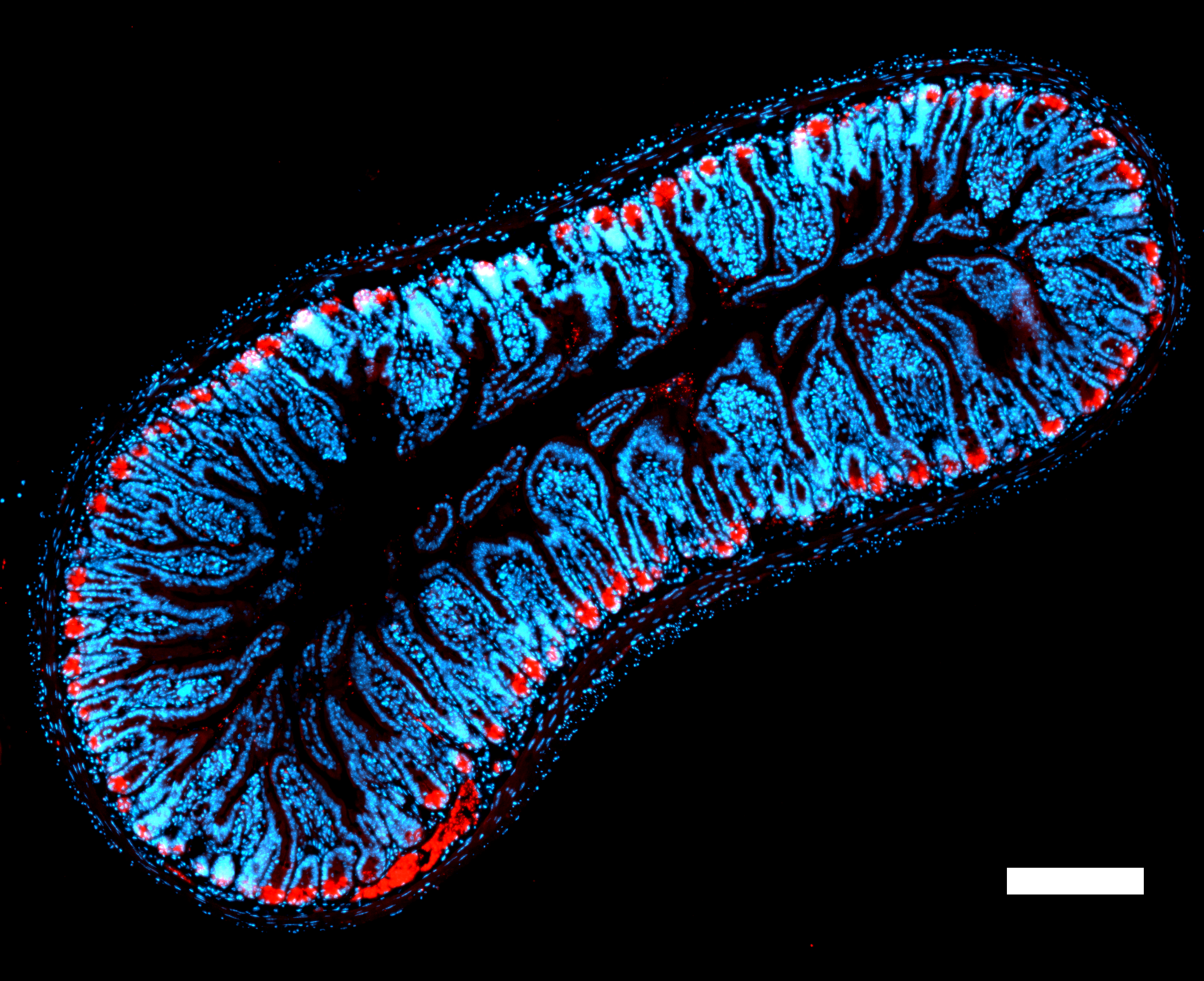

Supplement: Supplementary file 9 — Source data Fig. 6 [file 44321_2025_321_MOESM9_ESM.zip › Figure 6/6A/6A Lysozyme WT modified.tif]

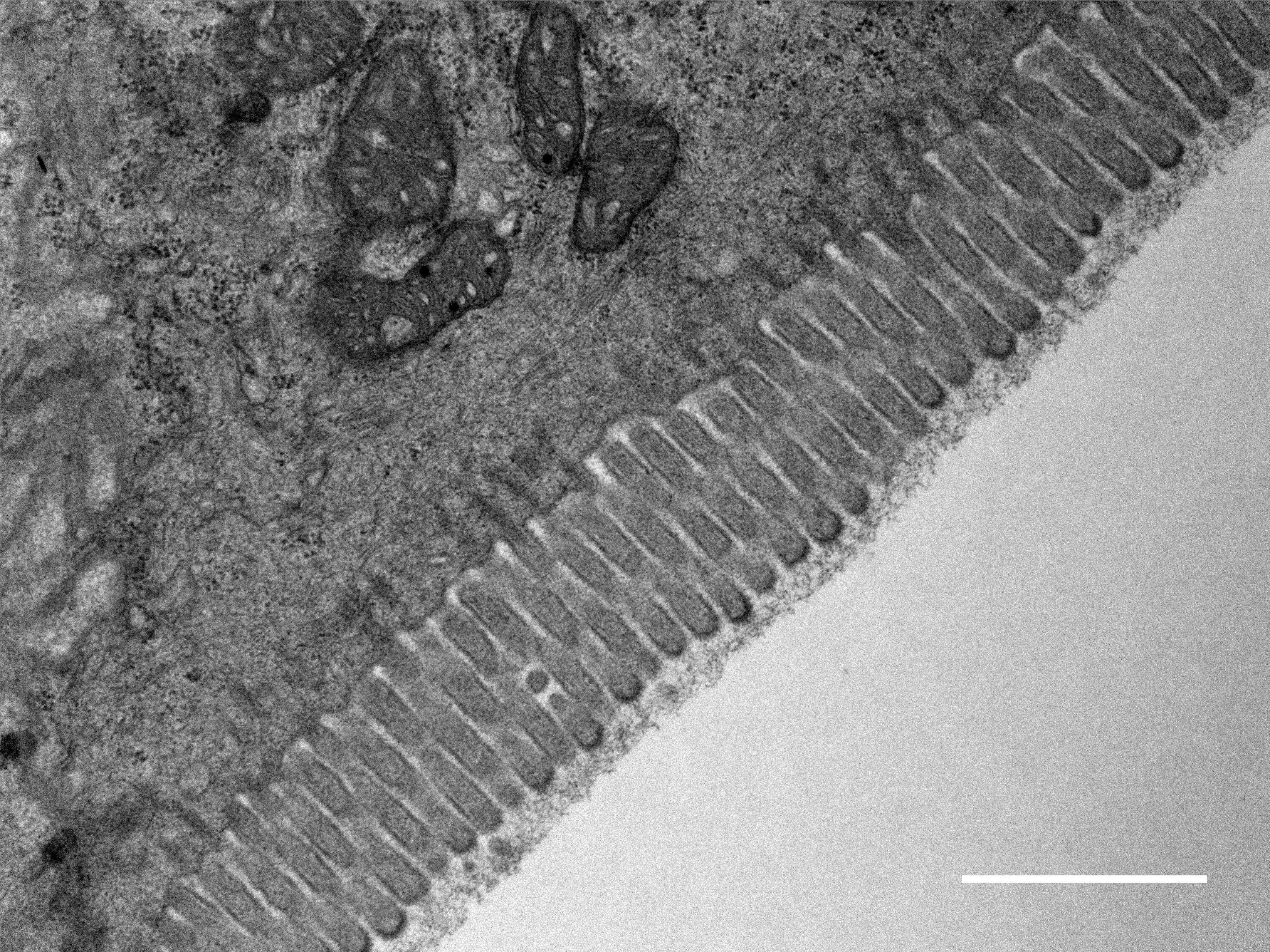

Supplement: Supplementary file 9 — Source data Fig. 6 [file 44321_2025_321_MOESM9_ESM.zip › Figure 6/6D/6D TEM E138A Brush Border.tif]

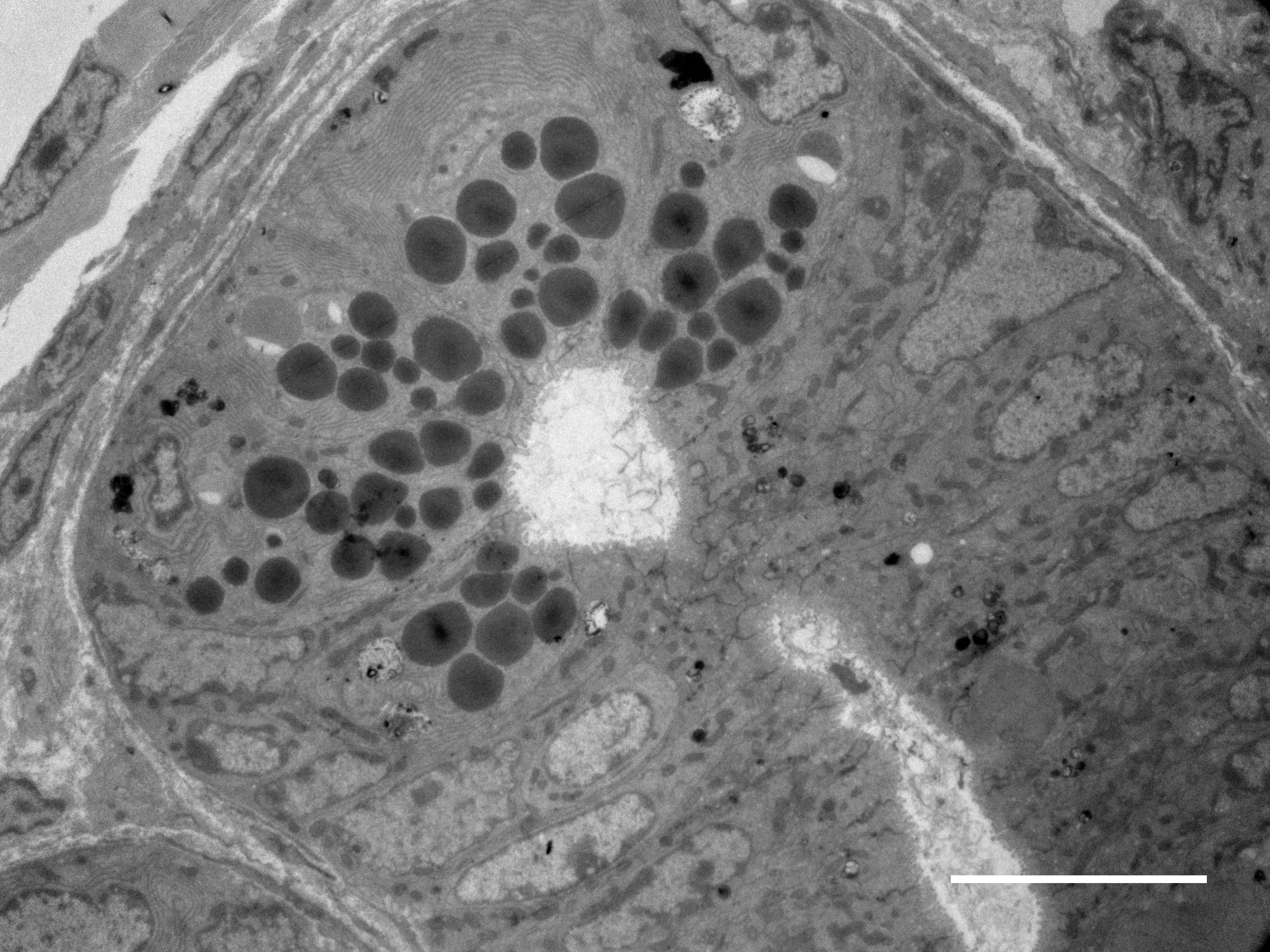

Supplement: Supplementary file 9 — Source data Fig. 6 [file 44321_2025_321_MOESM9_ESM.zip › Figure 6/6D/6D TEM E138A Crypt Paneth cells.tif]

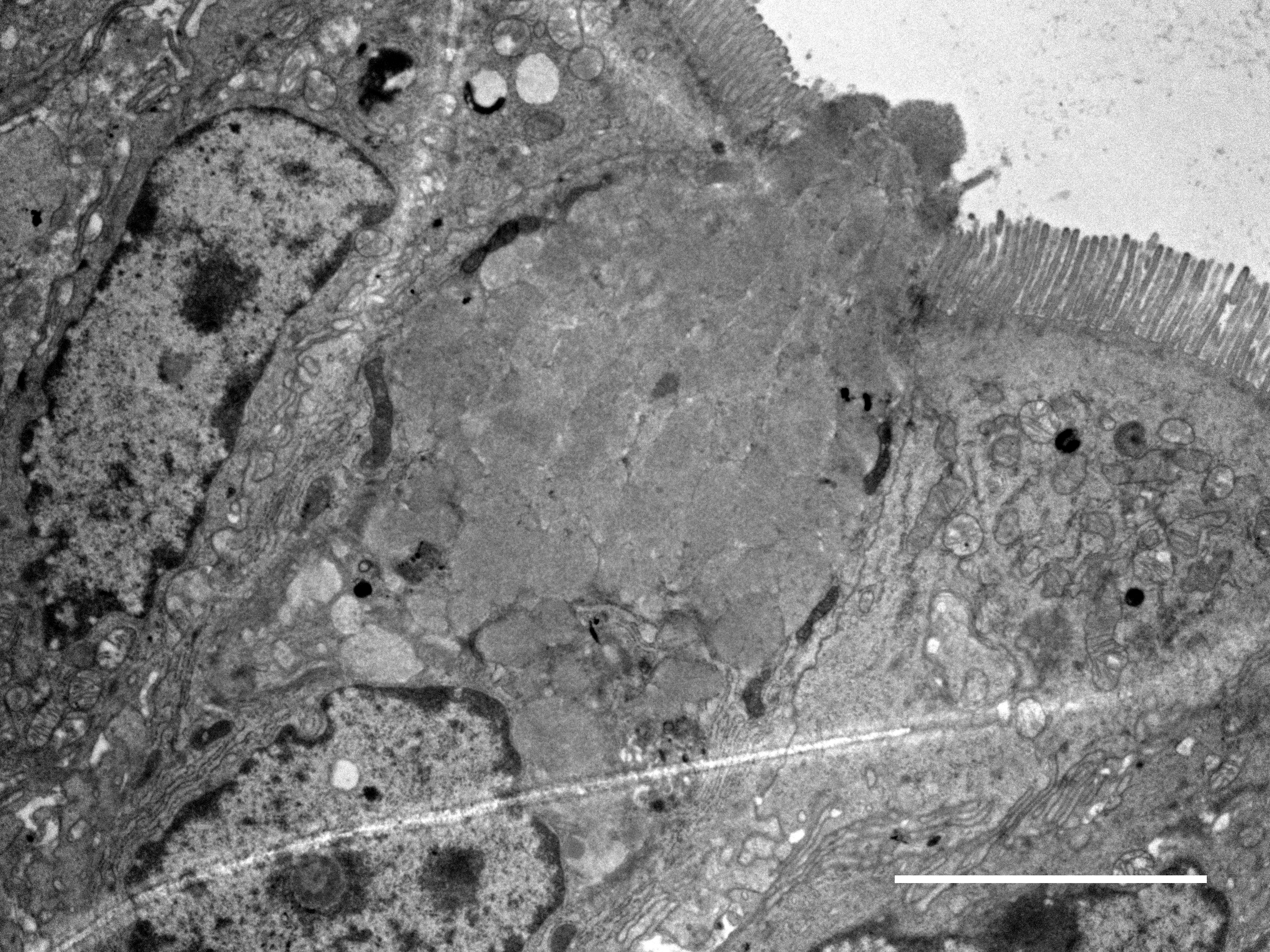

Supplement: Supplementary file 9 — Source data Fig. 6 [file 44321_2025_321_MOESM9_ESM.zip › Figure 6/6D/6D TEM E138A Goblet cells.tif]

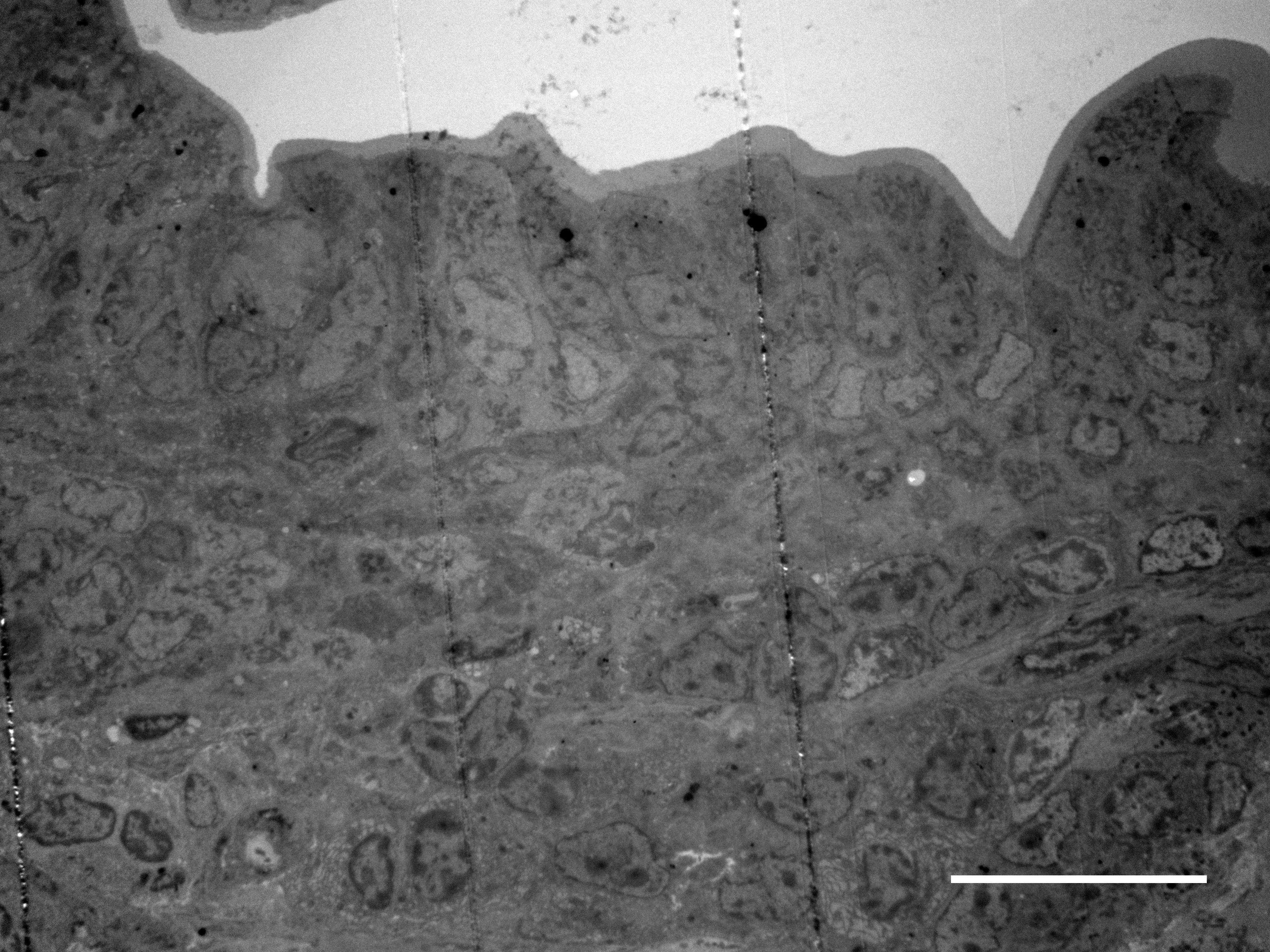

Supplement: Supplementary file 9 — Source data Fig. 6 [file 44321_2025_321_MOESM9_ESM.zip › Figure 6/6D/6D TEM E138A Villus Enterocytes.tif]

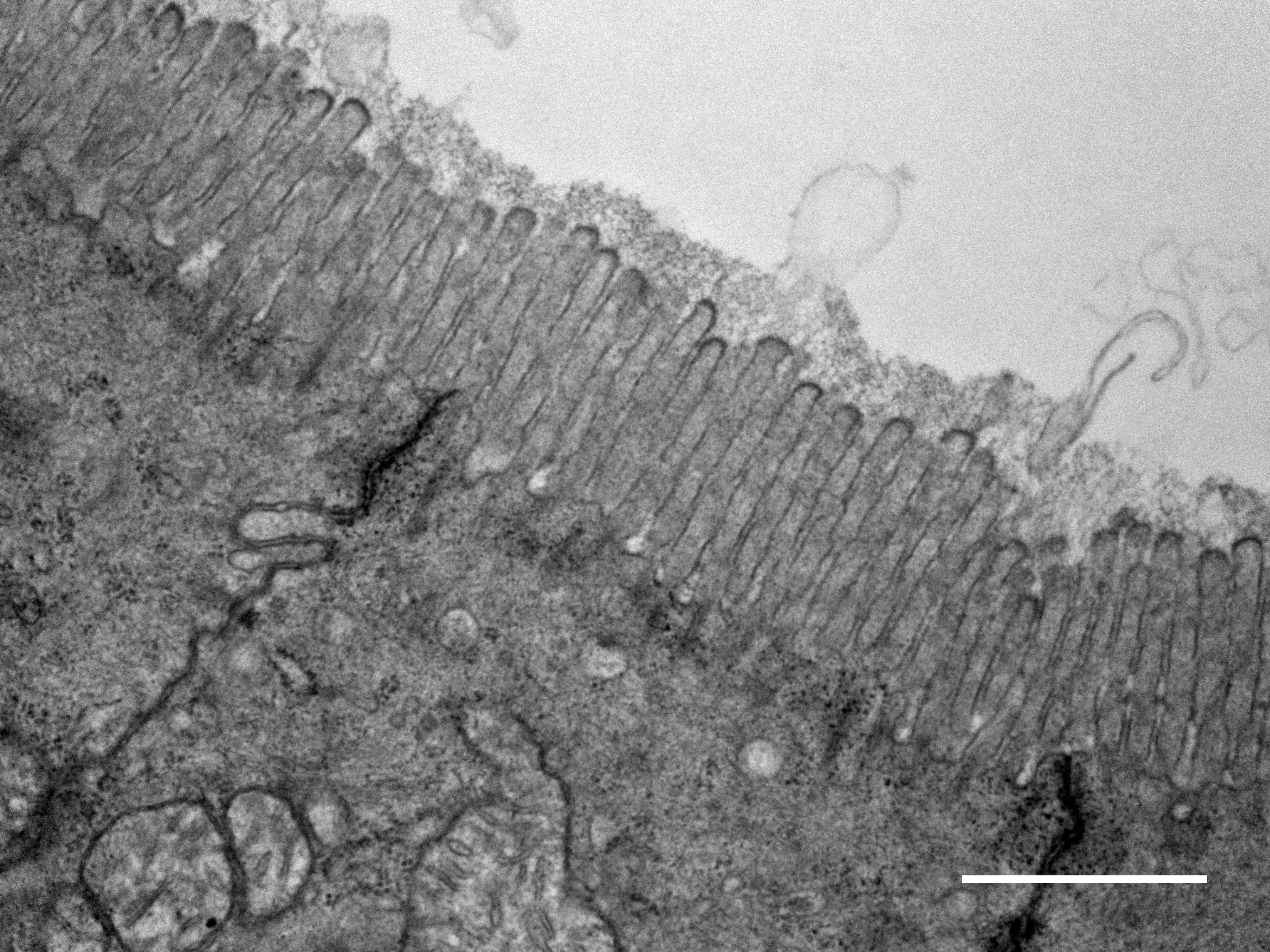

Supplement: Supplementary file 9 — Source data Fig. 6 [file 44321_2025_321_MOESM9_ESM.zip › Figure 6/6D/6D TEM WT Brush Border.tif]

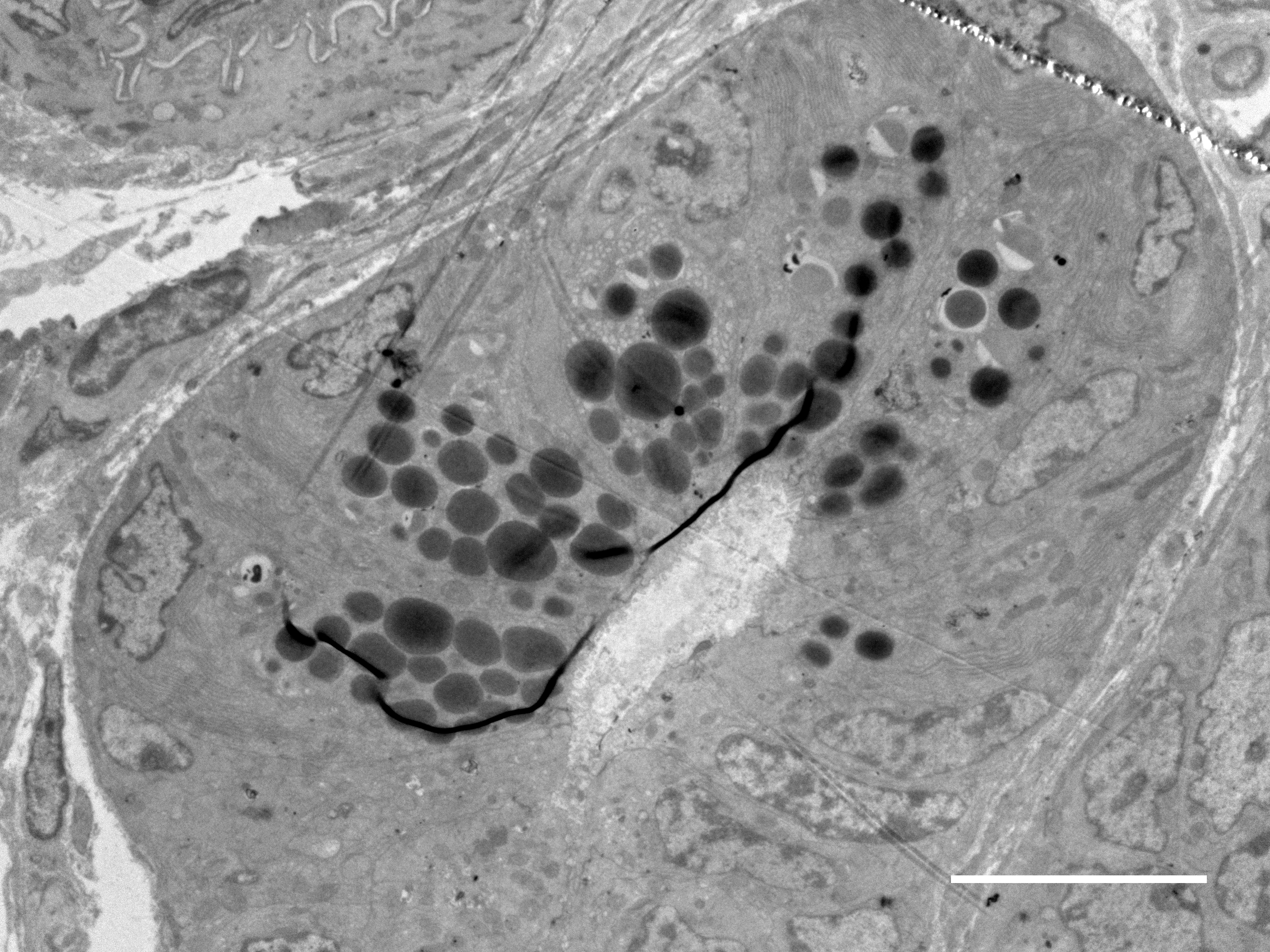

Supplement: Supplementary file 9 — Source data Fig. 6 [file 44321_2025_321_MOESM9_ESM.zip › Figure 6/6D/6D TEM WT Crypt Paneth cells.tif]

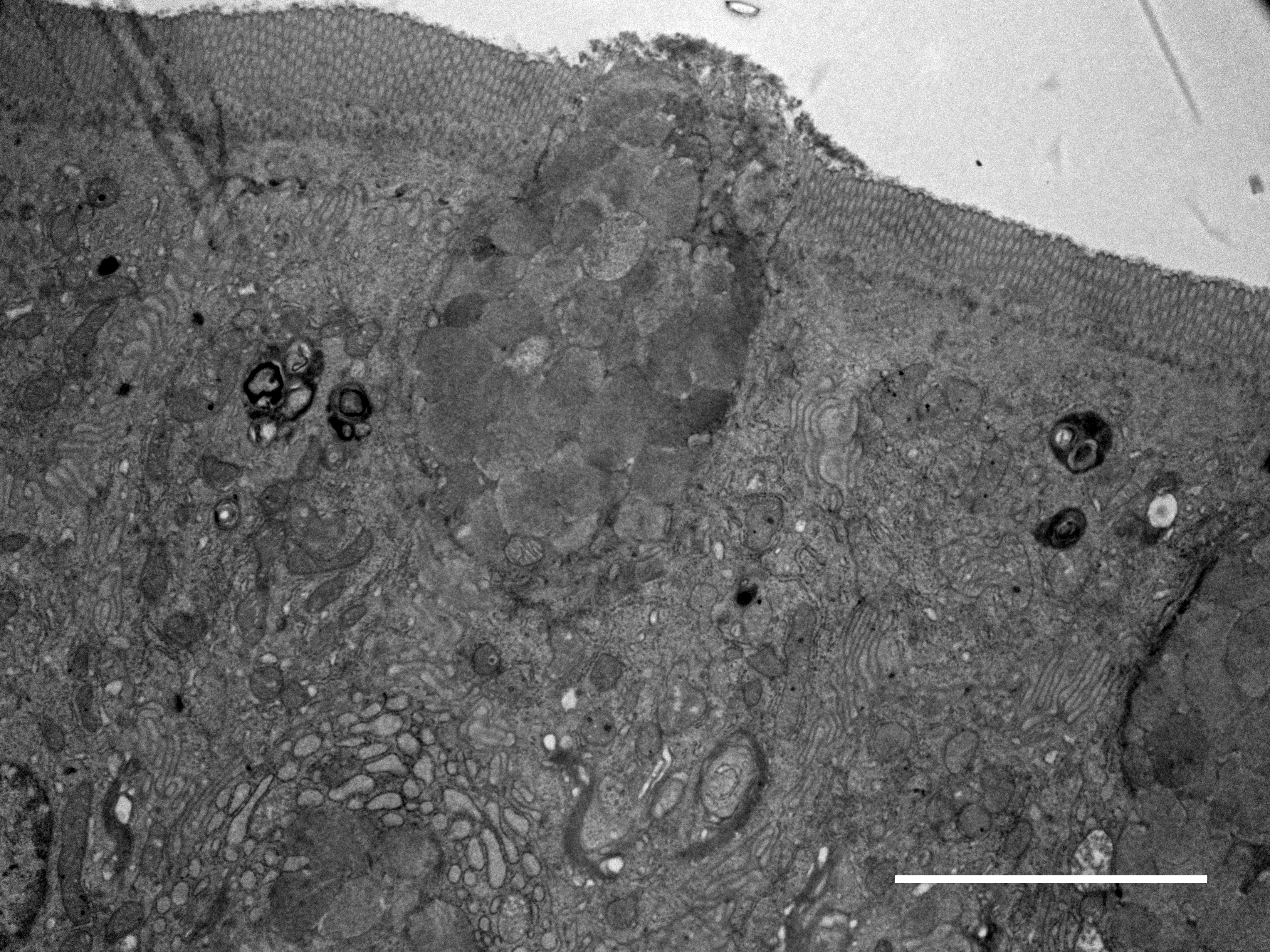

Supplement: Supplementary file 9 — Source data Fig. 6 [file 44321_2025_321_MOESM9_ESM.zip › Figure 6/6D/6D TEM WT Goblet cells.tif]

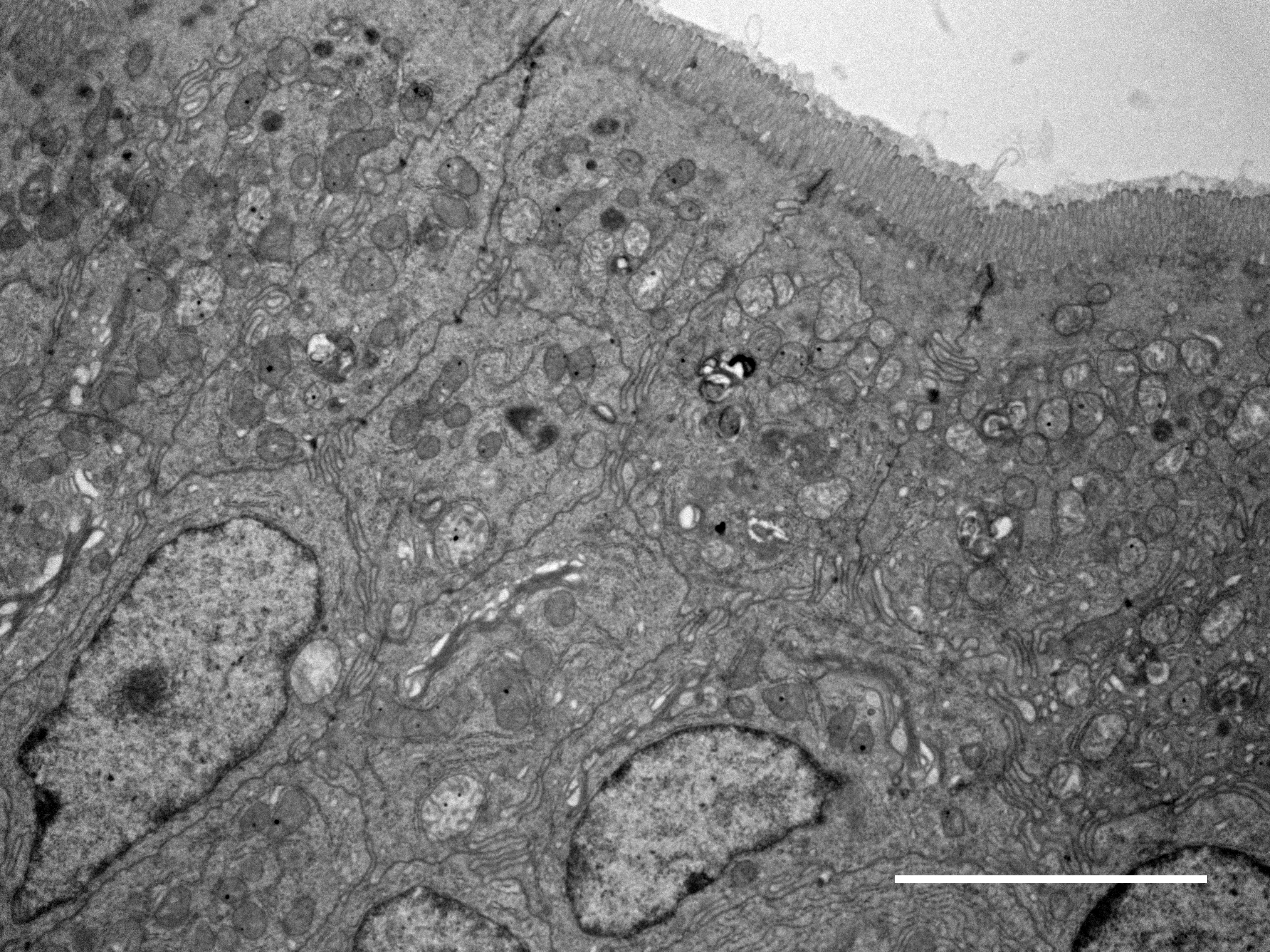

Supplement: Supplementary file 9 — Source data Fig. 6 [file 44321_2025_321_MOESM9_ESM.zip › Figure 6/6D/6D TEM WT Villus Enterocytes.tif]

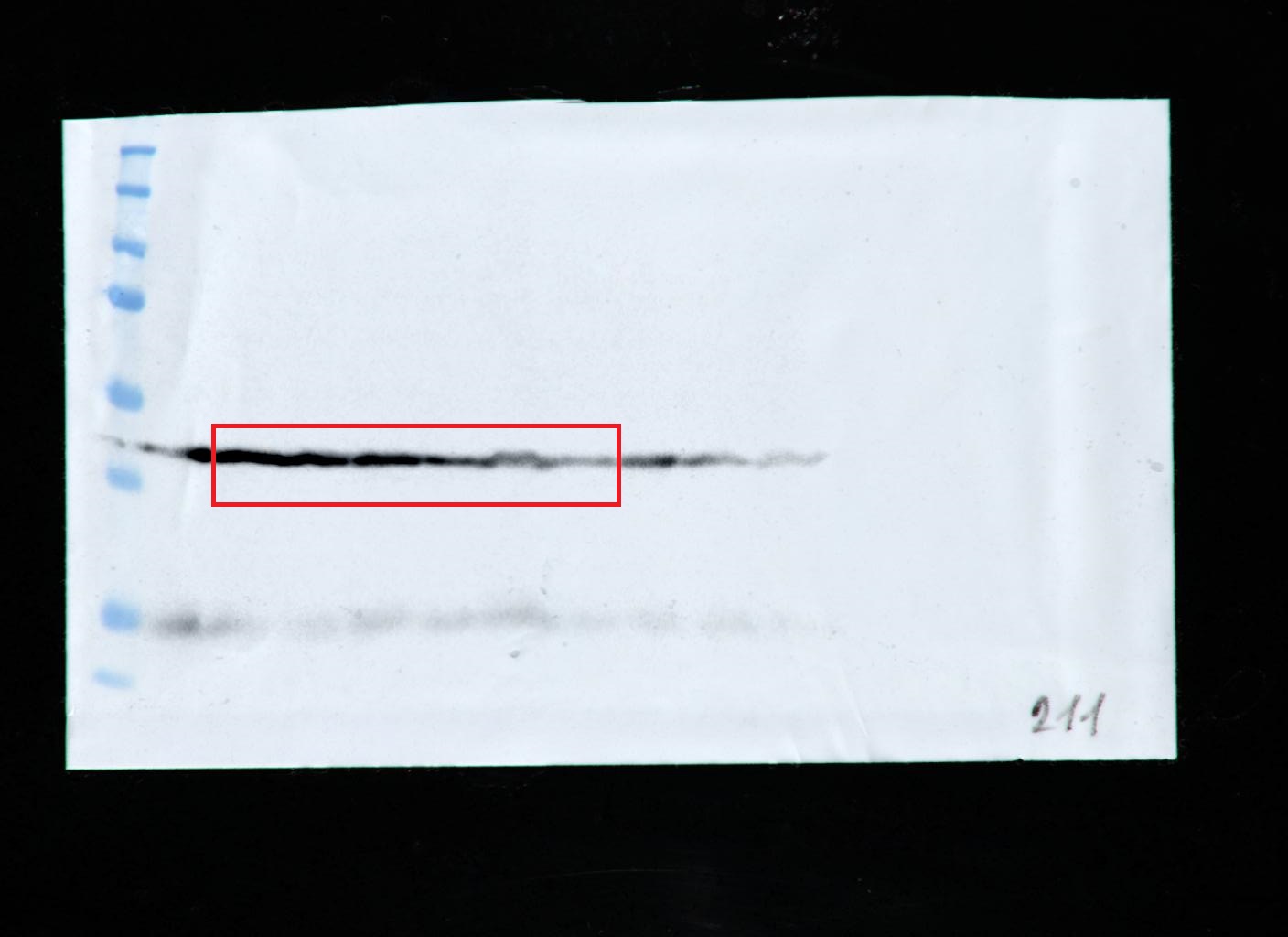

Supplement: Supplementary file 9 — Source data Fig. 6 [file 44321_2025_321_MOESM9_ESM.zip › Figure 6/6F/6F WB Actin blot original.jpg]

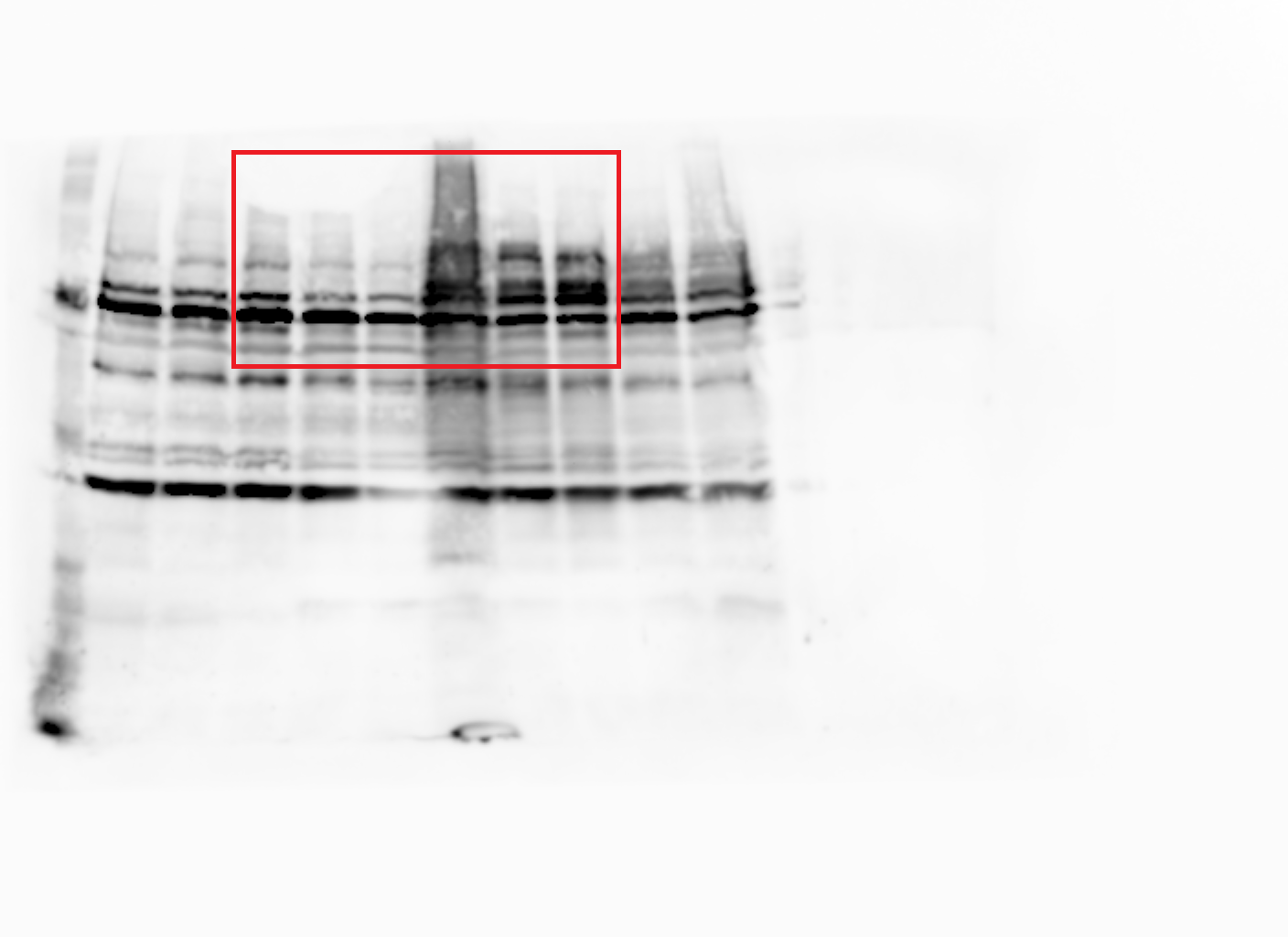

Supplement: Supplementary file 9 — Source data Fig. 6 [file 44321_2025_321_MOESM9_ESM.zip › Figure 6/6F/6F WB CARD14 blot original.tif]

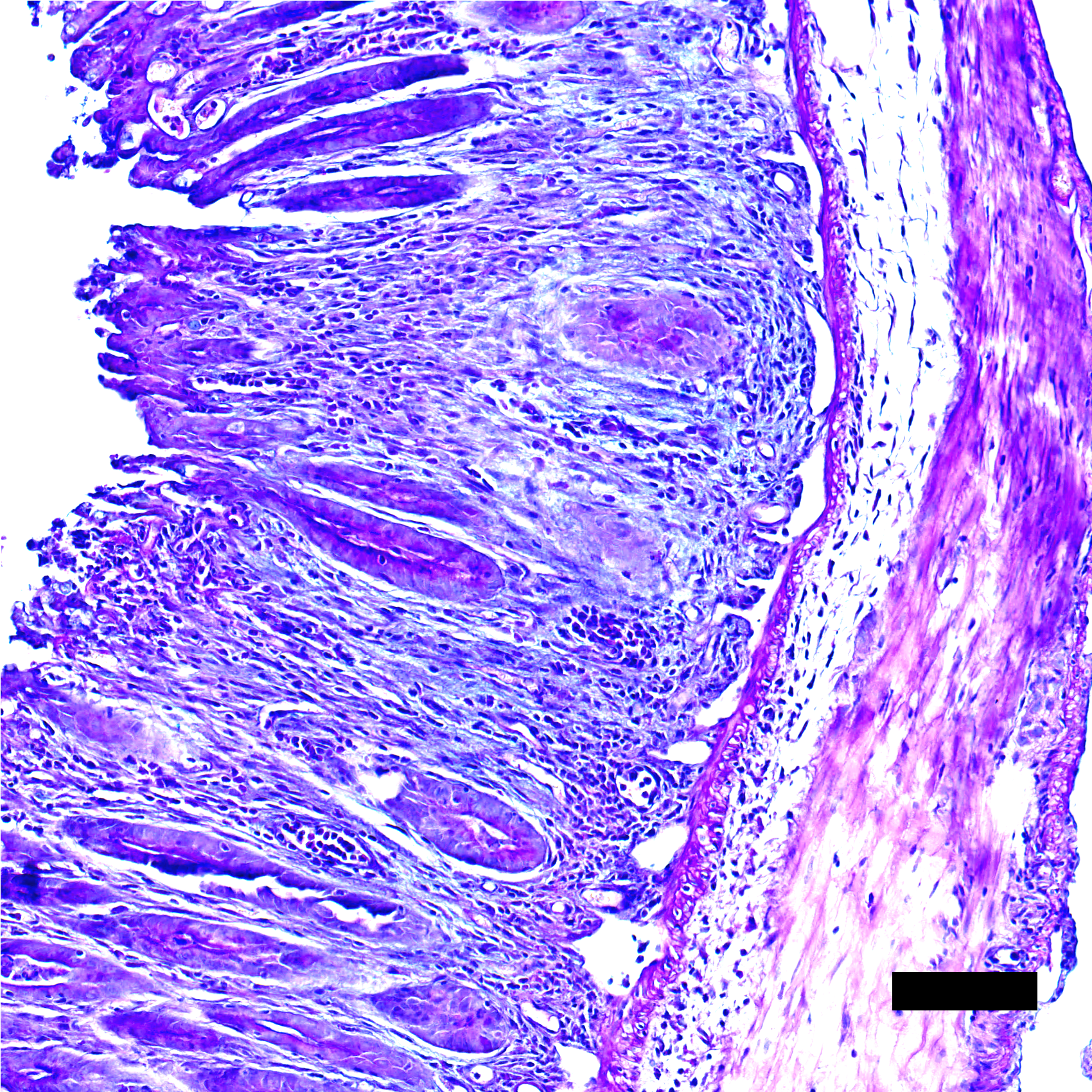

Supplement: Supplementary file 10 — Source data Fig. 7 [file 44321_2025_321_MOESM10_ESM.zip › Figure 7/7F/7F AB PAS E138A modified.png]

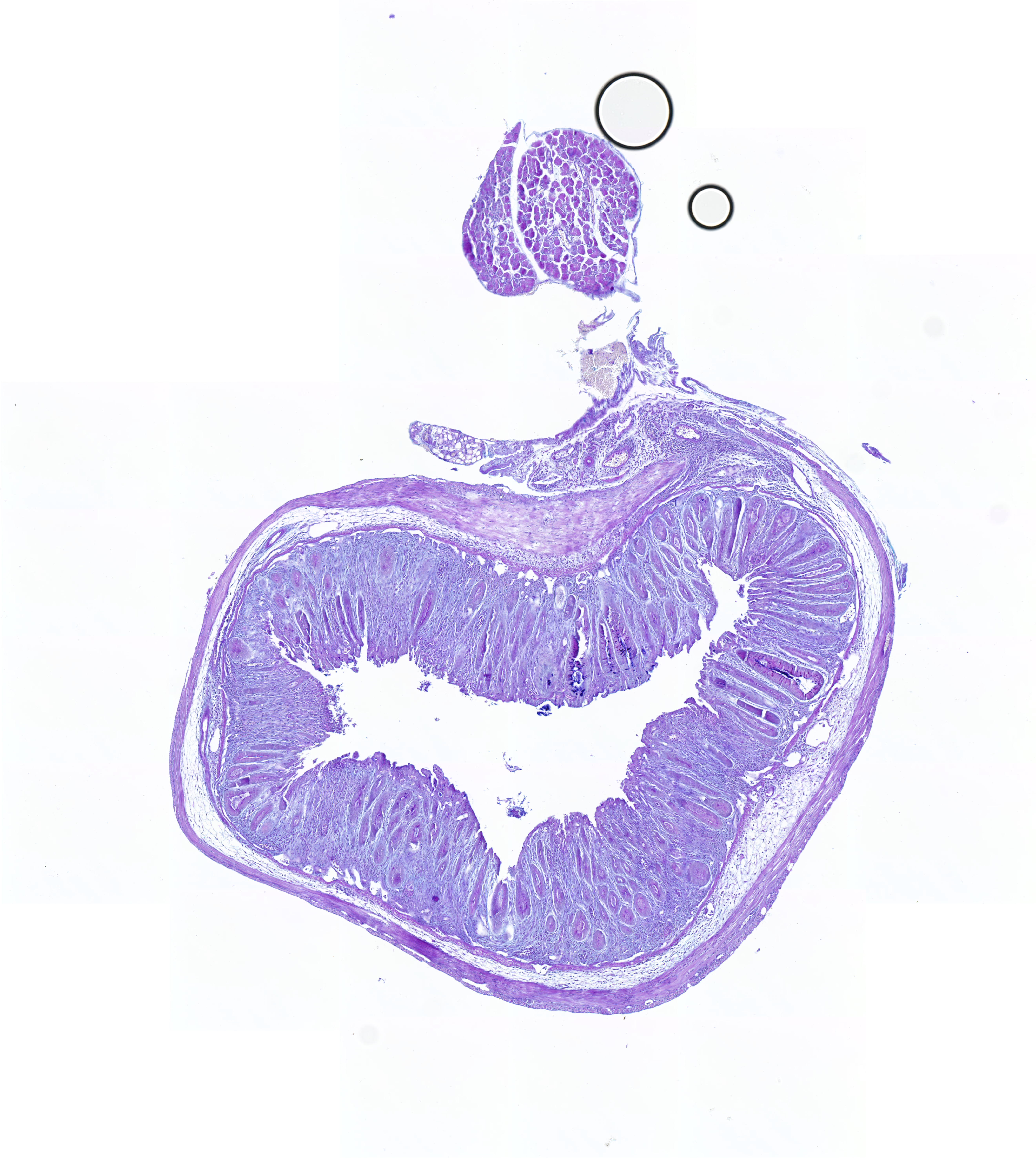

Supplement: Supplementary file 10 — Source data Fig. 7 [file 44321_2025_321_MOESM10_ESM.zip › Figure 7/7F/7F AB PAS E138A original.tif]

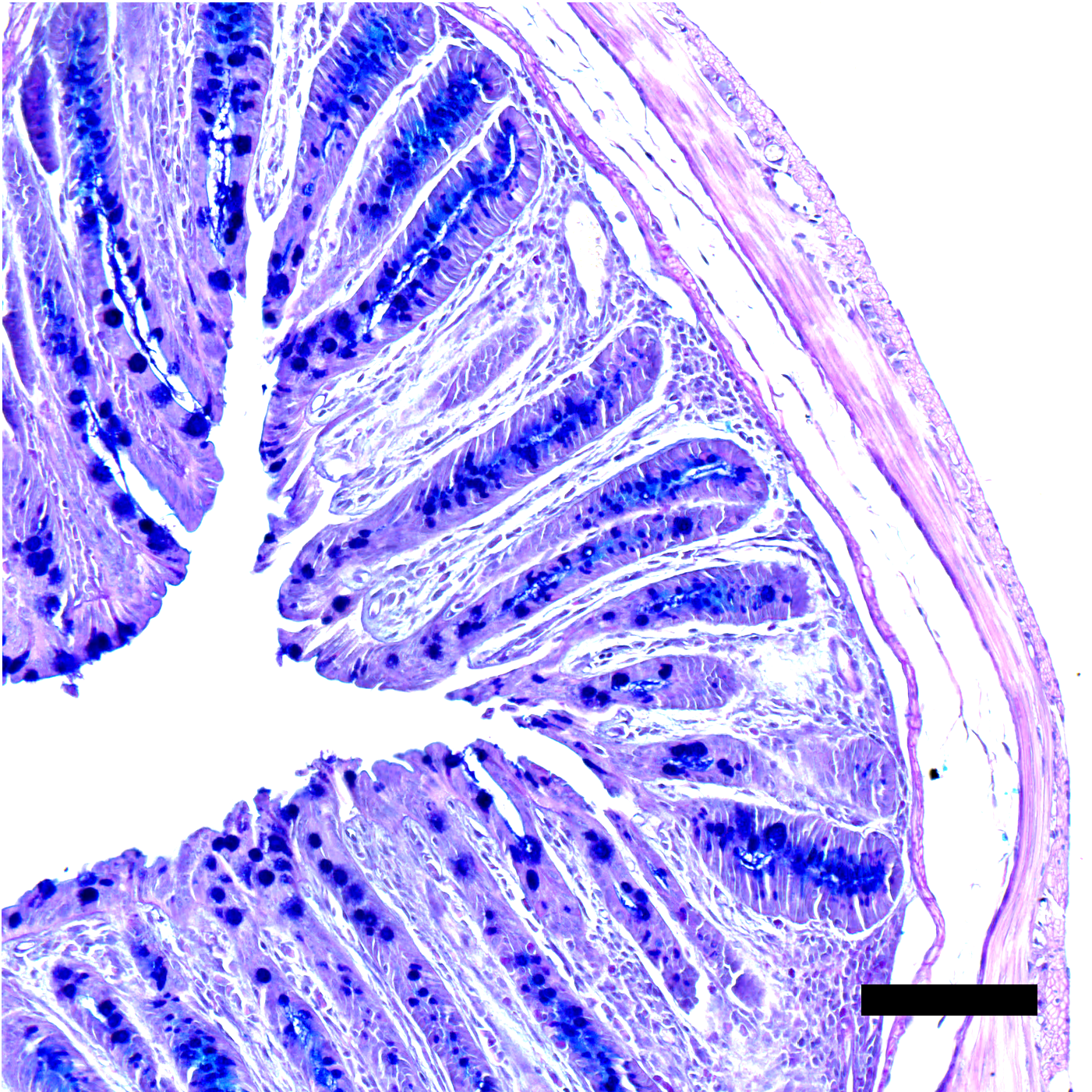

Supplement: Supplementary file 10 — Source data Fig. 7 [file 44321_2025_321_MOESM10_ESM.zip › Figure 7/7F/7F AB PAS WT modified.png]

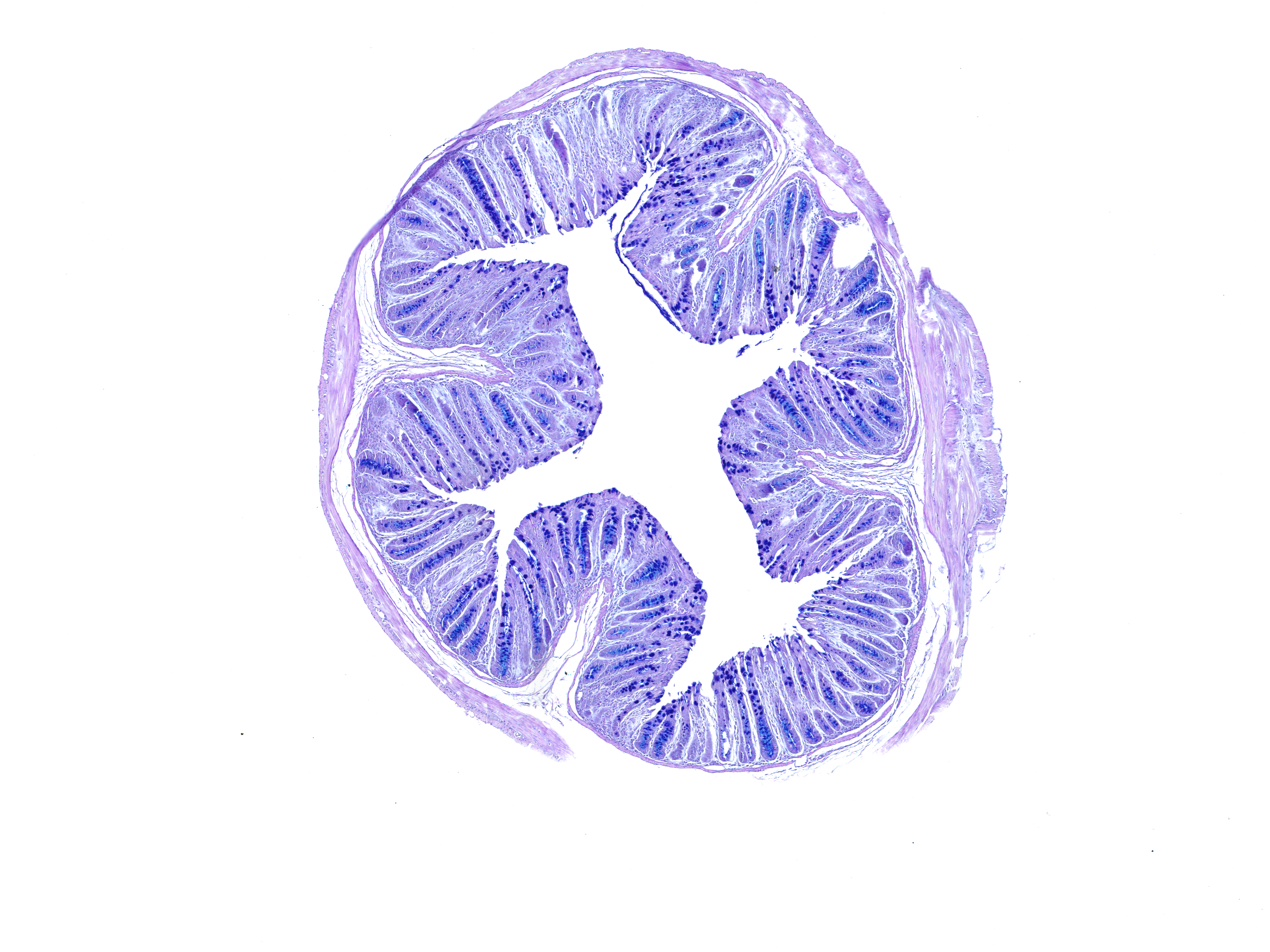

Supplement: Supplementary file 10 — Source data Fig. 7 [file 44321_2025_321_MOESM10_ESM.zip › Figure 7/7F/7F AB PAS WT original.tif]

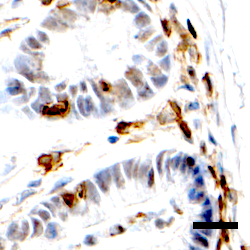

Supplement: Supplementary file 10 — Source data Fig. 7 [file 44321_2025_321_MOESM10_ESM.zip › Figure 7/7F/7F CD45 E138A cropped.png]

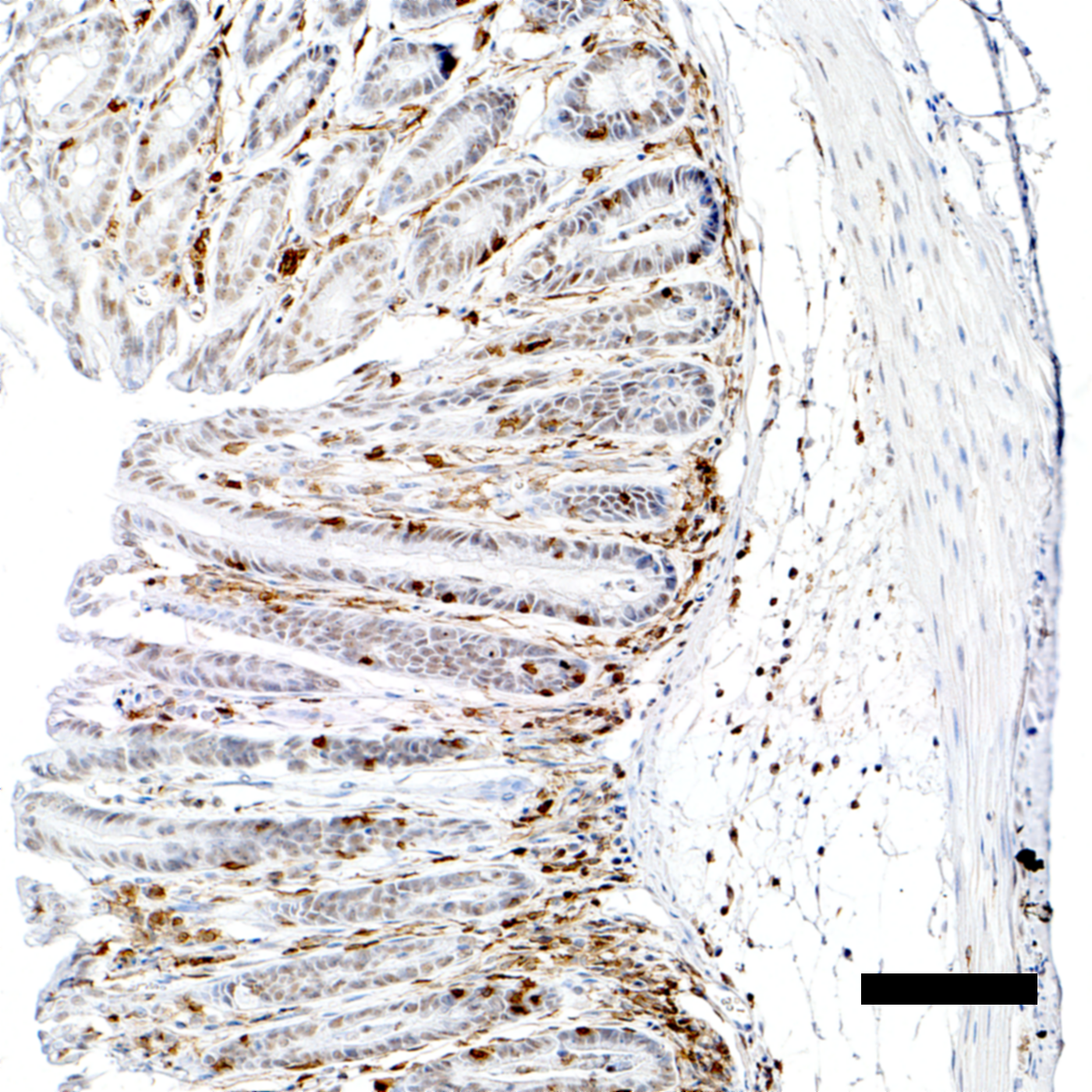

Supplement: Supplementary file 10 — Source data Fig. 7 [file 44321_2025_321_MOESM10_ESM.zip › Figure 7/7F/7F CD45 E138A modified.png]

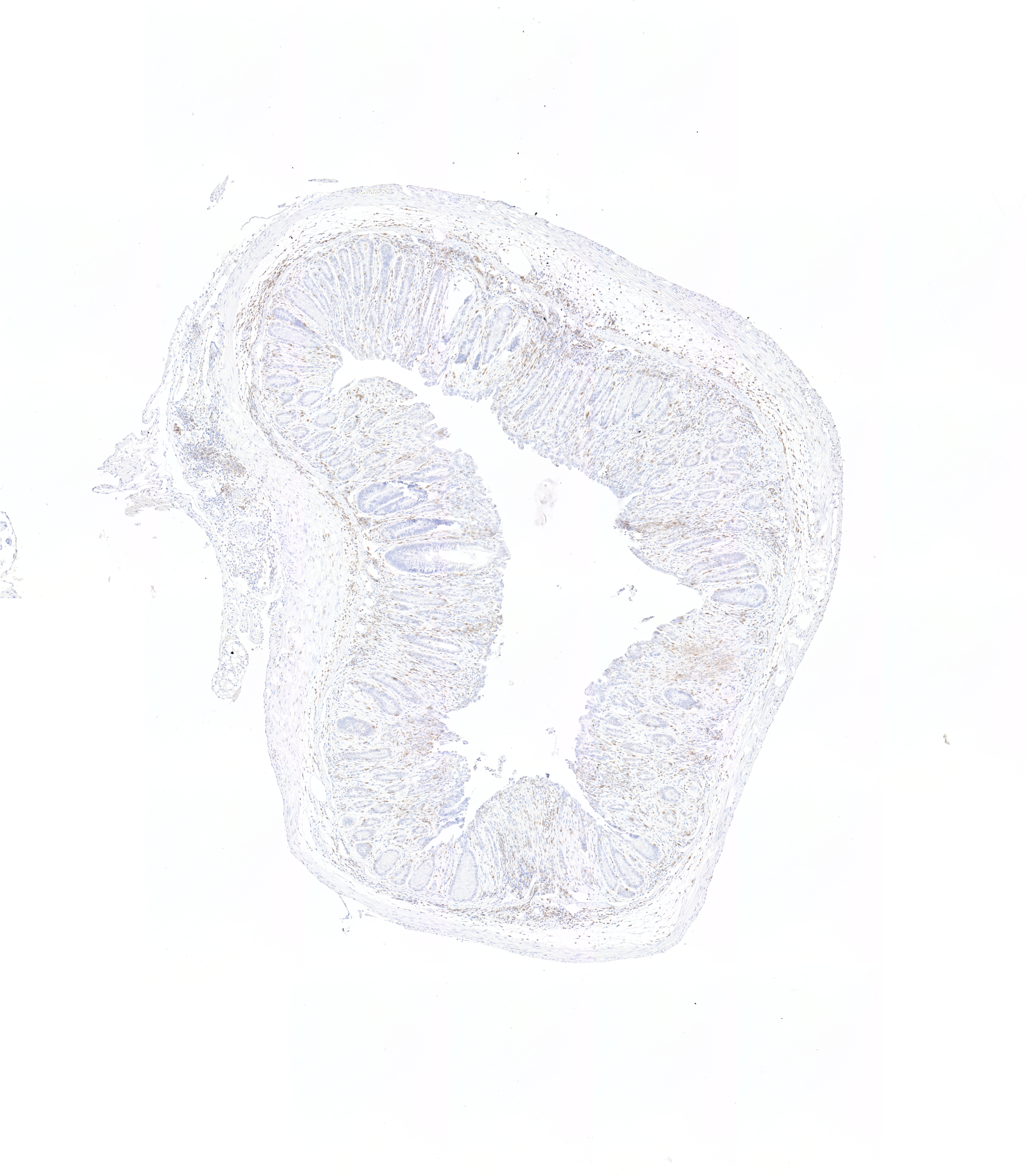

Supplement: Supplementary file 10 — Source data Fig. 7 [file 44321_2025_321_MOESM10_ESM.zip › Figure 7/7F/7F CD45 E138A original.tif]

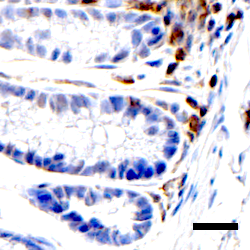

Supplement: Supplementary file 10 — Source data Fig. 7 [file 44321_2025_321_MOESM10_ESM.zip › Figure 7/7F/7F CD45 WT cropped.png]

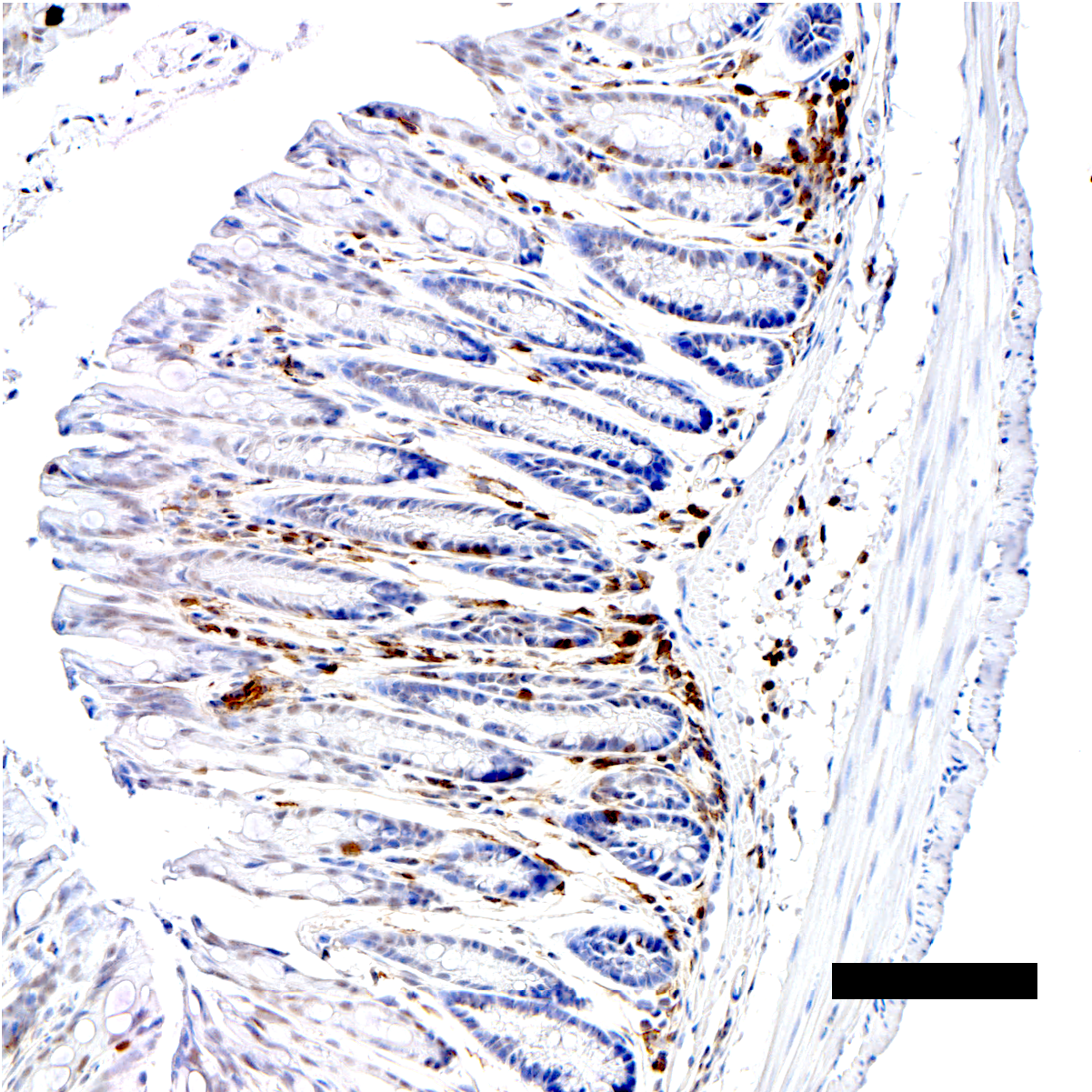

Supplement: Supplementary file 10 — Source data Fig. 7 [file 44321_2025_321_MOESM10_ESM.zip › Figure 7/7F/7F CD45 WT modified.png]

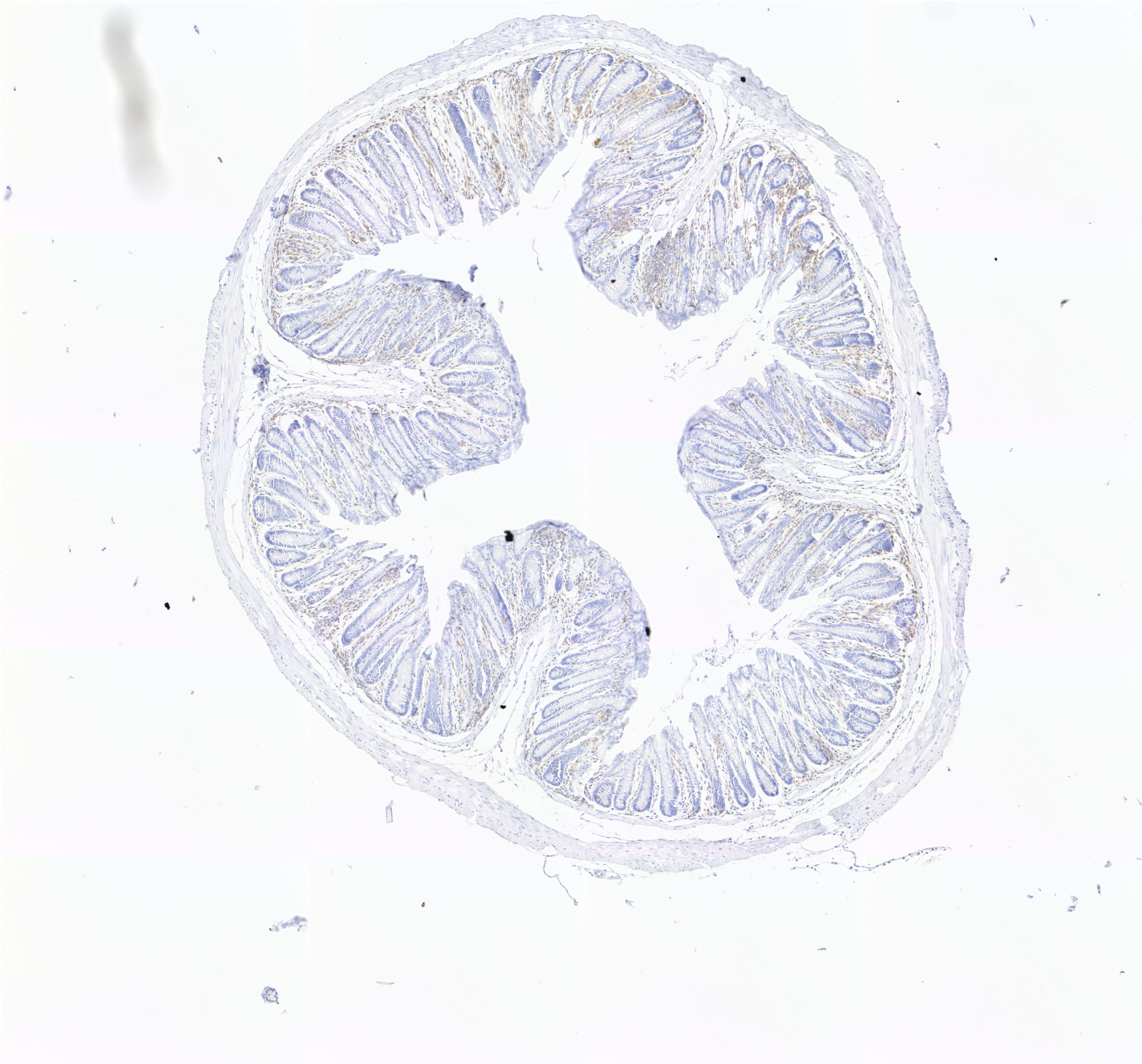

Supplement: Supplementary file 10 — Source data Fig. 7 [file 44321_2025_321_MOESM10_ESM.zip › Figure 7/7F/7F CD45 WT original.tif]

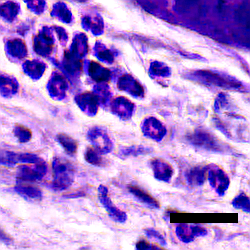

Supplement: Supplementary file 10 — Source data Fig. 7 [file 44321_2025_321_MOESM10_ESM.zip › Figure 7/7F/7F H&E E138A cropped.png]

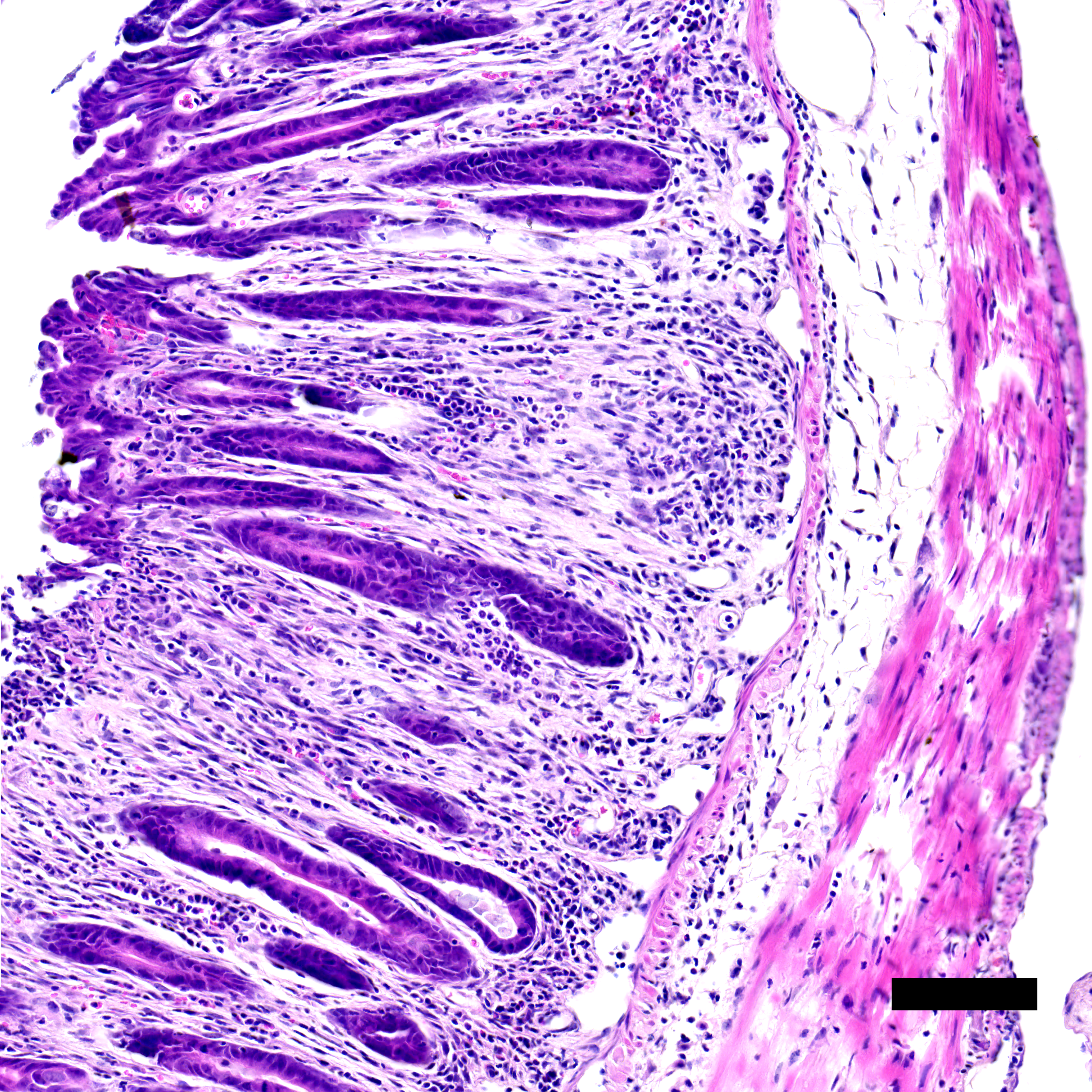

Supplement: Supplementary file 10 — Source data Fig. 7 [file 44321_2025_321_MOESM10_ESM.zip › Figure 7/7F/7F H&E E138A modified.png]

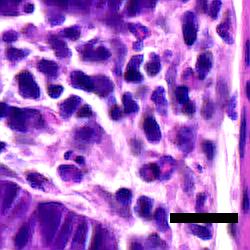

Supplement: Supplementary file 10 — Source data Fig. 7 [file 44321_2025_321_MOESM10_ESM.zip › Figure 7/7F/7F H&E WT cropped.png]

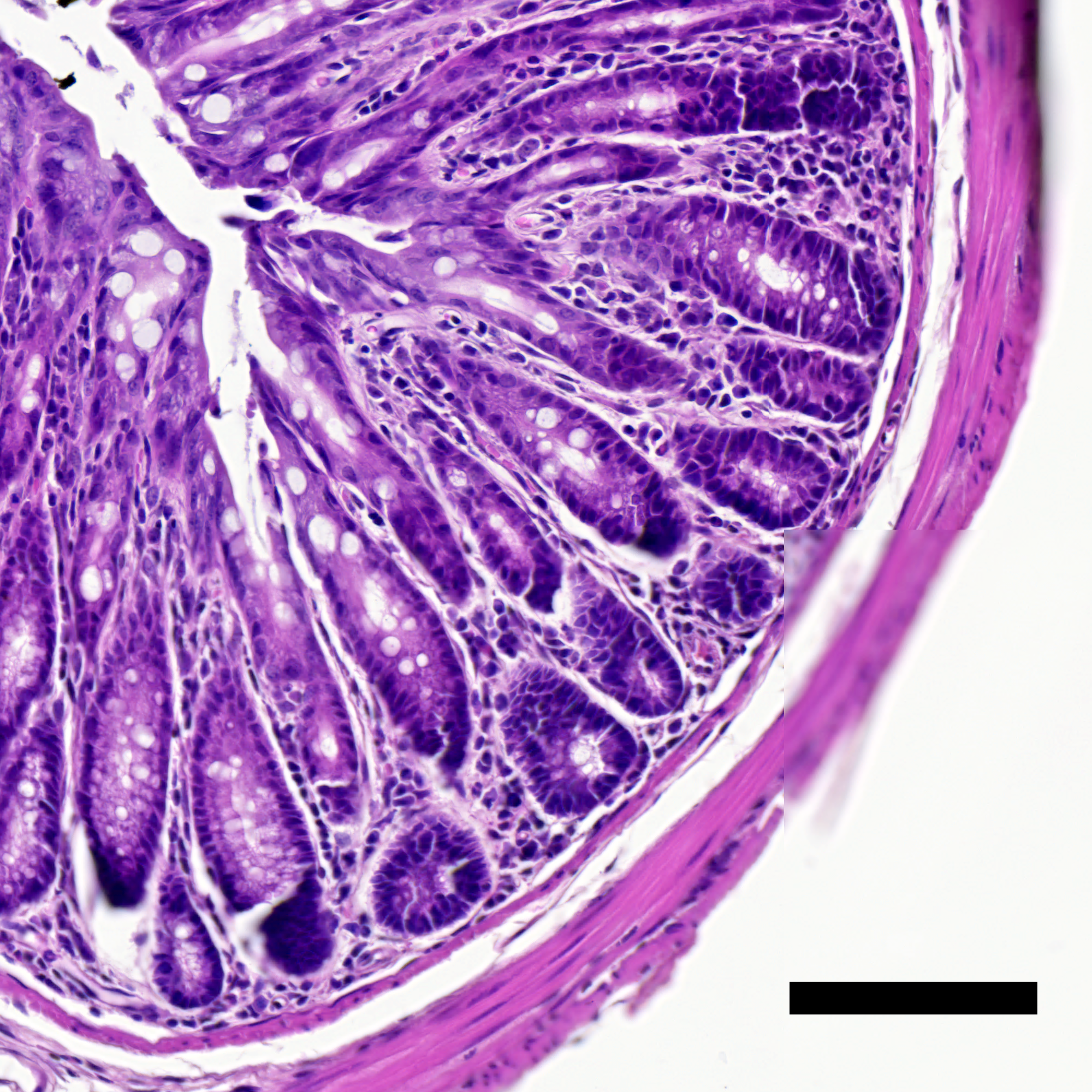

Supplement: Supplementary file 10 — Source data Fig. 7 [file 44321_2025_321_MOESM10_ESM.zip › Figure 7/7F/7F H&E WT modified.png]

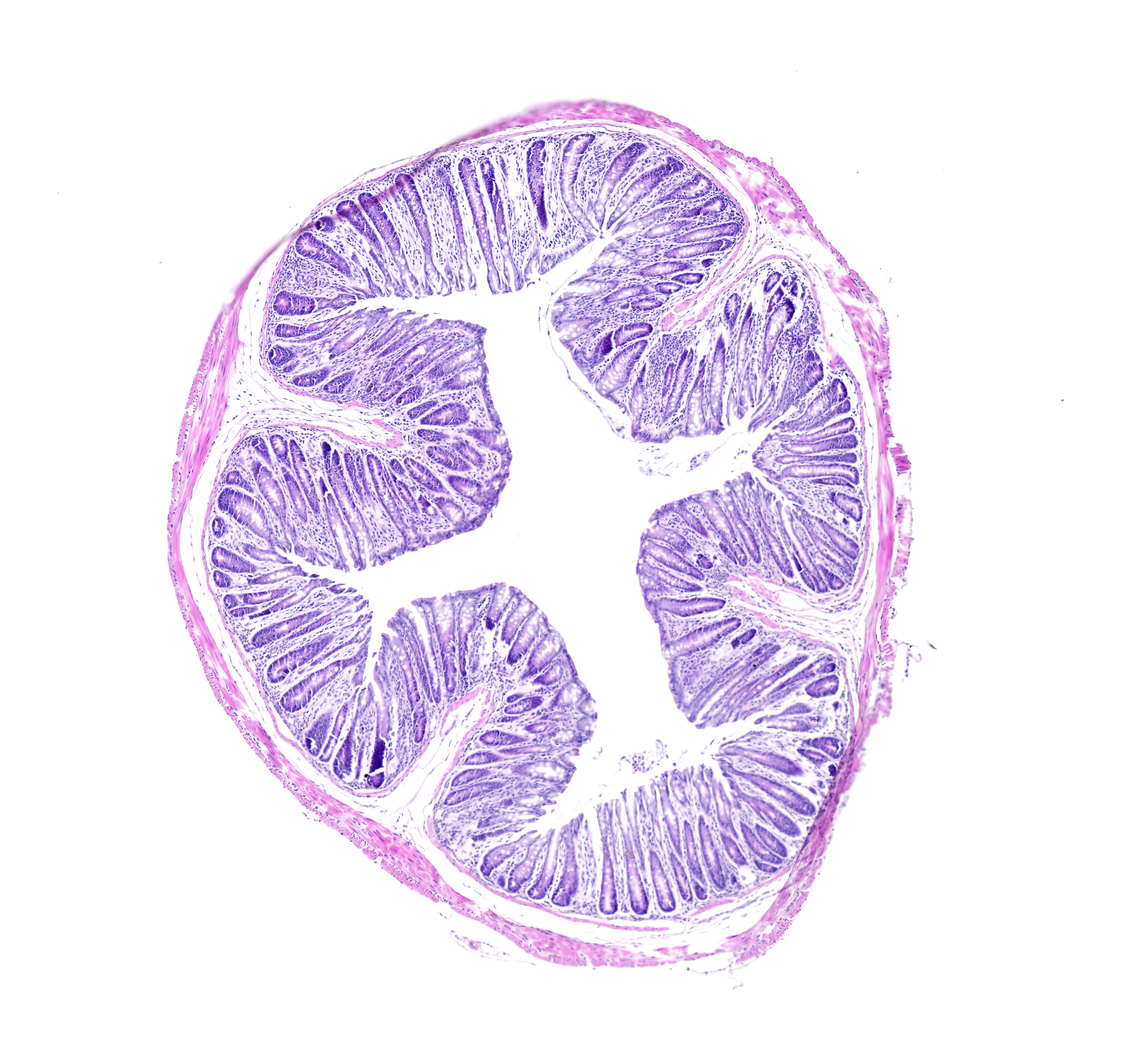

Supplement: Supplementary file 10 — Source data Fig. 7 [file 44321_2025_321_MOESM10_ESM.zip › Figure 7/7F/7F H&E WT original.tif]

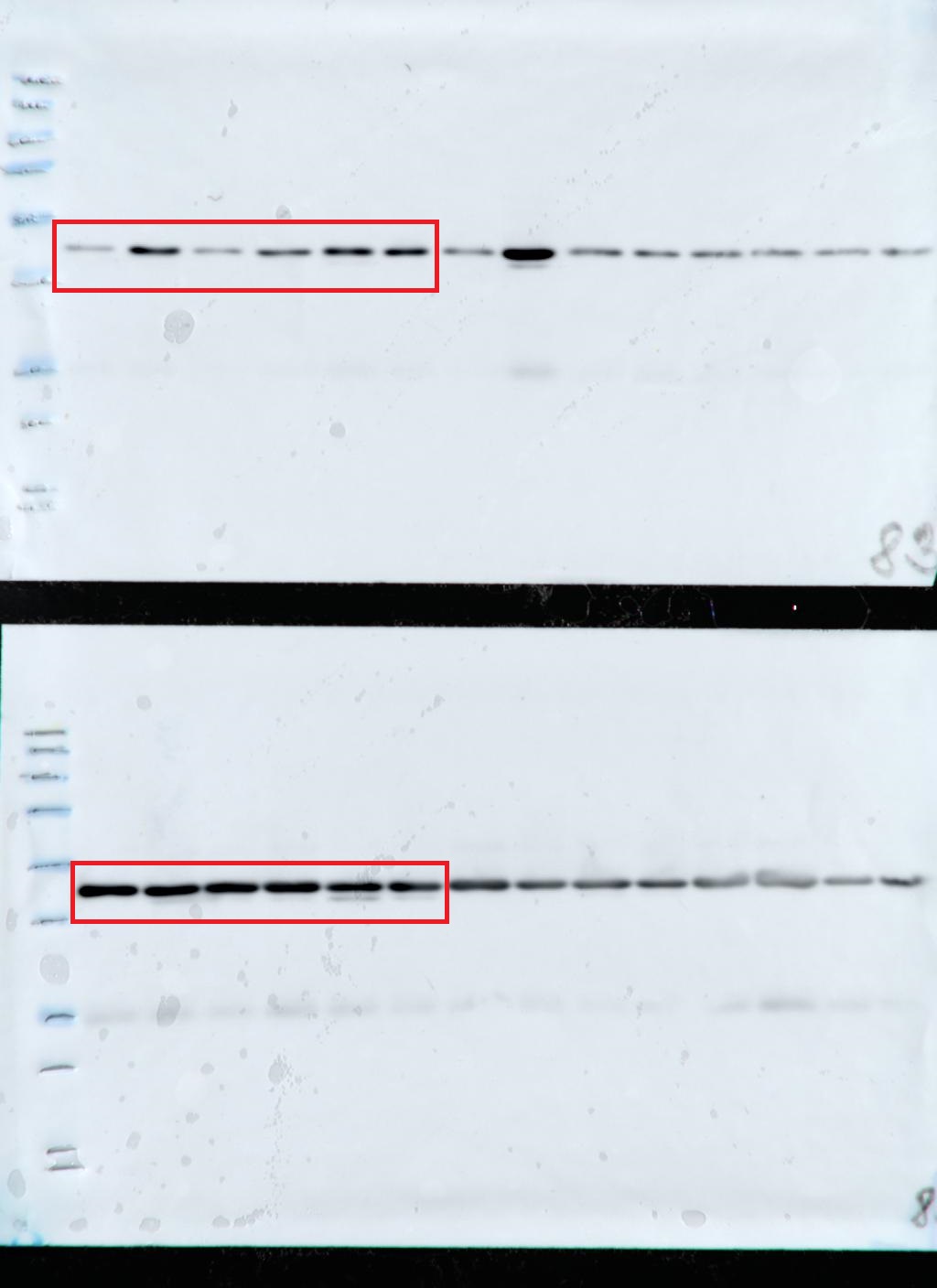

Supplement: Supplementary file 11 — Figure Source Data EV1 [file 44321_2025_321_MOESM11_ESM.zip › Figure EV1/EV1A/EV1A WB Actin original blot for both.jpg]

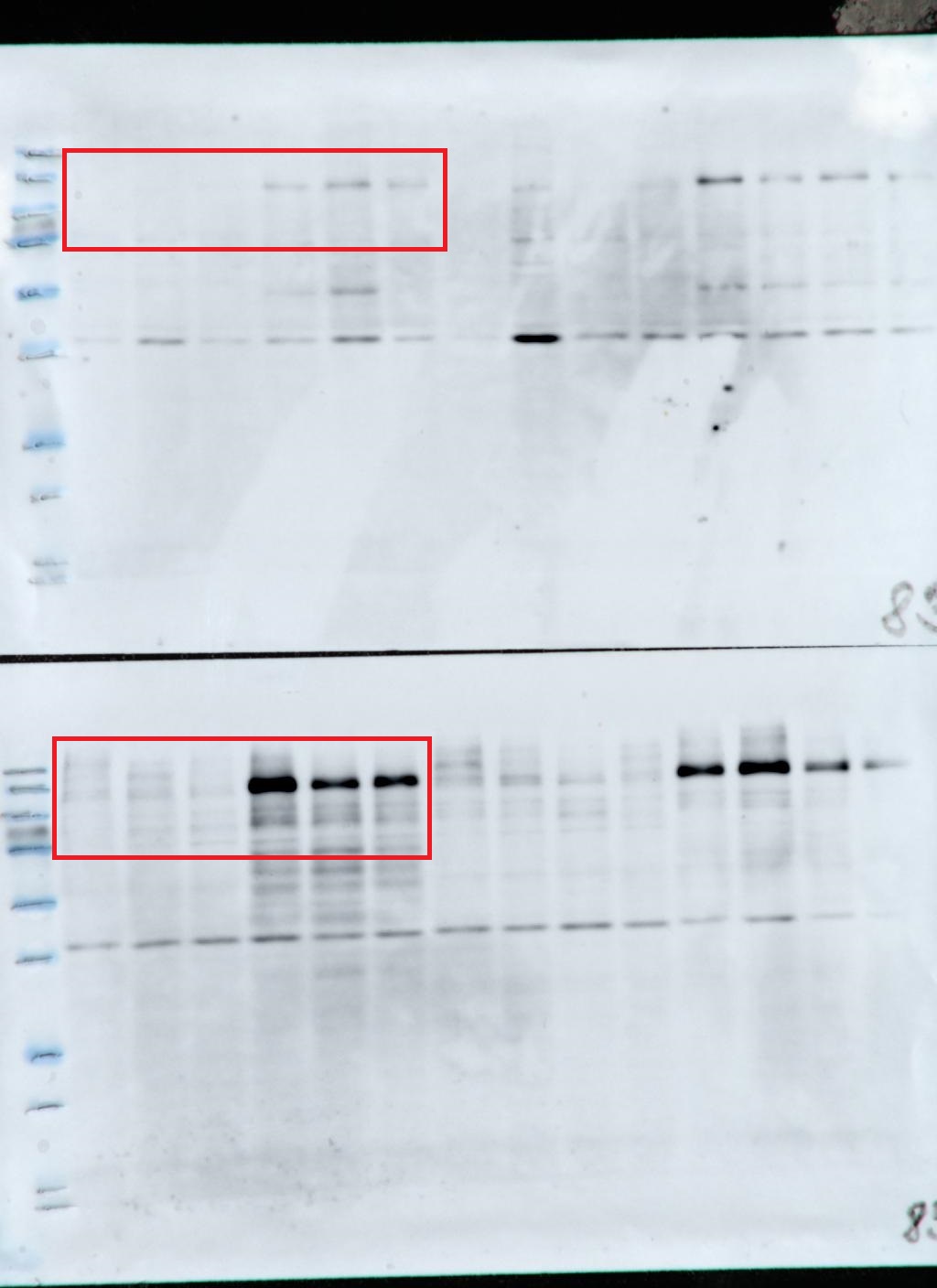

Supplement: Supplementary file 11 — Figure Source Data EV1 [file 44321_2025_321_MOESM11_ESM.zip › Figure EV1/EV1A/EV1A WB CARD14 original blot for both.jpg]
