# Supplementary material for: CARD14 signaling in intestinal epithelial cells induces intestinal inflammation and intestinal transit delay
Source: EMBO Mol Med. 2025 Oct 23;17(12):3300–28. doi: 10.1038/s44321-025-00321-4 (PMC12686530; doi:10.1038/s44321-025-00321-4)
Supplement: Supplementary file 14 — Expanded View Figures [file 44321_2025_321_MOESM14_ESM.pdf]

Expanded View Figures

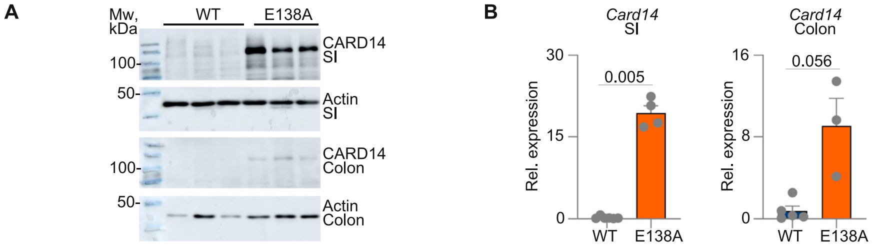

Figure EV1. CARD14 expression in IEC lysates.

(**A**) Representative western blot of CARD14 in IEC lysates of small intestine and colon from control (WT) and *CARD14*(E138A)^{IEC} mice. Actin was used as a loading control. Each lane represents one mouse. (**B**) Relative mRNA expression of human *CARD14* in IEC lysates from the small intestine of WT and *CARD14*(E138A)^{IEC} mice. Data are presented as mean ± SEM; each symbol represents one mouse. Statistical analysis was performed using a Mann–Whitney *U* test with multiple comparisons (**B**). One representative experiment of two independent experiments is shown. Source data are available online for this figure.

 © The Author(s)

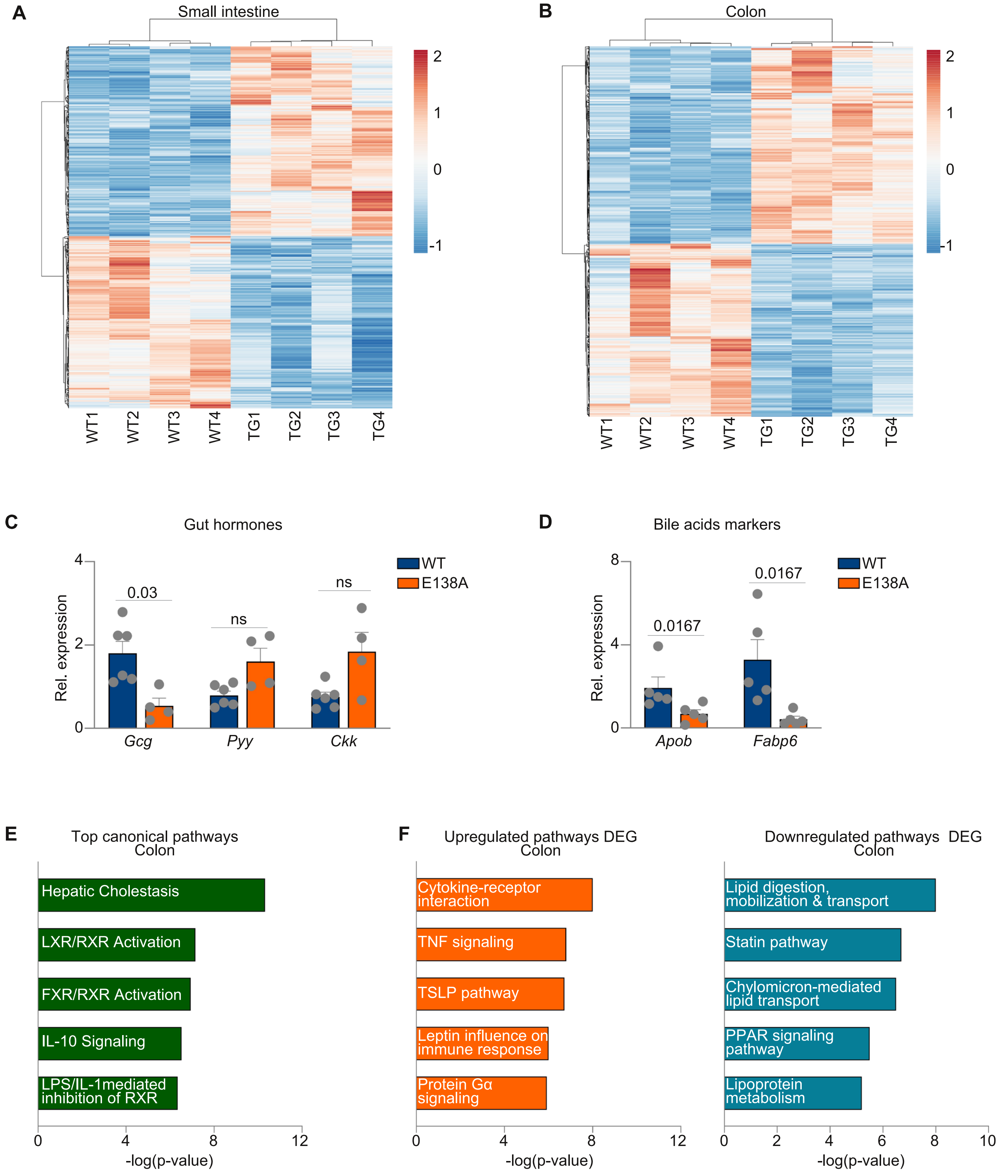

A
Small intestine
2
1
0
-1
WT1
WT2
WT3
WT4
TG1
TG2
TG3
TG4
B
Colon
2
1
0
-1
WT1
WT2
WT3
WT4
TG1
TG2
TG3
TG4
C
Gut hormones
Rel. expression
0
2
4
0.03
ns
ns
WT
E138A
Gcg
Pyy
Ckk
D
Bile acids markers
Rel. expression
0
4
8
0.0167
0.0167
WT
E138A
Apob
Fabp6
E
Top canonical pathways
Colon
Hepatic Cholestasis
LXR/RXR Activation
FXR/RXR Activation
IL-10 Signaling
LPS/IL-1mediated inhibition of RXR
0
4
8
12
-log(p-value)
F
Upregulated pathways DEG
Colon
Cytokine-receptor interaction
TNF signaling
TSLP pathway
Leptin influence on immune response
Protein Gα signaling
0
4
8
12
-log(p-value)
Downregulated pathways DEG
Colon
Lipid digestion, mobilization & transport
Statin pathway
Chylomicron-mediated lipid transport
PPAR signaling pathway
Lipoprotein metabolism
0
2
4
6
8
10
-log(p-value)

Figure EV2. Transcriptional changes in IEC of *CARD14*(E138A)^{IEC} mice.

(**A**, **B**) Heatmaps representing differentially expressed genes (DEGs) in IEC from the small intestine (**C**) (1289 DEGs) and colon (**D**) (1626 DEGs) of WT and *CARD14*(E138A)^{IEC} mice ($n = 4$, each group). (**C**, **D**) Relative mRNA expression of gut hormones (**C**) and bile acid metabolism-related genes (**D**) in colonic IEC. (**E**) Top canonical pathways enriched in DEGs in colon IEC from WT and *CARD14*(E138A)^{IEC} mice, analyzed by Ingenuity Pathway Analysis. (**F**) Gene ontology enrichment analysis of biological processes for DEGs in colon IEC isolated from WT and *CARD14*(E138A)^{IEC} mice, using Enrichr. Data are presented as mean ± SEM; each symbol represents one mouse. Heatmaps were generated in ClustVis from normalized counts of DEGs. Values are displayed as z-scores across each gene, no additional statistical tests were applied (**A**, **B**). Statistical analysis was performed using a Mann–Whitney *U* test with multiple comparisons (**C**, **D**), a Fisher's exact test (**E**, **F**). Data are from one independent experiment, except for (**C**) which includes data from two independent experiments. Source data are available online for this figure.

© The Author(s)

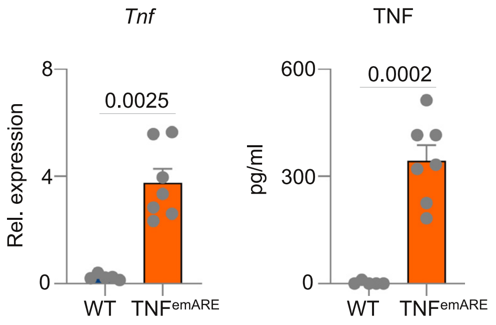

Figure EV3. Relative mRNA expression and protein levels of TNF of ileal lysates from control (WT) and TNF^{emARE} mice.

Data are presented as mean ± SEM; each symbol represents one mouse. Statistical analysis was performed using a Mann–Whitney *t* test (relative *Tnf* gene expression) or unpaired *t* test with Welch correction (TNF protein level). One representative experiment of two independent experiments is shown. Source data are available online for this figure.

© The Author(s)
